# Safety and efficacy of flow diversion for blood blister aneurysms: A comprehensive systematic review and meta-analysis

**DOI:** 10.1007/s10143-026-04278-x

**Published:** 2026-04-25

**Authors:** S. Farzad Maroufi, Mohammad Sadegh Fallahi, Ajith J. Thomas, Daniel A. Tonetti

**Affiliations:** 1 Neurosurgical Research Network (NRN), Universal Scientific Education and Research Network (USERN), Tehran University of Medical Sciences, Tehran, Iran; 2 Department of Neurosurgery, Johns Hopkins University, Baltimore, MD USA; 3 Cooper Medical School of Rowan University, Camden, NJ USA; 4 Division of the Cooper Neurological Institute, Department of Neurosurgery, Cooper University Health Care, Camden, NJ USA

**Keywords:** Blood Blister Aneurysm, Flow Diversion, Pipeline Embolization Device, Systematic Review

## Abstract

Blood blister aneurysms (BBAs) are surgically challenging with a high risk of bleeding and recurrence. Endovascular techniques, particularly flow diverters (FDs), offer a less invasive alternative to open surgery and conventional endovascular techniques by endovascular reconstruction and reducing aneurysm sac manipulation. This review aims to evaluate the safety and efficacy of FDs in treating BBAs, considering factors affecting outcomes. A systematic review of PubMed, Scopus, and Embase was performed aiming at studies reporting outcomes of FDs for BBAs. All reported radiologic, clinical, and complication outcomes were meta-analytically pooled. A total of 39 studies including 511 patients with BBAs treated with FDs were identified. The average patient age was 50.55 years, with 65.99% female, and 83.12% of aneurysms located in the internal carotid artery. Most aneurysms had ruptured before treatment, with an average maximum diameter of 2.94 mm. The mean time from symptom onset to FD treatment was 5.44 days, with Pipeline Embolization Devices used most frequently. The overall complete aneurysm occlusion rate was 87.56%, and 84.05% of patients had a good neurological outcome (mRS ≤ 2). In the subgroup analysis of large multicentric studies, the complete occlusion and good neurological outcome rates were 87.14% and 81.95%, respectively. Complications included intra-operative rupture (1.60%), hemorrhagic (5.62%), thromboembolic (6.89%), and neurologic (6.30%) complications, with a mortality rate of 6.55%. Older age and higher subarachnoid hemorrhage grades were associated with worse outcomes following flow diversion. This review demonstrates that FDs are safe and effective for treating ruptured BBAs, achieving promising rates of complete aneurysm occlusion and favorable neurological outcomes. However, complications such as intra-operative rupture and thromboembolic events still persist.

**Supplementary Information:**

The online version contains supplementary material available at 10.1007/s10143-026-04278-x.

## Introduction

 Blood blister aneurysms (BBAs) are rare and surgically challenging entities [1]. Characterized by their ill-defined neck and fragile arterial walls resulting from dissection, these lesions predominantly arise in the non-branching segments of the supraclinoid internal carotid artery (ICA) or the intradural vertebral artery (V4 segment) [2]. The inherent risk of intraoperative bleeding and recurrence contributes to the generally poor prognosis associated with BBAs [3].

Recent literature increasingly supports endovascular reconstructive techniques, such as stents and stent-assisted coiling, over open microsurgical repair for aneurysm treatment. Flow diverters (FDs) have become promising alternatives for managing complex lesions [3]. FDs, placed across the aneurysm neck, promote thrombosis by diverting blood flow and encouraging neo-endothelialization. This method offers a less invasive and potentially safer treatment for BBAs, reducing the need for aneurysm sac manipulation and the risk of catastrophic bleeding. However, the use of FDs remains controversial due to challenges like perioperative antiplatelet therapy management, delayed rupture risks, and the long-term durability of aneurysm occlusion. This review aims to evaluate safety and efficacy of FDs in BBAs and identify predictors of outcomes.

## Methods

### Search strategy

This systematic review was conducted in accordance with the PRISMA guidelines. PubMed, Scopus, and Embase were comprehensively searched on April 24, 2024, using keywords related to “blood blister aneurysms” (Supplementary Table 1). The search was updated in PubMed on December 23, 2025. This systematic review was not previously registered and did not include a search of gray literature.

### Study selection

Two independent reviewers screened the titles/abstracts of all identified records. Subsequently, full-text was assessed according to the inclusion and exclusion criteria. Any discrepancies were resolved through consensus or consultation with a third reviewer. To identify additional records, a manual inspection of the reference list of included articles was performed. Additionally, we screened for potential overlapping cohorts using study site(s), recruitment periods, registry identifiers (when available), and author groups. Where overlap was suspected, we prioritized the most comprehensive dataset and excluded likely duplicates to avoid double-counting.

### Eligibility criteria

Inclusion criteria were: (1) Peer-reviewed original research articles, (2) Use of FDs for the treatment of BBAs, (3) Reporting outcomes such as occlusion rates, complication rates, recurrence rates, or clinical outcomes, and (4) Trials, cohorts, case-controls, and case series. Studies were excluded if: (1) Did not focus on the use of FDs for the treatment of BBAs, (2) Were review articles, editorials, letters, or conference abstracts, (3) Were published in non-English, or (4) Did not report relevant outcomes.

### Data extraction

The same two reviewers extracted data from the included studies using a standardized data extraction form. A third reviewer checked the extracted data for validity. Data included study characteristics (author, year, study design), patient demographics, aneurysm characteristics, treatment details, outcomes (e.g., occlusion rates, complication rates, recurrence rates), and follow-up duration. Any discrepancies were resolved through discussion. The definitions of BBAs and the occlusion criteria used in each study are provided in Supplementary Table 2.

### Risk of bias assessment

The Joanna Briggs Institute (JBI) checklist for case series was used to assess the risk of bias by two reviewers. A third reviewer was involved in cases of disagreement. The risk of bias was considered low if the assessed study answered positively to all of the questions in the checklist.

### Statistical analysis

R studio (version 4.1.2, R Foundation for Statistical Computing) and the “meta” package were used for meta-analysis. Proportions were pooled using a generalized linear mixed model (GLMM) with a logit transformation. For continuous outcomes summarized as single means, pooled estimates were obtained using random-effects models with Hartung–Knapp adjustment and accompanying prediction intervals. Influential factors were determined through rigorous meta-regression analysis. For outcomes with high heterogeneity, we used a random-effects model to report the relevant effect estimates. Funnel plots were visually inspected for the presence of asymmetry and the corresponding publication bias. Egger’s test and Begg’s test were used for the assessment of the small study effect. Leave-one-out analyses were conducted for each primary outcome to evaluate how individual studies affected the overall reported rate. Subgroup analysis was performed to compare the outcomes of interest between various subgroups. To address potential confounding, and find the source of study-level heterogeneity, we performed multivariable meta-regression (random-effects) including clinically relevant study-level covariates: age, hemorrhage severity, rupture-to-treatment timing and FD device type. For the multivariable meta-regression, a continuity correction of 0.5 was applied only to studies with zero events. P-value < 0.05 was considered statistically significant.

## Results

**Fig. 1:**
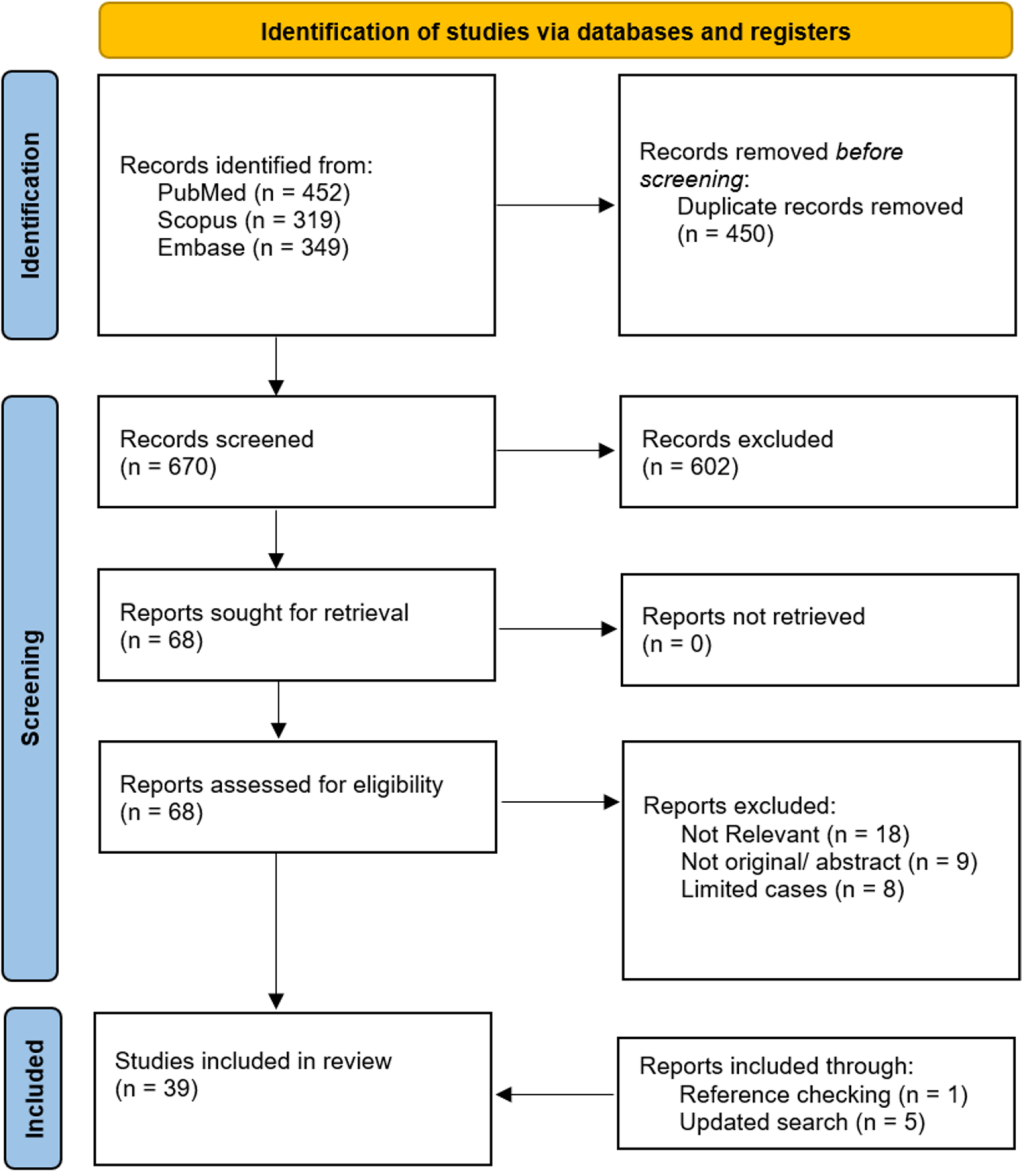
Flow diagram of the screening process. Data added to the PRISMA template (from Page MJ, McKenzie JE, Bossuyt PM, Boutron I, Hoffmann TC, Mulrow CD, et al. The PRISMA 2020 statement: an updated guideline for reporting systematic reviews.BMJ. 2021;372:n71) under the terms of the Creative Commons Attribution (CC BY 4.0) License (https://creativecommons.org/licenses/by/4.0/)

Our systematic search identified a total of 1,120 records. After removing duplicates, 670 articles remained for title and abstract screening. Of these, we assessed the full text of 68 articles for eligibility. One additional study was included through reference checking. Following the updated search, five additional studies were included. A total of 39 studies were ultimately included for quantitative analysis (Fig. 1) [3–41].

The included studies were retrospective case series and cohort studies published between 2013 and 2025. The majority of studies originated from the United States (10 studies) and China (8 studies). In total, our analysis included data from 511 patients with BBA who underwent FD treatment (Table 1).

**Table 1:** Characteristics of included studies

Author, year	Country	Number of patients	Mean age	Aneurysm location (Number of aneurysms)	FD device	Number of FDs per aneurysm (Number of aneurysms)	Antiplatelet/anticoagulation regimen	Occlusion imaging criteria
Çinar, 2013 [4]	Turkey	7	44.5	ICA	PED	1 (11), 2 (1)	Aspirin/Clopidogrel (6), Aspirin/Ticagrelor (1)	NA
Chalouhi, 2014 [5]	USA	8	55.0	ICA (7), BA (1)	PED	NA	Aspirin/Clopidogrel	NA
Yoon, 2014 [3]	USA	11	44.8	L ICA (6), R ICA (2), L Comm (2)	PED	1 (4), 2 (1)	Aspirin/Clopidogrel	NA
Aydin, 2015 [6]	Turkey	11	50.2	ICA (9), BA (2)	Silk	1 (5), 2 (3)	Aspirin/Clopidogrel	RROC
Lin, 2015 [7]	USA	8	NA	ICA (7), PCom (1)	PED	1 (41), 2 (2)	Aspirin/Clopidogrel	RROC
Cerejo, 2017 [8]	USA	8	51.9	ICA	PED/PED Flex	1	Aspirin/Clopidogrel	NA
Linfante, 2017 [10]	USA	10	47.2	ICA (8), MCA (2)	PED	1	Aspirin/Clopidogrel	RROC
Luecking, 2017 [11]	Germany	7	NA	NA	FRED	NA	Aspirin/Clopidogrel	RROC
Ryan, 2017 [12]	USA	13	54.8	L ICA (10). R ICA (6)	PED	1	Aspirin/Clopidogrel	NA
Yang, 2017 [13]	Hungary	12	50.2	NA	PED	1	Aspirin/Clopidogrel	RROC
Kumar, 2017 [9]	India	6	53.2	PC ICA (4), SC ICA (2)	PED (2), FRED (3), SURPASS (1)	1 (4), 2 (2)	Aspirin/Ticagrelor	NA
Hellstern, 2018 [14]	Germany	5	55.6	ICA (3), ACA (1), ACom (1)	p64	1	Aspirin/(Ticagrelor OR Prasugrel)	NA
Lozupone, 2018 [15]	Italy	8	53.4	ICA (4), BA (2), MCA (1), PCA (1)	PED (7), Silk (1)	1	Aspirin/Ticlopidine	NA
Mokin, 2018 [16]	USA	43	52.8	NA	PED	1	Aspirin/Clopidogrel (37), prasugrel or ticagrelor (6)	NA
Parthasarathy, 2018 [17]	India	9	53.3	R SC ICA (6), L SC ICA (3)	PED	1 (3), 2 (3)	Ecosprin/Prasugrel	NA
Ghorbani, 2019 [18]	Iran	6	47.5	ICA	PED (4), Silk Plus (2)	1 (6)	Aspirin/Clopidogrel	NA
Griffin, 2019 [19]	USA	8	NA	NA	PED	1 (18), 2 (1)	Aspirin/Clopidogrel	NA
Capocci, 2020 [20]	France	8	52	ICA (5), ACA (1), BA (1), PCA (1)	PED (6), Surpass (2)	NA	Aspirin/Ticagrelor	OKM
Incandela, 2020 [21]	Italy	6	42.7	ICA (3), MCA (1), BA (2)	PED (3), FRED (2), DERIVO (1)	1	Aspirin/Clopidogrel	NA
Möhlenbruch, 2020 [22]	Germany, Turkey, Austria	30	55.6	ICA (19), BA (7), VA (2), ACom (1), PCA (1)	FRED	1	Aspirin/Clopidogrel (21), Ticagrelor (5), Prasugrel (4)	OKM
Zhang, 2020 [23]	China	10	50.7	L ICA (4), R ICA (6)	PED	1	Aspirin/Clopidogrel	RROC
Aboukais, 2021 [24]	France	8	52.6	ICA	PED (4), Silk (4)	1 (5), 2 (1)	Aspirin/Clopidogrel	NA
Gopinathan, 2021 [25]	Singapore	6	58.7	ICA (4), ACA (2)	PED (5), Silk Vista (1)	NA	Aspirin/Ticagrelor	OKM
Tanburoglu, 2021 [26]	Turkey	6	41.7	SC ICA	PED (4), Derivo (1), SURPASS (1), FRED (1)	1	Aspirin/Ticagrelor (1), Prasugrel (5)	OKM
Wang, 2021 [27]	China	6	48.3	L ICA C6 (2), L ICA C7 (2), R ICA C7 (2)	PED Flex	1 (10), 2 (2)	Aspirin/Clopidogrel	RROC
Zhang, 2021 [28]	China	19	53.1	SC ICA (19)	PED	1 (11), 2 (1)	Aspirin/Clopidogrel	NA
Zhong, 2021 [29]	China	12	NA	NA	PED	NA	Aspirin/Clopidogrel	OKM
Alpay, 2022 [30]	Finland	47	NA	NA	NA	1 (4), 2 (1)	Aspirin/Prasugrel	OKM
Liu, 2022 [31]	China	12	44.3	R ICA C7 (3), L ICA C7 (4), R ICA C6 (2), L ICA C6 (3)	PED	1 (5), 2 (3)	Aspirin/Clopidogrel	RROC
Feng, 2023 [32]	China	8	48.5	ICA	Turbridge	1 (41), 2 (2)	Aspirin/Ticagrelor	RROC
Madjidyar, 2023 [33]	Switzerland	6	55.8	ICA (3), VA (1), BA (1), PCA (1)	PED-Shield	1 (9)	SAPT with Prasugrel or Aspirin	NA
Reidy, 2023 [34]	Australia	15	52.2	R ICA (7), L ICA (6), L M2 (1), Mid Basilar trunk (1)	PED	1	Aspirin/Prasugrel (10), Aspirin/Clopidogrel (3)	NA
Zhang, 2023 [35]	China	10	41.7	L ICA C6 (2), L ICA C7 (1), R ICA C6 (4), R ICA C7 (3)	PED	NA	Aspirin/Clopidogrel	RROC
Hoffman, 2024 [36]	USA	12	49.8	ICA	PED (1), PED Flex (11)	1	Aspirin/Clopidogrel (11), Aspirin/Ticagrelor (1)	RROC
Chen, 2025 [38]	China	31	49.3	ICA	PED	1	Aspirin/Clopidogrel	NA
Chen, 2025 [39]	China	13	49.8	L ICA (5), R ICA (8)	PED (8), Lattice (5)	NA	Aspirin/Ticagrelor	NA
Chen, 2025 [37]	Taiwan	17	NA	ICA	PED-Shield	1 (16), 2 (1)	SAPT Ticagrelor (16), Prasugrel (1)	NA
Dange, 2025 [40]	India	21	58	R ICA (8), L ICA (9), Basilar trunk (3), ACom (1)	PED (4), Derivo (14), Silk Vista (3)	1	Aspirin/Ticagrelor	NA
Wroe, 2025 [41]	USA	28	54.8	ICA (6), OA (8), M1 (1), ACA (1), PCA (2), BA (3), VA (7)	NA	1 (25), 2 (3)	Aspirin/Clopidogrel (20), Aspirin/Ticagrelor (6), Aspirin/Prasugrel (2)	OKM

*NA* Not available, *ICA* Internal carotid artery, *SC* Supraclinoid, *PC* Paraclinoid, *ACA* Anterior cerebral artery, *MCA* Middle cerebral artery, *PCA* Posterior cerebral artery, *BA* Basilar artery, *VA* Vertebral artery, *ACom* Anterior communicating artery, *PCom* Posterior communicating artery, *M1* M1 segment of the middle cerebral artery, *PED* Pipeline embolization device, *FRED* Flow redirection endoluminal device, *RROC* Raymond–Roy occlusion classification, *OKM* O’Kelly-Marotta grading scale

### Baseline characteristics

The average patient age was 50.55 years, with 65.99% being female. The most frequent location for the aneurysms was the ICA, at 83.12%. Nearly all (98.32%) aneurysms had ruptured before treatment. The mean maximum aneurysm diameter was 2.94 mm. Three grading scales were employed: Fisher, Hunt-Hess, and World Federation of Neurological Surgeons (WFNS) scales. The average scores on these scales were 2.93, 2.24, and 2.19, respectively (Table 2, Supplementary Figs. 1, 2, 3, 4, 5, 6, 7 and 8).

**Table 2:** Meta-analytic details of baseline characteristics

Variables	No Study	No Patients	Value (95% CI)	I^2^	Tau	H
**Baseline Characteristics**						
Age, years	33	412	50.55 (48.90–52.20)	63.8	3.69	1.66
Female%	31	260	65.99 (61.17–70.50)	26.6	0.48	1.17
ICA location%	33	320	83.12 (79.04–86.54)	0.0	2.06	1.00
Ruptured status%	29	351	98.32 (96.31–99.24)	0.0	6.81	1.00
Aneurysm maximum diameter, mm	23	259	2.94 (2.56–3.32)	95.3	0.70	4.60
Fisher grade	20	266	2.93 (2.72–3.15)	72.2	0.38	1.90
HH grade	21	293	2.24 (2.02–2.46)	77.8	0.41	2.12
WFNS grade	9	95	2.19 (1.58–2.80)	76.4	0.61	2.06

*ICA* Internal carotid artery, *HH* Hunt-Hess, *WFNS* World Federation of Neurological Surgeons

### Treatment details

The mean time from symptom onset to FD treatment was 5.44 days (Supplementary Fig. 9). The included studies employed various FD devices, with PEDs (including PED, PED-Flex, and PED-Shield) being the most common (76.08%) (Supplementary Fig. 10), followed by FRED (9.79%). The mean FD length was 21.01 mm (Supplementary Fig. 11). Multiple FDs were required in 8.39% of patients (Supplementary Fig. 12), and adjunctive coiling was performed in 30.10% of patients (Supplementary Fig. 13). Aspirin/Clopidogrel (61.45%) was the most commonly used dual antiplatelet therapy (DAPT) regimen (Supplementary Fig. 14), followed by Aspirin/Prasugrel or Aspirin/Ticagrelor (29.16%) (Table 3).

**Table 3:** Meta-analytic details of endovascular treatment

Variables	No Study	No Patients	Value (95% CI)	I^2^	Tau	H
Time to treatment, days	21	221	5.44 (3.31–7.56)	94.9	3.70	4.44
*FD Device*						
PED (PED-Flex, PED-Shield), %	37	334	76.08 (71.87–79.84)	0.0	4.98	1.00
FRED, %	37	43	9.79 (7.34–12.95)	0.0	15.53	1.00
Silk, %	37	22	5.01 (3.32–7.49)	0.0	5.73	1.00
Other (Derivo, SURPASS, p64, Tubridge)	37	38	8.66 (6.36–11.67)	0.0	4.26	1.00
≥ 2 FDs, %	33	38	8.39 (6.16–11.32)	2.18	1.48	1.00
FD length, mm	7	77	21.01 (18.61–23.40)	84.0	2.34	2.50
Adjunctive coiling, %	36	124	30.10 (25.86–34.70)	7.9	4.93	1.04
*DAPT regimen*						
Aspirin/Clopidogrel, %	39	314	61.45 (57.15–65.57)	0.0	23.46	1.00
Aspirin/Prasugrel or Ticagrelor, %	39	149	29.16 (25.38–33.25)	0.0	19.07	1.00

*PED* Pipeline embolization device, *FRED* Flow re-direction endoluminal device, *DAPT* Dual antiplatelet therapy

### Radiologic & clinical outcomes

**Fig. 2:**
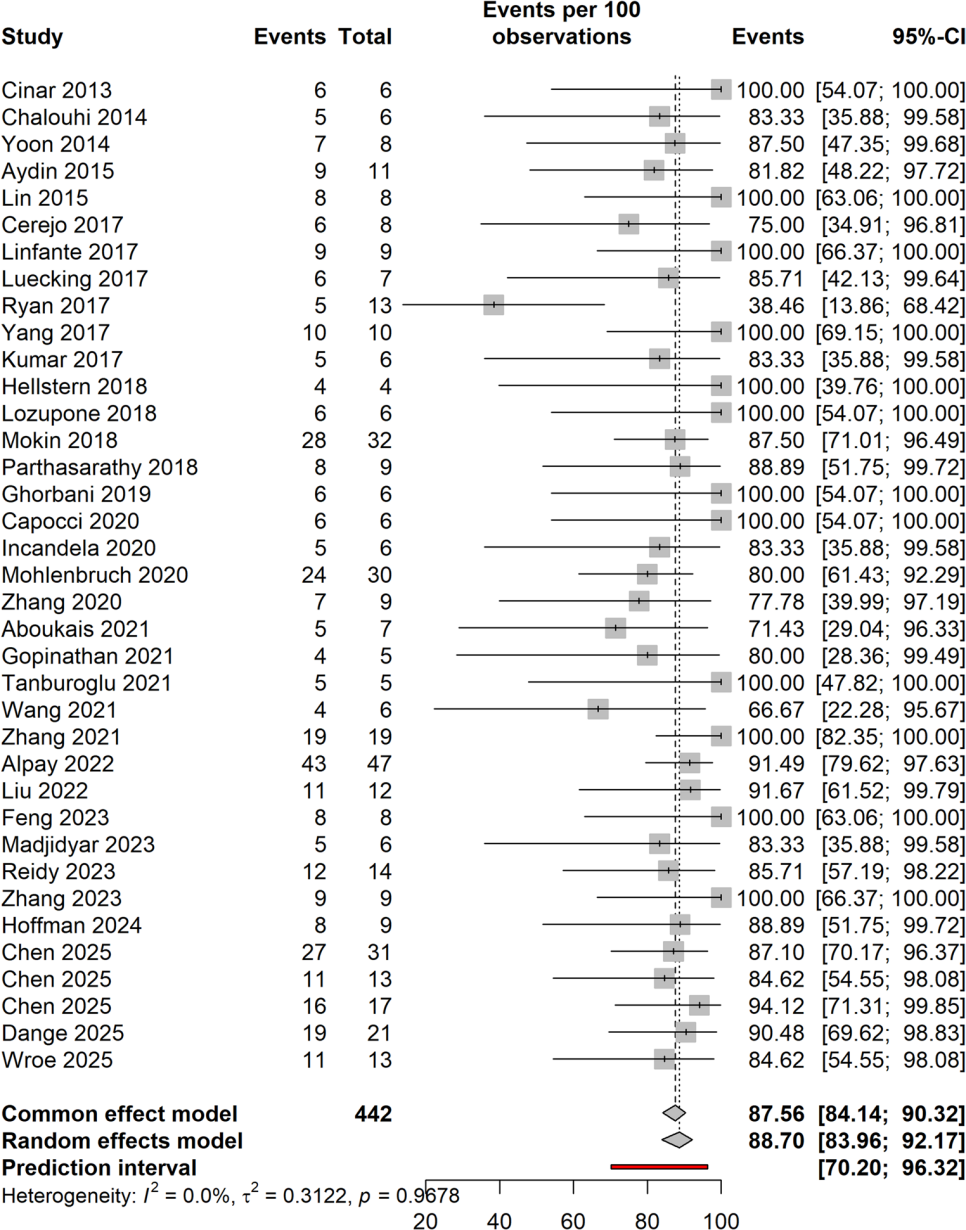
Forest plot demonstrating the proportion of patients with complete aneurysm occlusion at the last follow-up

Patients were followed for a duration ranging from 3 to 59 months, with an average follow-up of 12.72 months (Median: 7.21 months, Supplementary Fig. 15). The pooled rate of complete aneurysm occlusion at the last follow-up was 87.56% (Fig. 2), while 9.73% of patients had remnant aneurysms in studies reporting these rates (Table 4, Supplementary Fig. 16). The pooled rates of complete occlusion and remnant aneurysms did not change on leave-one-out analysis (Supplementary Figs. 17, 18).

**Table 4:** Meta-analytic details of treatment outcomes

Variables	*N* Studies	*N* Patients	Value (95% CI)	I^2^	Tau	H
Follow-up	19	209	12.72 (7.29–18.15)	96.1	9.64	5.05
Complete occlusion, %	37	387	87.56 (84.14–90.32)	0.0	0.56	1.00
Remnant, %	37	43	9.73 (7.29–12.86)	0.0	0.69	1.00
Good neurological outcome (mRS ≤ 2)	34	332	84.05 (80.10- 87.34)	0.0	0.88	1.00
Post-operative CSF diversion, %	16	55	11.77 (3.25–34.63)	61.4	2.15	1.61
Aneurysm retreatment, %	31	17	4.94 (3.09–7.80)	0.0	0.80	1.00
*Complications*						
Intra-operative bleeding, %	32	6	1.60 (0.72–3.51)	0.0	0.00	1.00
Post-operative hemorrhagic events, %	33	24	5.62 (3.80–8.25)	0.0	0.84	1.00
Thromboembolic, %	32	29	6.89 (4.83–9.74)	0.0	0.00	1.00
Vasospasm, %	12	15	11.90 (7.31–18.81)	0.0	1.10	1.00
Neurologic, %	25	17	6.30 (3.95–9.89)	0.0	0.51	1.00
In-stent stenosis, %	20	19	7.17 (4.62–10.97)	0.0	0.00	1.00
Mortality, %	38	33	6.55 (4.69–9.07)	0.0	0.64	1.00

*mRS* Modified rankin scale

A meta-regression analysis revealed that age (*P* = 0.066) had a marginally significant negative correlation with complete occlusion rates (Table 5). In the subgroup analysis, there was no significant difference in complete occlusion between studies that only used PED compared to other FDs (*P* = 0.545), and between studies using Aspirin/Clopidogrel compared to other DAPTs (*P* = 0.586). Additionally, pooled complete occlusion rates did not differ significantly among studies grouped according to the occlusion criteria applied (*P* = 0.72, Supplementary Fig. 19) .

**Table 5:** Meta-regression details for different factors affecting outcomes

Variables	No of studies	Estimate	SE	*P* value	I^2^
**Complete occlusion**					
Year of publication	37	0.0289	0.0530	0.5895	25.97%
Female gender	31	−1.9692	1.1695	0.1030	21.37%
Age	33	−0.0942	0.0495	0.0663	23.71%
ICA	33	−0.8365	0.9536	0.3871	28.30%
Max diameter	26	0.0259	0.2596	0.9214	42.05%
Fisher grade	21	−0.9074	0.4912	0.0803	0.00%
Hunt-Hess grade	21	−0.4635	0.5889	0.4409	41.81%
WFNS grade	9	−0.0367	0.5081	0.9444	0.00%
Ruptured status	28	1.1381	2.8870	0.6966	35.53%
Time to treatment	21	0.1043	0.0710	0.1582	35.03%
PED	35	0.2959	0.5146	0.5692	29.73%
FD ≥ 2	33	−0.6313	1.1617	0.5907	34.83%
FD length	7	−0.1263	0.1298	0.3752	0.00%
Aspirin/Clopidogrel	37	−0.5468	0.4047	0.1853	23.34%
Adjunctive coiling	35	0.3826	0.5426	0.4856	28.80%
**Good neurological outcome (mRS ≤ 2)**					
Year of publication	34	0.0133	0.0672	0.8443	50.93%
Female gender	29	0.2090	1.3535	0.8785	56.24%
Age	31	−0.0701	0.0583	0.2393	51.58%
ICA	31	0.8747	1.1951	0.4701	40.93%
Max diameter	24	0.4315	0.2464	0.0939	0.00%
Fisher grade	19	−0.8568	0.5342	0.1271	35.64%
Hunt-Hess grade	20	−1.3586	0.5798	**0.0308**	33.56%
WFNS grade	8	−0.9003	0.3843	0.0576	0.00%
Ruptured status	26	−2.7487	3.7448	0.4701	40.32%
Time to treatment	19	−0.0524	0.0461	0.2710	16.72%
PED	34	−0.1474	0.6534	0.8229	49.80%
FD ≥ 2	31	−1.8411	1.2915	0.1647	52.63%
FD length	7	−0.2305	0.2294	0.3611	0.00%
Aspirin/Clopidogrel	34	0.3647	0.5047	0.4751	51.32%
Adjunctive coiling	33	1.5756	0.6367	**0.0190**	29.36%
**Intraoperative bleeding**					
Year of publication	32	0.0101	0.1275	0.9372	0.00%
Female gender	27	1.2620	2.8251	0.6589	0.00%
Age	29	−0.0461	0.0991	0.6459	0.00%
ICA	28	8.1834	3.8492	**0.0432**	0.00%
Max diameter	21	0.8205	0.6389	0.2145	0.00%
Fisher grade	19	0.5860	1.3834	0.6772	0.00%
Hunt-Hess grade	18	−0.0668	1.0505	0.9501	0.00%
Ruptured status	24	134.22	42,115,490	1.0000	0.00%
Time to treatment	18	−0.2719	0.2194	0.2333	0.00%
PED	32	3.9074	3.5629	0.4265	0.00%
FD ≥ 2	27	−6.8646	7.5171	0.3699	0.00%
FD length	6	0.2140	0.2672	0.4680	0.00%
Aspirin/Clopidogrel	32	7.0481	6.4470	0.2830	0.00%
Adjunctive coiling	30	2.6757	1.3395	**0.0556**	0.00%
**Post-operative hemorrhage**					
Year of publication	33	0.0632	0.0827	0.4504	32.51%
Female gender	28	1.6010	1.9818	0.4265	41.26%
Age	30	0.1537	0.0950	0.1170	32.18%
ICA	30	−2.8591	0.7087	**0.0004**	0.00%
Max diameter	25	0.2844	0.3170	0.3790	28.72%
Fisher grade	20	1.1264	0.9170	0.2352	37.77%
Hunt-Hess grade	19	2.0889	0.8346	**0.0228**	8.43%
WFNS grade	8	0.4020	0.7700	0.6203	0.00%
Ruptured status	25	72.746	24,128	0.9976	39.58%
Time to treatment	20	−0.0647	0.0992	0.5225	46.59%
PED	31	1.6599	1.6396	0.3198	0.00%
FD ≥ 2	30	0.9923	1.4968	0.5128	35.15%
FD length	6	−0.4409	0.5396	0.4597	0.00%
Aspirin/Clopidogrel	33	0.6002	0.6838	0.3869	36.36%
Adjunctive coiling	31	−0.6548	0.9137	0.4794	40.05%
**Thromboembolic events**					
Year of publication	32	0.0053	0.0551	0.9242	0.00%
Female gender	28	0.8358	1.2887	0.5223	0.00%
Age	30	0.0268	0.0511	0.6043	0.00%
ICA	29	0.2289	0.8541	0.7908	0.00%
Max diameter	22	−0.0788	0.2235	0.7282	0.00%
Fisher grade	20	0.7213	0.6202	0.2601	0.00%
Hunt-Hess grade	18	0.5110	0.5300	0.3493	0.00%
WFNS grade	9	−0.3028	0.8721	0.7386	50.16%
Ruptured status	25	−0.3518	2.8876	0.9041	0.00%
Time to treatment	19	0.0282	0.0510	0.5867	0.00%
PED	31	−0.4474	0.5151	0.3922	0.00%
FD ≥ 2	28	0.4910	1.2548	0.6988	0.00%
FD length	5	0.4160	0.3740	0.3472	28.56%
Aspirin/Clopidogrel	27	−0.1165	0.4556	0.8000	0.00%
Adjunctive coiling	30	−0.3771	0.6018	0.5359	0.00%
**Mortality**					
Year of publication	38	−0.0182	0.0685	0.7921	26.72%
Female gender	31	−0.0680	1.2526	0.9571	20.25%
Age	33	0.0603	0.0537	0.2700	12.70%
ICA	33	−0.6643	0.8068	0.4166	0.00%
Max diameter	26	0.1892	0.2131	0.3834	0.00%
Fisher grade	21	0.8167	0.5594	0.1607	5.82%
Hunt-Hess grade	21	0.7816	0.4680	0.1113	1.53%
WFNS grade	9	0.7886	0.5536	0.1973	0.00%
Ruptured status	29	1.8717	4.1266	0.6538	9.77%
Time to treatment	21	−0.1483	0.0941	0.1317	0.00%
PED	36	−0.2663	0.6102	0.6653	21.05%
FD ≥ 2	33	0.9323	1.5471	0.5511	36.65%
FD length	7	0.2377	0.2779	0.4313	0.00%
Aspirin/Clopidogrel	38	0.3711	0.5328	0.4905	25.12%
Adjunctive coiling	35	−0.6537	0.6272	0.3049	0.00%

The rate of good neurological outcome, defined as a modified Rankin Scale (mRS) ≤ 2, at the last follow-up was 84.05% (Supplementary Fig. 20) and did not change on leave-one-out analysis (Supplementary Fig. 21). Good neurological outcome was negatively associated with Hunt-Hess grade (*P* = 0.031). Furthermore, adjunctive coiling was significantly associated with better neurological outcomes (*P* = 0.019), although this may be influenced by case selection bias (Table 5). Multivariate meta-regression results (including age, Hunt-Hess grade, time to treatment, and the percentage of patients who received a PED) showed that none of these variables were significantly associated with a good neurological outcome. There was no significant difference in good neurological outcome rates in the subgroup analysis based on FD type and DAPT regimen. Additionally, 11.77% of patients required post-operative cerebrospinal fluid (CSF) diversion due to hydrocephalus (Supplementary Fig. 22), and 4.94% underwent retreatment of the aneurysm (Supplementary Fig. 23, Table 3).

Among the included studies, four large multicenter trials [16, 22, 30, 38] reported a pooled complete occlusion rate of 87.14%, which is lower than the 87.75% rate found in smaller studies. This difference was not significant (*P* = 0.858) (Supplementary Fig. 24). Funnel plot asymmetry was observed for complete occlusion rates (Supplementary Fig. 25), with Egger’s test (t = 2.86, *P* = 0.007) indicating a significant small-study effect. However, the Begg’s test was not significant (z = 1.44, *P* = 0.149).

Furthermore, large studies demonstrated a lower good neurological outcome rate (81.95%) compared to small studies (86.94%) and the difference was not significant (*P* = 0.420) (Supplementary Fig. 26). Funnel plot asymmetry was also observed for good neurological outcome rates (Supplementary Fig. 27), with significant Begg’s test (z = 3.05, *P* = 0.002) and Egger’s test (t = 7.61, *P* < 0.0001) results.

Baujat plots identified the studies by Ryan et al. [12] and Mokin et al. [16] as the largest contributors to between-study heterogeneity and as having the greatest influence on the pooled estimates of complete occlusion and good neurological outcome rates, respectively. This suggests that these studies may partially explain the observed high I² values (Supplementary Figs. 28, 29).

### Complications

Intra-operative aneurysm rupture occurred in 1.60% of patients (Supplementary Fig. 30). Postoperative complications included hemorrhagic (5.62%) (Supplementary Fig. 31), thromboembolic (6.89%) (Supplementary Fig. 32), and neurologic (6.30%, e.g., paresis) (Supplementary Fig. 33) complications. Additionally, vasospasm was observed in 11.90% of patients, in-stent stenosis in 7.17%, and 6.55% of patients died during follow-up (Table 4) (Supplementary Figs. 34–36). The meta-regression analysis (Table 5 ) showed a significant positive association (*P* = 0.043) between ICA location of aneurysm and rates of intra-operative bleeding and rates of adjunctive coiling had a marginally significant positive correlation (*P* = 0.057) with intra-operative bleeding. Furthermore, post-operative hemorrhage was positively correlated (*P* = 0.023) with Hunt-Hess grade and negatively correlated (*P* = 0.0004) with rates of aneurysms located in ICA. The subgroup analysis of FD type and DAPT regimens showed no significant differences in adverse events. There was no significant difference between small and large studies in complications and mortality rates (Supplementary Figs. 37–41).

### Risk of bias

The majority of the included studies (82.05%) had a low risk of bias (Supplementary Table 3). Among the seven studies with a high risk of bias, a lack of clear demographic and clinical data was the main contributor to the added risk.

## Discussion

### Summary

The optimal management of BBAs remains unclear due to their rarity and limited high-quality evidence. Most literature on BBAs comprises case series and small retrospective studies. Recently, FDs have emerged as a promising treatment, offering parent artery reconstruction and promoting aneurysm occlusion by disrupting blood flow. However, high-quality data on the safety and efficacy of FDs for BBAs is scarce.

This systematic review synthesizes current evidence on FD effectiveness and safety in managing BBAs. Findings suggest FDs achieve high rates of complete aneurysm occlusion and favorable clinical outcomes, with a low incidence of major complications, indicating an acceptable safety profile.

### Functional & radiologic outcome

FDs represent a paradigm shift in aneurysm treatment by focusing on reconstructing the parent artery rather than directly targeting the aneurysm sac. The first FDA-approved FD was the Pipeline Embolization Device (PED), a self-expanding stent with a braided multi-alloy cylindrical mesh, providing higher metal coverage (30–35%) compared to stent-assisted coiling (6–8%) [42]. FDA approval followed two U.S. clinical trials: Pipeline for Intracranial Treatment of Aneurysms (PITA) and Pipeline for Uncoilable or Failed Aneurysms (PUFS) [43, 44]. Initially used for large, complex unruptured proximal carotid aneurysms, recent studies suggest FDs are a safe, effective alternative to microsurgery for ruptured aneurysms, showing acceptable occlusion and morbidity rates [45–47].

Our study found a high rate of complete occlusion (87.56%) at 12.72 months post-FD placement, with 84.05% of patients achieving good neurological outcomes. The study’s findings align with two previous reviews on BBAs treatment modalities. Sanchez et al. noted an initial occlusion rate of 32.3% that improved to 89.1% upon follow-up [48], similar to the current study’s 87.56% complete occlusion rate at the last follow-up. Immediate complete occlusion with FDs is rare, but their ability to reduce re-rupture rates offers significant protection against bleeding even before full closure. FDs divert blood flow and act as scaffolds for neointimal proliferation, leading to complete occlusion only after neo-endothelialization, with the timeline varying by aneurysm morphology. Peschillo et al. reviewed various BBA treatments, reporting 82% good clinical outcomes with FDs alone [49]. In our study, 30.10% of received adjunctive coiling during FD placement, potentially explaining better functional outcomes. Meta-regression analysis indicated a significant association between adjunctive coiling and higher rates of good functional outcomes. However, in multivariate meta-regression controlling for age, disease severity, and device type, this association was no longer significant, suggesting that the initial finding was likely driven by case selection.

Our study identified younger age had a marginally significant association with complete occlusion rates. Younger patients typically have a greater physiological reserve, enabling them to better withstand the stress of a hemorrhagic stroke and subsequent endovascular interventions [50, 51]. They are also less likely to suffer from significant atherosclerosis, which can complicate device placement and elevate the risk of procedure-related complications. Additionally, younger patients generally have better overall health and vascular response, which contributes to a more efficient endothelialization process [12, 52]. Our study showed that higher Hunt-Hess grade was associated with worse neurological outcome. The Hunt–Hess scale reflects the initial clinical severity of aneurysmal subarachnoid hemorrhage (level of consciousness and focal deficits), a higher Hunt-Hess grade generally indicates more extensive early brain injury and physiologic stress. Additionally, a prognostic study in poor-grade aneurysmal subarachnoid hemorrhage population reported that worse Hunt-Hess grade was independently associated with poor long-term functional outcome [53]. Adjunctive coiling offers immediate aneurysm occlusion, reducing the risk of early rebleeding and promoting thrombosis and endothelialization over the aneurysm neck [54]. Coils provide scaffolding for the FD, enhance intra-aneurysmal flow patterns, and stabilize the FD, reducing risks of stent migration or kinking [55]. It is important to note that the decision to use adjunctive coiling with flow diversion should be individualized and should take into account factors such as the aneurysm’s morphology, location, and the individual’s clinical presentation.

Additionally, our review found no link between the number of stents deployed and outcomes or complications post-FD placement. In contrast, Zhu et al. reported better outcomes with multiple FDs [56], possibly due to selection bias where patients with worse initial conditions received multiple FDs, leading to improved outcomes.

### Complications

FDs present a promising treatment for BBAs but come with significant complications, particularly when used for ruptured aneurysms. These complications stem from the intraluminal stent’s risk of thromboembolic events and the necessity of DAPT to mitigate this risk.

Immediate procedural complications include intra-operative bleeding. This is because BBAs are fragile, and manipulating the microcatheters and microwires near them can cause them to rupture, further leading to subarachnoid hemorrhage or intracerebral hemorrhage [57]. Our study demonstrated that 1.60% of patients with BBAs experienced intra-operative bleeding, which was consistent with the rates reported in the review by Zhu et al. (1.21%) on 165 patients undergoing FD for BBAs [56]. Thromboembolic events are another concern. FD deployment can damage the endothelium of the blood vessel, increasing the risk of thrombus formation and potentially causing ischemic strokes. This complication was observed in 6.89% of patients included in this review. While Sanchez et al. reported a lower rate (4.3%) of thromboembolic events following FD placement, Zhu et al. reported a higher rate of 12% for perioperative ischemic events [48, 56].

Delayed complications mainly stem from the DAPT used to prevent thromboembolic complications after FD placement. DAPT increases the risk of bleeding, especially for patients with SAH who may require additional procedures like cerebrospinal fluid diversion or decompressive craniectomy [58]. Our review found that 11.77% of patients required CSF diversion and 5.62% experienced postoperative hemorrhage. While some bleeding might be due to the aneurysm re-bleeding, most are likely linked to the additional surgeries following FD placement.

Furthermore, meta-regression analysis revealed no significant association between the use of clopidogrel (compared with other P2Y12 inhibitors, including ticagrelor and prasugrel) and the outcomes of FDs in BBAs. These findings align with previous meta-analyses, which demonstrated no significant differences in complication rates or functional outcomes between patients receiving ticagrelor and those receiving clopidogrel after endovascular treatment of cerebral aneurysms [59, 60]. Accordingly, prior studies have recommended clopidogrel as the first-line antiplatelet agent, reserving ticagrelor and prasugrel for patients who are non-responders to clopidogrel [61, 62]. However, owing to the limited number of studies evaluating ticagrelor and prasugrel in the context of BBAs, our results remain inconclusive for guiding the choice of antiplatelet regimen in these cases. Further research is needed to compare the efficacy and safety of these treatment options in this specific population.

The majority of FDs utilized in the included studies were PED (76.08%), followed by FRED (9.79%). While the PED is a single-layer braided flow-diverting stent, FRED consists of a dual-layer stent with an outer flow-diverting layer and an inner scaffold layer [63]. The meta-regression presented here did not reveal any major significant differences between FD types, which may have been due to confounding factors and the limited number of patients who underwent FRED, comparative studies are required to establish a robust recommendation for BBAs.

Novel FDs with surface-modified technology have been introduced to reduce thromboembolic risks without needing DAPT [64]. The single anti-platelet therapy utilized in the setting of surface-modified FDs is assumed to lower the risk of hemorrhagic complications. Our review found two study using a surface-modified FD (PED-Shield), which demonstrated that single antiplatelet therapy effectively prevented thromboembolic events and reduced re-bleeding risks [33, 37]. Moreover, a recent multi-institutional study on the safety and efficacy of PED-Shield for the treatment of pseudoaneurysms (including dissecting, blister, and iatrogenic pseudoaneurysms) was published [65]. In the latter series, 12.5% of patients received single anti-platelet therapy and none of the patients experienced thromboembolic events, highlighting the potential for earlier cessation of DAPT in the setting of surface-modified FDs. However, due to the limited studies on surface-modified FDs, we couldn’t perform a subgroup analysis. Future comparative studies are necessary to evaluate the safety and efficacy of this promising technology.

### Comparison

Untreated BBAs are highly aggressive, often leading to repeated ruptures, severe complications, and high mortality rates [66, 67], with a one-year mortality rate of about 65% [68]. Surgical treatments like clipping, wrapping, and bypass have been used for BBAs, achieving high immediate occlusion rates but also posing significant risks such as intraoperative rupture, parent artery stenosis, and poor neurological outcomes [69]. A systematic review reported a 90.7% early occlusion rate for surgical treatments, but with notable risks of re-hemorrhage (17.5%), vasospasm (15.8%), stroke (10.1%), and a 7.4% mortality rate [70]. Another study indicated a high rate of intra-operative complications, including rupture (28.8%), vasospasm (21.6%), hydrocephalus (9.9%), infarction (6.3%), and a 23.1% rate of poor neurological outcomes [66]. In comparison, Although FDs may have lower immediate complete occlusion rates, according to our findings, they show comparable occlusion rates (93.55%) during follow-ups with significantly lower rates of poor functional outcomes (mRS > 2, 11.37%) and mortality (2.61%). These findings suggest that FDs offer a safer and more effective treatment for BBAs compared to traditional microsurgery, with improved long-term outcomes and fewer complications.

Stent-assisted coiling (SAC), particularly when multiple stents are used, has demonstrated high immediate occlusion rates and favorable clinical outcomes in patients with BBAs. For example, a study comparing double low-profile visualized intraluminal support (LVIS) stents with a single PED reported that double LVIS achieved a 46.9% complete occlusion rate, a 44.2% favorable clinical outcome rate, and low complication rates, with results comparable to those of PEDs [38]. Another study evaluating SAC for BBAs found complete obliteration rates of 42.9%, 78.4%, and 88.2% in patients treated with one, two, and three or more stents, respectively. The corresponding recurrence rates were 38.1% for single-stent cases, 13.5% for two-stent cases, and 5.9% for patients who received three or more stents [71]. Collectively, these findings suggest that SAC is a safe and effective treatment option for BBAs; however, it is associated with higher recurrence rates compared with flow diversion and may require multiple stents to achieve similar efficacy. FDs could be used for the treatment of recurrent aneurysms after SAC [72].

### Limitations

Our study has several limitations. The evidence is weakened by the heterogeneity between included studies and the rarity of the disease. Due to the rarity of the disease and complications following FD placement, meta-regression was limited, missing some correlated factors. Many of the included studies did not provide a clear or standardized definition of BBAs. Variations in occlusion criteria restricted our analysis to complete occlusion. Short follow-up periods may have affected observed clinical safety and efficacy. The time between symptom onset and treatment varied significantly, influencing occlusion and complication rates. Most studies were small and monocentric, potentially causing a small study effect. Some studies had overlapping patients but were included due to incomplete overlap and additional data provided for these patients.

## Conclusion

This systematic review highlights the safety and efficacy of FDs for treating BBAs in carefully selected patients. The promising rates of complete aneurysm occlusion and favorable neurological outcomes indicate that FD treatment offers significant benefits for patients. The majority of treatments successfully utilized PEDs. Key factors influencing treatment success include patient age and disease severity, emphasizing the importance of careful patient selection and management. Complications were rare and manageable, and the type of FD and DAPT did not significantly impact outcomes. These findings support the continued use and optimization of FD treatment strategies for BBAs.

## Supplementary Information

Below is the link to the electronic supplementary material.

Supplementary Material 1 (DOCX 6.53 MB)

## Data Availability

Data is available upon reasonable request from the corresponding author.
