# Supplementary material for: Safety and efficacy of flow diversion for blood blister aneurysms: A comprehensive systematic review and meta-analysis
Source: Neurosurg Rev. 2026 Apr 25;49(1):375. doi: 10.1007/s10143-026-04278-x (PMC13109133; doi:10.1007/s10143-026-04278-x)
Supplement: Supplementary file 1 — Supplementary Material 1 (DOCX 6.53 MB) [file 10143_2026_4278_MOESM1_ESM.docx]

| **Supplementary Table 1.** Keywords used for search in various database. | |
| --- | --- |
| 1 | (Aneurysm OR Aneurysms) |
| 2 | (Blister OR blood-blister OR blood blister-like) |
| 1 AND 2 | |

| **Supplementary Table 2.** Characteristics of included studies reporting inclusion criteria for BBAs. | |
| --- | --- |
| **Author, year** | **Inclusion Criteria for BBAs** |
| Çinar, 2013 [1] | Ruptured blister-like aneurysms |
| Chalouhi, 2014 [2] | Blister-like aneurysms defined as a shallow, broadbased aneurysm arising from non-branching sites of the supraclinoid ICA or the basilar artery. |
| Yoon, 2014 [3] | Blister aneurysms that were ruptured, small, wide necked, and thin walled, arising at nonbranching sites in the distal ICA. |
| Aydin, 2015 [4] | Ruptured blood-blister aneurysms defined as aneurysms in non-branching sites of supraclinoid internal carotid or basilar arteries with a typical blister-like shape. |
| Lin, 2015 [5] | Ruptured blister-like aneurysms |
| Cerejo, 2017 [6] | Blood blister-like aneurysms |
| Linfante, 2017 [7] | Ruptured blister-type aneurysms defined as very small (1–2 mm) irregularly-shaped aneurysms with a wide or non-discernable neck. |
| Luecking, 2017 [8] | Blister-like aneurysms |
| Ryan, 2017 [9] | Ruptured blister aneurysms were defined as small lesions occurring on the sidewall of the ICA at nonbranching sites, and having dome width equal to or greater than its height. |
| Yang, 2017 [10] | Ruptured blood blister aneurysms with characteristic small- and wide-necked configuration, arising from non-branching sites of the distal ICA. |
| Kumar, 2017 [11] | Ruptured blister-like aneurysms at the nonbranching sites of intracranial circulation. |
| Hellstern, 2018 [12] | Ruptured blood blister aneurysms defined as aneurysms with a diameter of less than or equal to 2 mm. |
| Lozupone, 2018 [13] | Ruptured Blood blister-like aneurysms |
| Mokin, 2018 [14] | Blood blister aneurysms located at non-branching segments along the anteromedial wall of the supraclinoidal ICA. |
| Parthasarathy, 2018 [15] | Ruptured blister-like aneurysms |
| Ghorbani, 2019 [16] | Ruptured blood blister aneurysms. |
| Griffin, 2019 [17] | Blister aneurysms |
| Capocci, 2020 [18] | Ruptured blister-like aneurysms |
| Incandela, 2020 [19] | Ruptured blood blister aneurysms |
| Möhlenbruch, 2020 [20] | Blister-like aneurysms were defined as small, conical, wide-neck aneurysms located at a nonbranching site of the circle of Willis. |
| Zhang, 2020 [21] | Ruptured blood blister-like aneurysms defined as small (<5 mm), shallow, broad-based aneurysms originating from unbranched sites on the dorsal wall of the supraclinoid ICA. |
| Aboukais, 2021 [22] | Ruptured blood blister-like aneurysms defined as small non-branching aneurysm  located on supraclinoid segment of ICA. |
| Gopinathan, 2021 [23] | Ruptured blood blister/blister-like aneurysms |
| Tanburoglu, 2021 [24] | Ruptured blood blister-like aneurysms located at non-branching ICA |
| Wang, 2021 [25] | 1) Typical clinical presentation of SAH. 2) Computed tomography (CT) confirmed SAH. 3) The aneurysm located in nonbranching site of supra-clinoidal segment ICA on digital subtraction angiography (DSA) imaging. 4) Accompanied with stenosis or dilation of parent artery on DSA. 5) Aneurysmal shape changes obvious on 2 different angiograms in a short time. 6) Cerebral vasospasm was obvious in pre- or intraoperation imaging. 7) Poor contrast filling in aneurysmal sac.  Criteria 1 to 3 were constant while criteria 4 to 7 must be meet  at least once. |
| Zhang, 2021 [26] | Blood blister-like aneurysms of ICA |
| Zhong, 2021 [27] | Ruptured blister aneurysms |
| Alpay, 2022 [28] | Ruptured blister-like aneurysms |
| Liu, 2022 [29] | Ruptured blood blister aneurysms of the ICA |
| Feng, 2023 [30] | Ruptured blood blister aneurysms |
| Madjidyar, 2023 [31] | Ruptured blood blister aneurysms |
| Reidy, 2023 [32] | Blister aneurysms defined as aneurysms arising from the supraclinoid segment of ICA above the ophthalmic artery origin that were lateral or superiorly projecting, or small broad-based aneurysms not at typical arterial branching sites. |
| Zhang, 2023 [33] | ICA blood blister-like aneurysm was diagnosed based on the following criteria: (1) the presence of a small non-branching aneurysm in the supraclinoid segment of ICA, which is responsible for subarachnoid hemorrhage (SAH) and absence of other associated intracranial aneurysms; (2) the presence of small aneurysms (typically less than 10 mm in size) without a discernible neck; (3) the observation of thin-walled and rapid growth in computer tomographic angiography (CTA), magnetic-resonance angiography (MRA), or digital subtraction angiography (DSA) |
| Hoffman, 2024 [34] | Ruptured blister aneurysm |
| Chen, 2025 [35] | The diagnostic criteria for blood blister-like aneurysms were as follows: (1) ruptured aneurysm located at the anterior wall or the anterior medial wall of the nonbranching sites of the supraclinoid ICA, (2) typical appearance as a small hemispherical bulge (typically less than 10 mm in size) or an irregular bleb-like protrusion without a discernible neck, (3) newly developing or rapidly growing sac (< 2 weeks) on repeated  angiograms. |
| Chen, 2025 [36] | Ruptured blood blister-like aneurysms located at the nonbranching site of the dorsal  ICA wall. |
| Chen, 2025 [37] | Blood blister-like aneurysms identified as aneurysms arising from the non-branching side (dorsal wall) of the supraclinoid ICA |
| Dange, 2025 [38] | Ruptured blister aneurysms defined as small, hemispherical bulges along non-branching vessel segments, typically <3 mm, poorly demarcated, and without a classical neck. |
| Wroe, 2025 [39] | As the terms blister and blood blisterlike aneurysm are often used in the literature interchangeably with dissecting aneurysm, and no well-defined distinction exists, all these aneurysms were grouped and labeled as dissecting. |

| **Supplementary Table 3**. Risk of bias assessment using JBI Critical Appraisal Checklist for Case Series. | | | | | | | | | | | |
| --- | --- | --- | --- | --- | --- | --- | --- | --- | --- | --- | --- |
| **Author, year** | **Q1** | **Q2** | **Q3** | **Q4** | **Q5** | **Q6** | **Q7** | **Q8** | **Q9** | **Q10** | **Overall** |
| Çinar, 2013 [1] | Y | Y | Y | Y | Y | Y | Y | Y | Y | Y | Low |
| Chalouhi, 2014 [2] | Y | Y | Y | Y | Y | Y | Y | Y | Y | Y | Low |
| Yoon, 2014 [3] | Y | Y | Y | Y | Y | Y | Y | Y | Y | Y | Low |
| Aydin, 2015 [4] | Y | Y | Y | Y | Y | Y | Y | Y | Y | Y | Low |
| Lin, 2015 [5] | Y | Y | Y | Y | Y | N | Y | Y | Y | Y | High |
| Cerejo, 2017 [6] | Y | Y | Y | Y | Y | Y | Y | Y | Y | Y | Low |
| Linfante, 2017 [7] | Y | Y | Y | Y | Y | Y | Y | Y | Y | Y | Low |
| Luecking, 2017 [8] | Y | Y | Y | Y | Y | N | N | Y | Y | Y | High |
| Ryan, 2017 [9] | Y | Y | Y | Y | Y | Y | Y | Y | Y | Y | Low |
| Yang, 2017 [10] | Y | Y | Y | Y | Y | Y | Y | Y | Y | Y | Low |
| Kumar, 2017 [11] | Y | Y | Y | Y | Y | Y | Y | Y | Y | Y | Low |
| Hellstern, 2018 [12] | Y | Y | Y | Y | Y | Y | Y | Y | Y | Y | Low |
| Lozupone, 2018 [13] | Y | Y | Y | Y | Y | Y | Y | Y | Y | Y | Low |
| Mokin, 2018 [14] | Y | Y | Y | Y | Y | Y | Y | Y | Y | Y | Low |
| Parthasarathy, 2018 [15] | Y | Y | Y | Y | Y | Y | Y | Y | Y | Y | Low |
| Ghorbani, 2019 [16] | Y | Y | Y | Y | Y | Y | Y | Y | Y | Y | Low |
| Griffin, 2019 [17] | Y | Y | Y | Y | Y | N | N | Y | Y | Y | High |
| Capocci, 2020 [18] | Y | Y | Y | Y | Y | Y | Y | Y | Y | Y | Low |
| Incandela, 2020 [19] | Y | Y | Y | Y | Y | Y | Y | Y | Y | Y | Low |
| Möhlenbruch, 2020 [20] | Y | Y | Y | Y | Y | Y | Y | Y | Y | Y | Low |
| Zhang, 2020 [21] | Y | Y | Y | Y | Y | Y | Y | Y | Y | Y | Low |
| Aboukais, 2021 [22] | Y | Y | Y | Y | Y | Y | Y | Y | Y | Y | Low |
| Gopinathan, 2021 [23] | Y | Y | Y | Y | Y | Y | Y | Y | Y | Y | Low |
| Tanburoglu, 2021 [24] | Y | Y | Y | Y | Y | Y | Y | Y | Y | Y | Low |
| Wang, 2021 [25] | Y | Y | Y | Y | Y | Y | Y | Y | Y | Y | Low |
| Zhang, 2021 [26] | Y | Y | Y | Y | Y | Y | Y | Y | Y | Y | Low |
| Zhong, 2021 [27] | Y | Y | Y | Y | Y | N | N | N | Y | Y | High |
| Alpay, 2022 [28] | Y | Y | Y | Y | Y | N | N | U | Y | Y | High |
| Liu, 2022 [29] | Y | Y | Y | Y | Y | Y | Y | Y | Y | Y | Low |
| Feng, 2023 [30] | Y | Y | Y | Y | Y | Y | Y | Y | Y | Y | Low |
| Madjidyar, 2023 [31] | Y | Y | Y | Y | Y | Y | Y | Y | Y | Y | Low |
| Reidy, 2023 [32] | Y | Y | Y | Y | Y | Y | Y | Y | Y | Y | Low |
| Zhang, 2023 [33] | Y | Y | Y | Y | Y | Y | Y | Y | Y | Y | Low |
| Hoffman, 2024 [34] | Y | Y | Y | Y | Y | Y | Y | Y | Y | Y | Low |
| Chen, 2025 [35] | Y | Y | Y | Y | Y | Y | Y | Y | Y | Y | Low |
| Chen, 2025 [36] | Y | Y | Y | Y | Y | Y | Y | Y | Y | Y | Low |
| Chen, 2025 [37] | Y | Y | Y | Y | Y | N | Y | Y | Y | Y | High |
| Dange, 2025 [38] | Y | Y | Y | Y | Y | Y | Y | Y | Y | Y | Low |
| Wroe, 2025 [39] | N | Y | Y | Y | Y | Y | Y | Y | Y | Y | High |
| Abbreviations: Y: Yes; N: No; U: Unclear.  Questions: Q1: Were there clear criteria for inclusion in the case series? Q2: Was the condition measured in a standard, reliable way for all participants included in the case series?  Q3: Were valid methods used for identification of the condition for all participants included in the case series?  Q4: Did the case series have consecutive inclusion of participants?  Q5: Did the case series have complete inclusion of participants?  Q6: Was there clear reporting of the demographics of the participants in the study?  Q7: Was there clear reporting of clinical information of the participants?  Q8: Were the outcomes or follow up results of cases clearly reported?  Q9: Was there clear reporting of the presenting site(s)/clinic(s) demographic information?  Q10: Was statistical analysis appropriate? | | | | | | | | | | | |

**Supplementary Figure 1**. Forest plot demonstrating the mean age of participants.
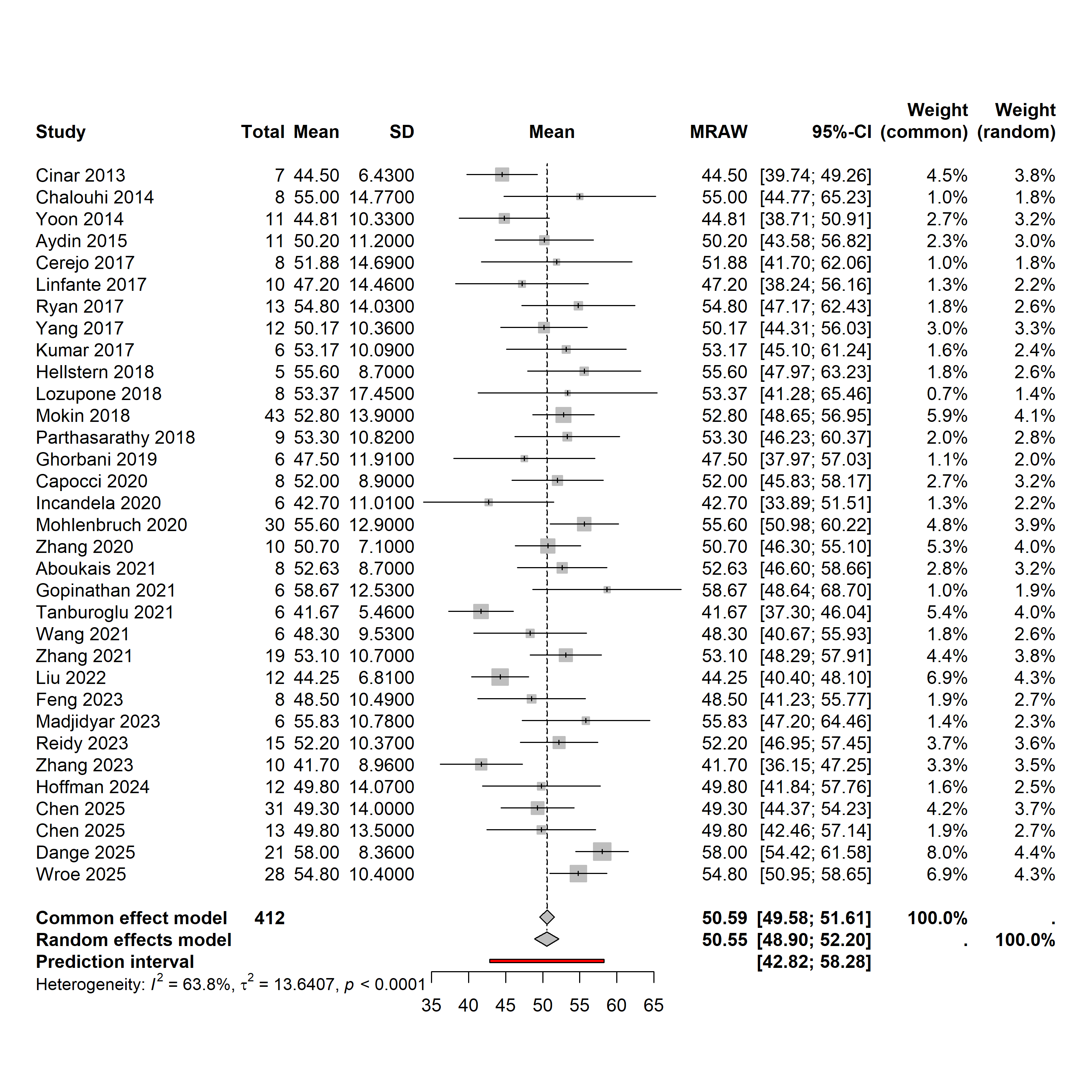


**Supplementary Figure 2.** Forest plot demonstrating proportion of female participants.
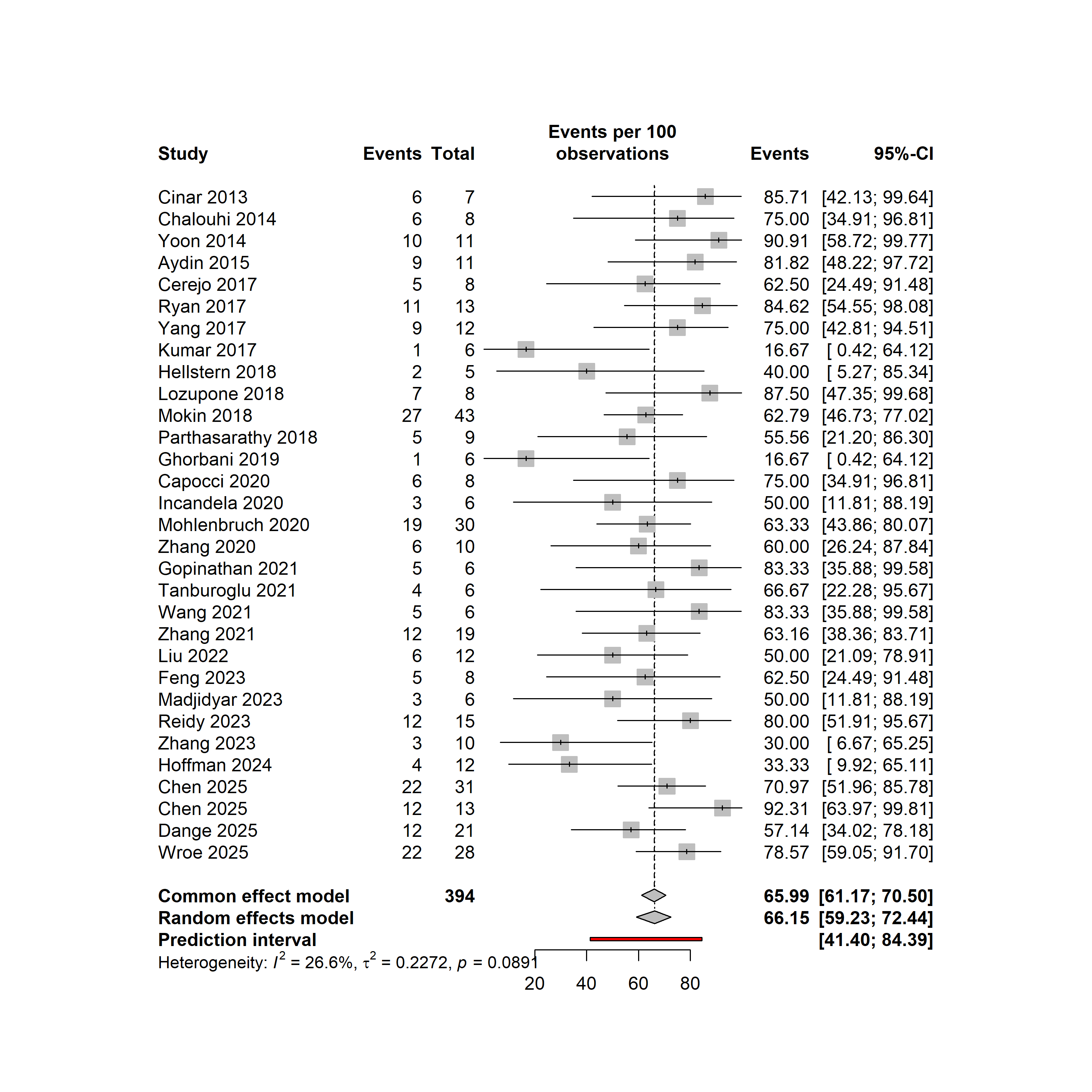


**Supplementary Figure 3.** Forest plot demonstrating proportion of aneurysms located in ICA.
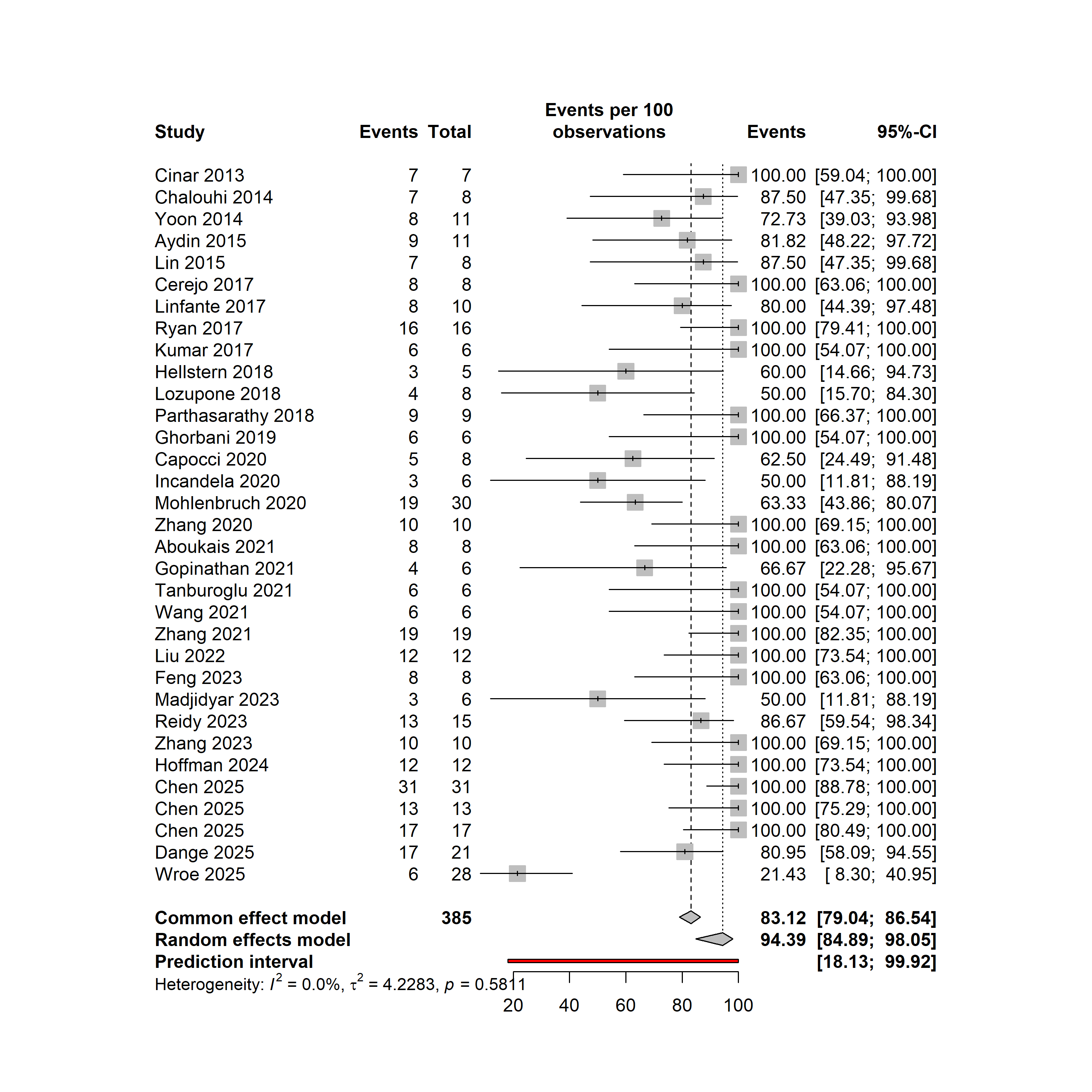


**Supplementary Figure 4.** Forest plot demonstrating the proportion of ruptured aneurysms at presentation.
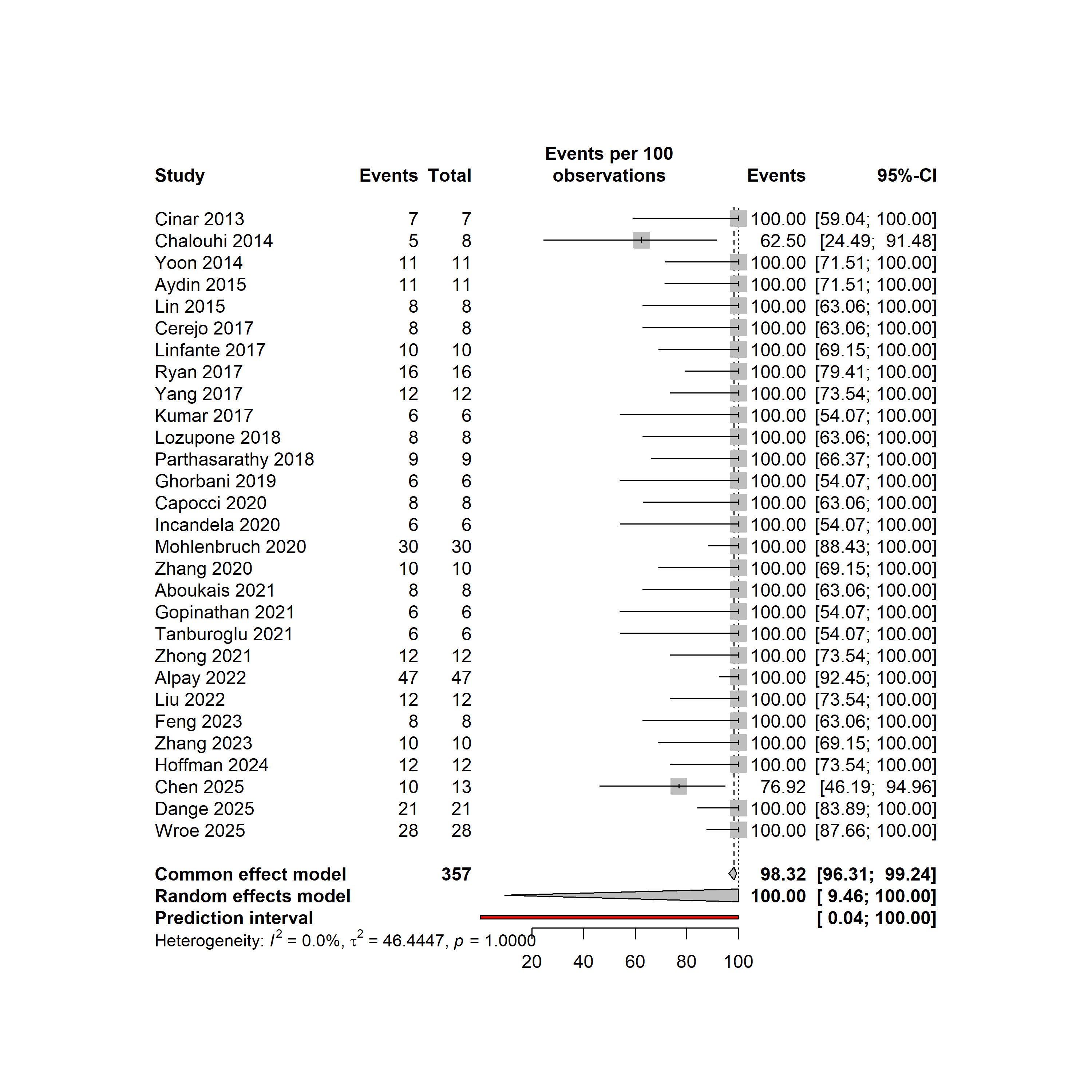


**Supplementary Figure 5.** Forest plot demonstrating the mean maximum aneurysm diameter.
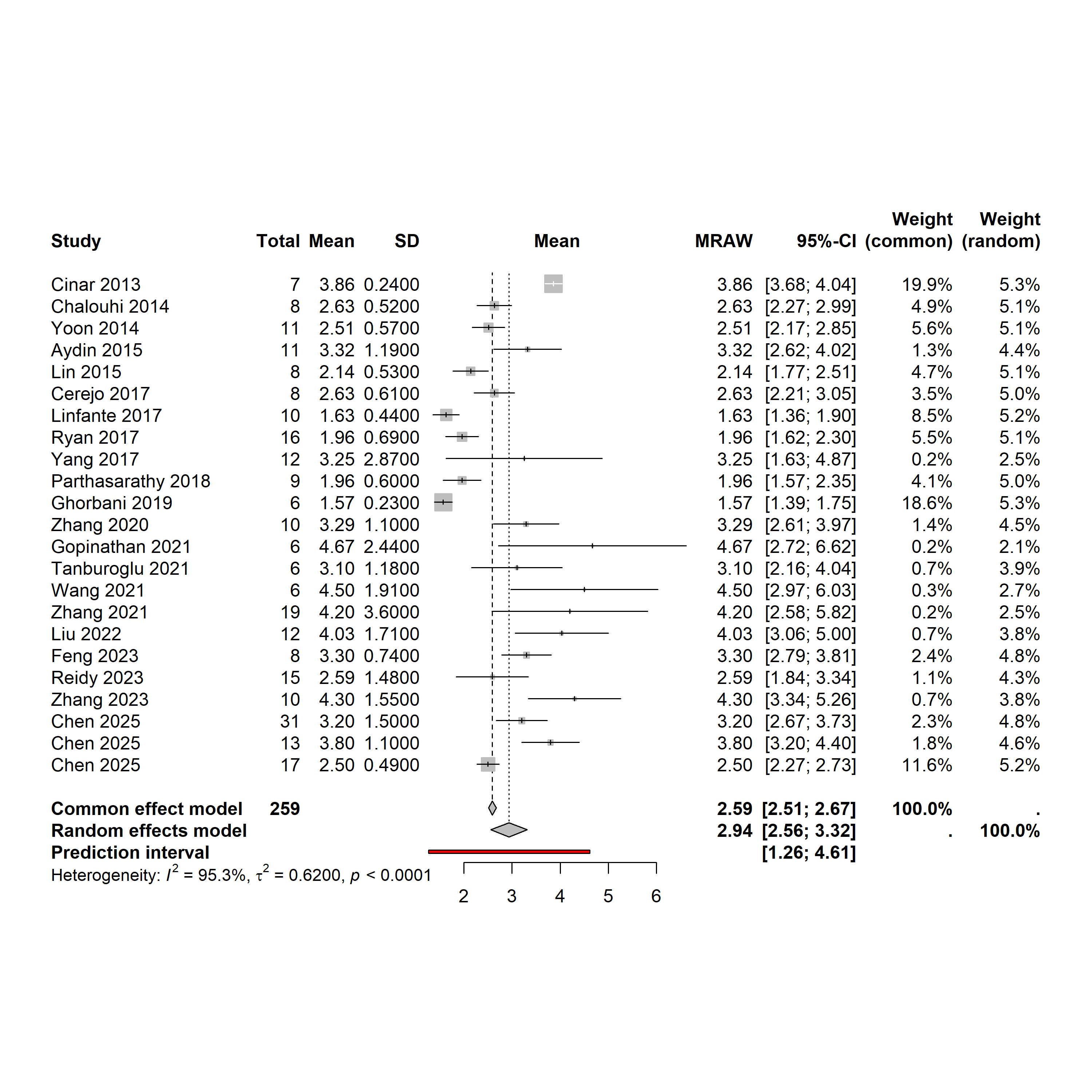


**Supplementary Figure 6.** Forest plot demonstrating the mean Fisher's grade of aneurysms.
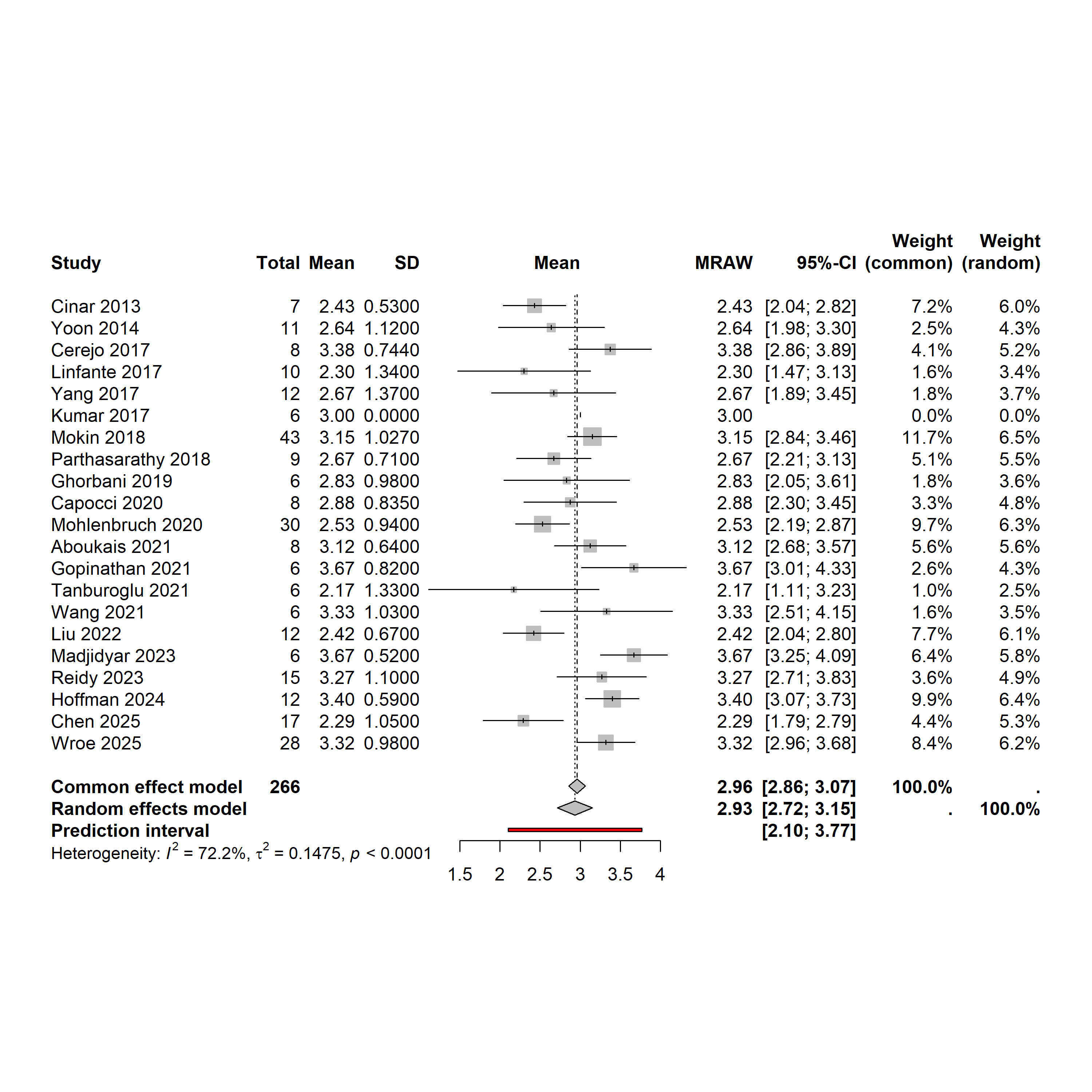


**Supplementary Figure 7.** Forest plot demonstrating the mean Hunt and Hess grade of aneurysms.
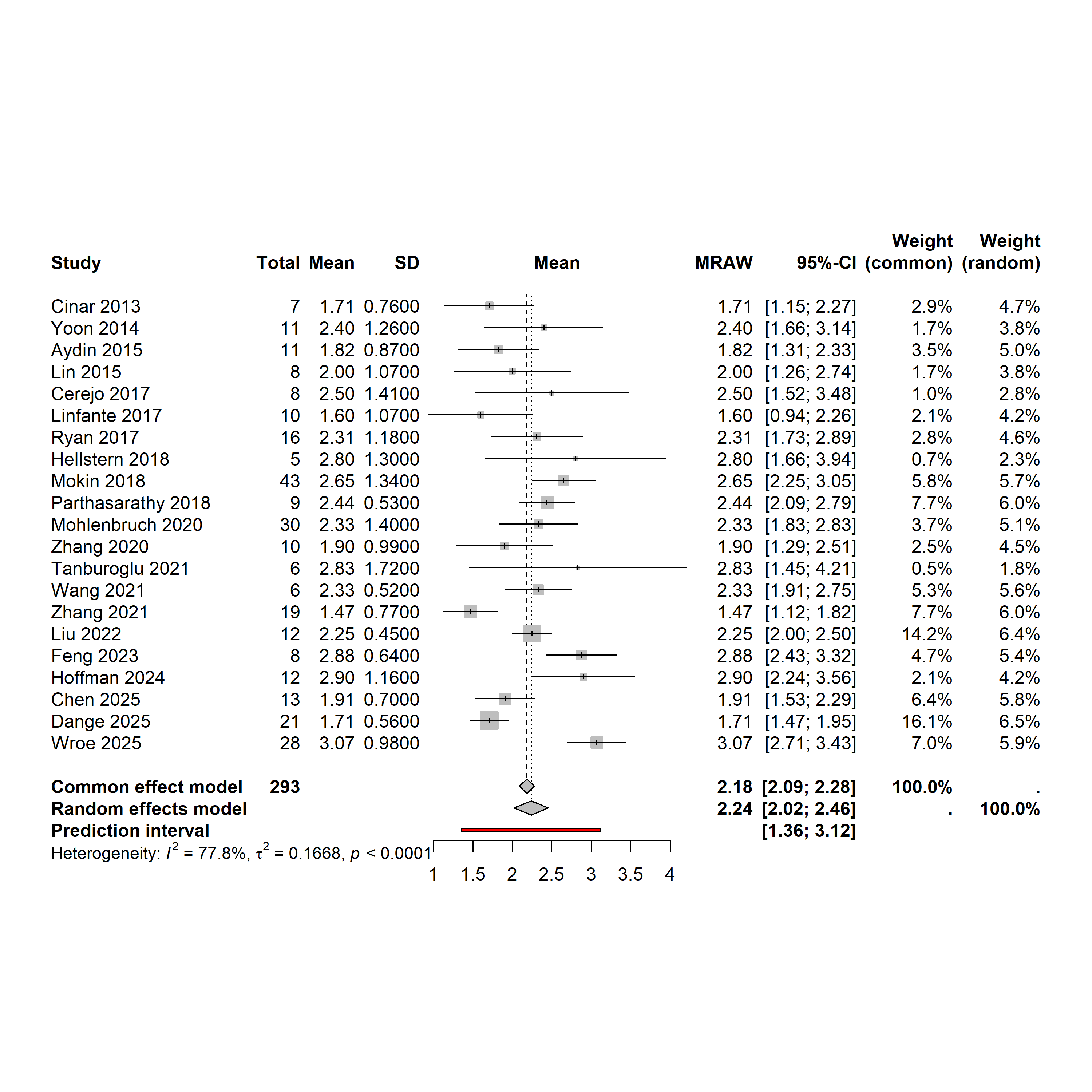


**Supplementary Figure 8.** Forest plot demonstrating the mean score of World Federation of Neurological Surgeons (WFNS) scale.
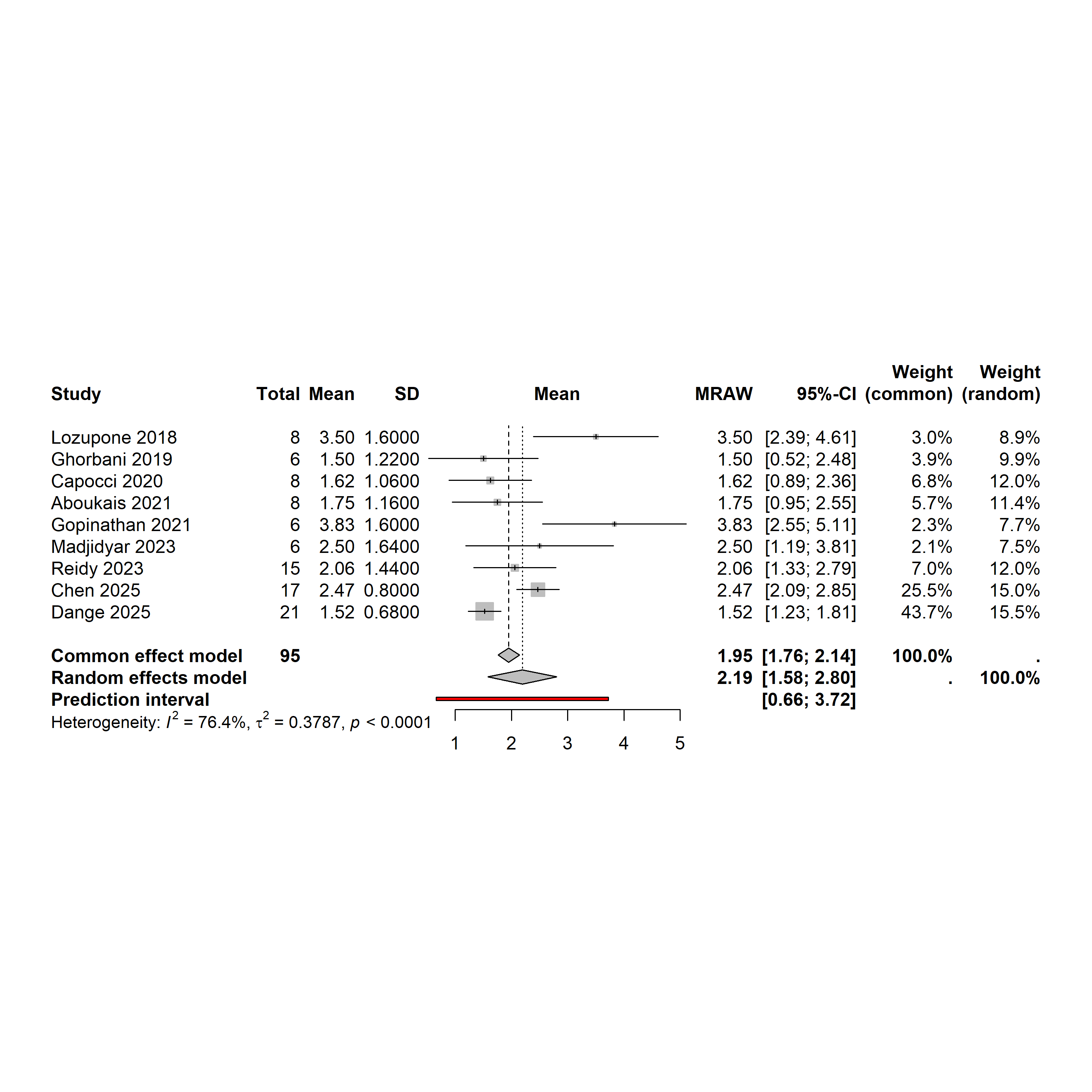


**Supplementary Figure 9.** Forest plot demonstrating the mean time to treatment in days.
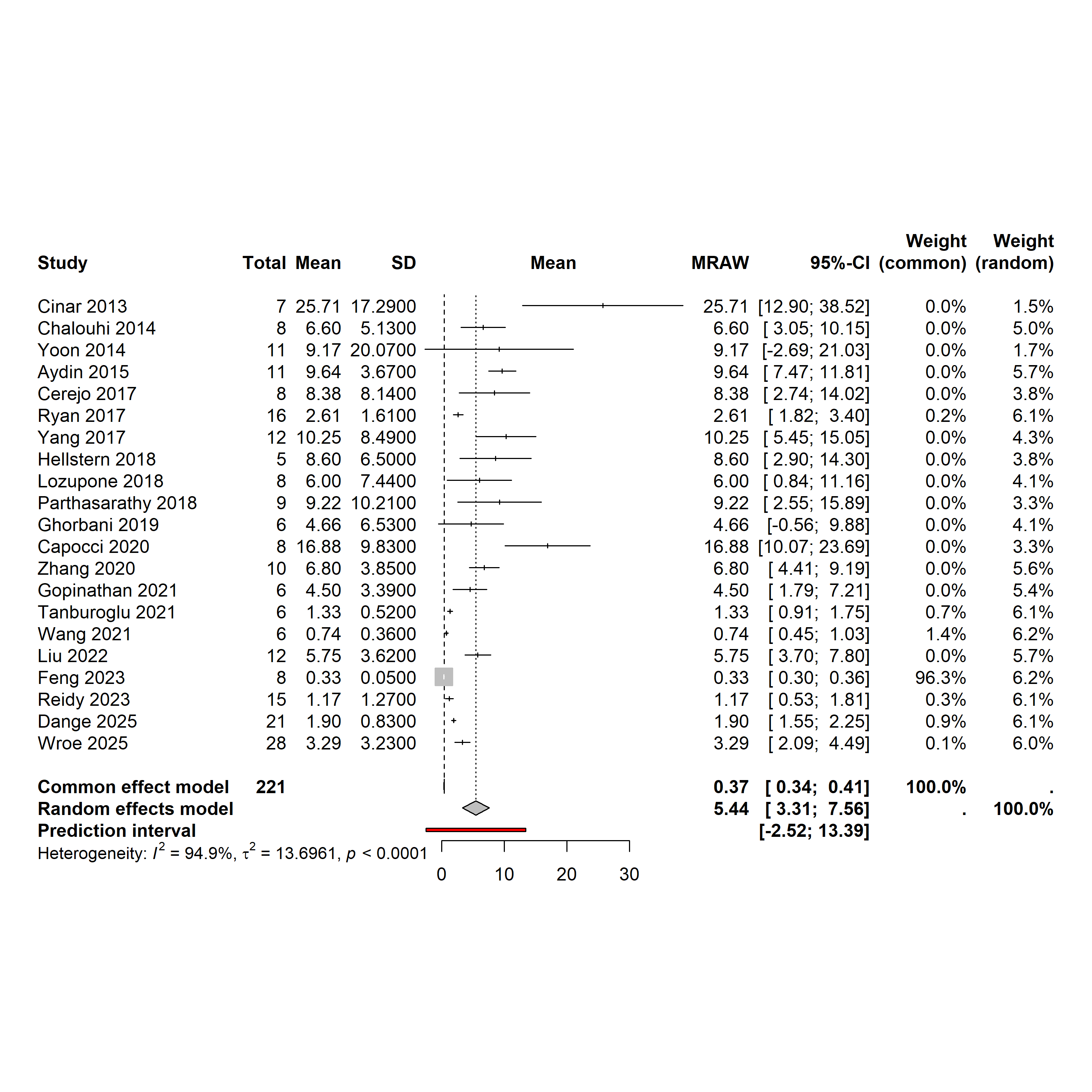


**Supplementary Figure 10**. Forest plot demonstrating the proportion of patients with Pipeline Embolization Device (PED) employment.
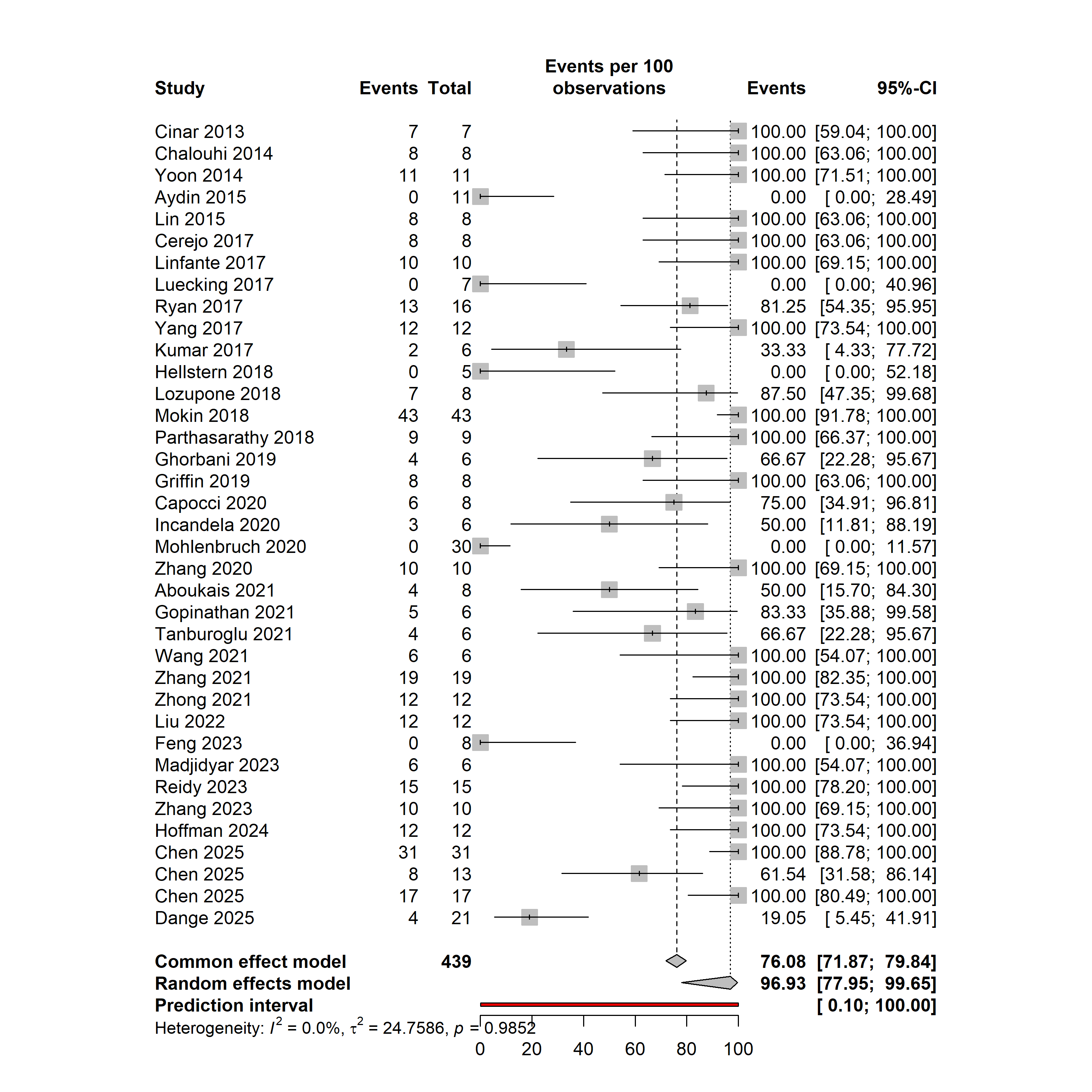


**Supplementary Figure 11.** Forest plot demonstrating the mean flow diverter length.
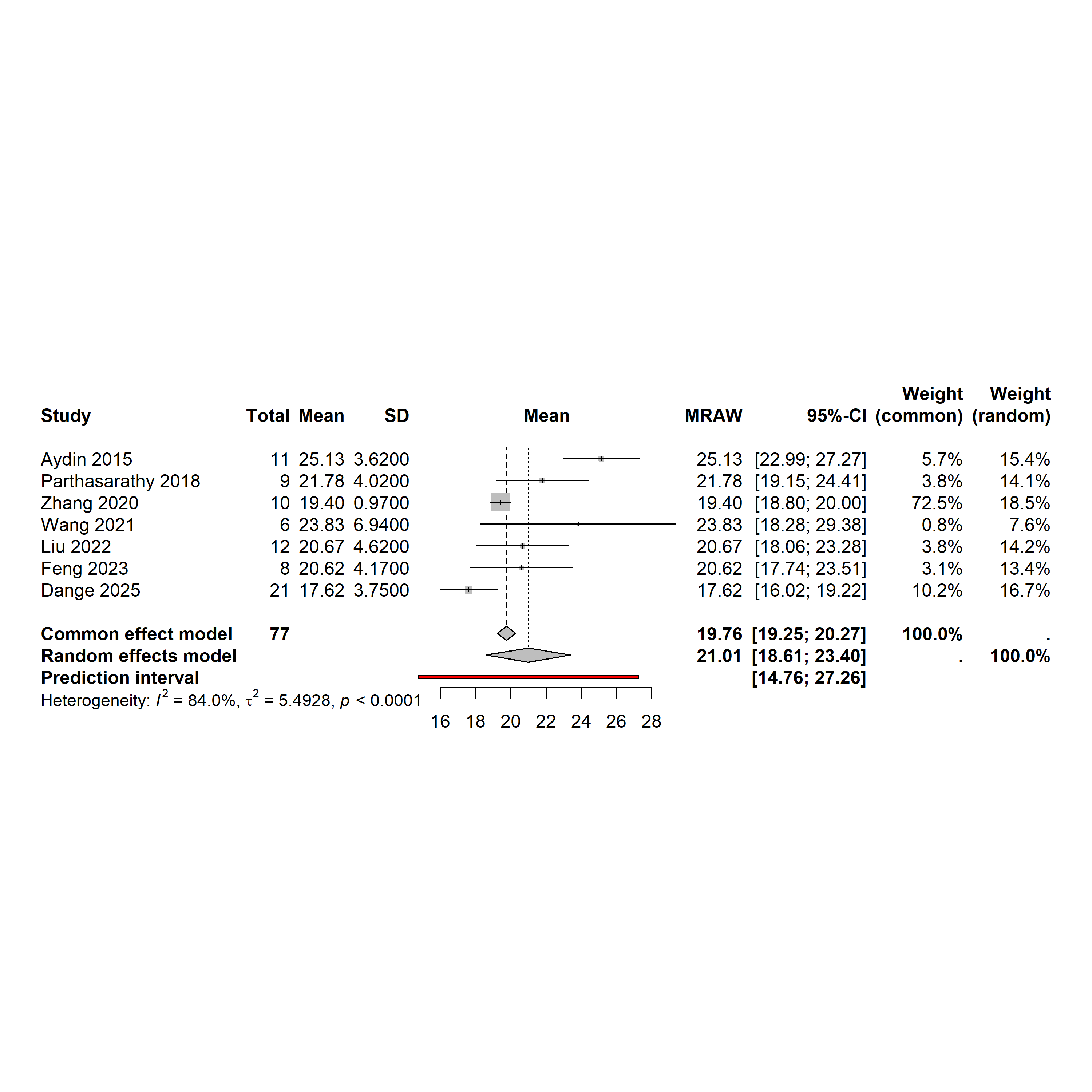


**Supplementary Figure 12.** Forest plot demonstrating proportion of patients with two or more flow diverter employments.
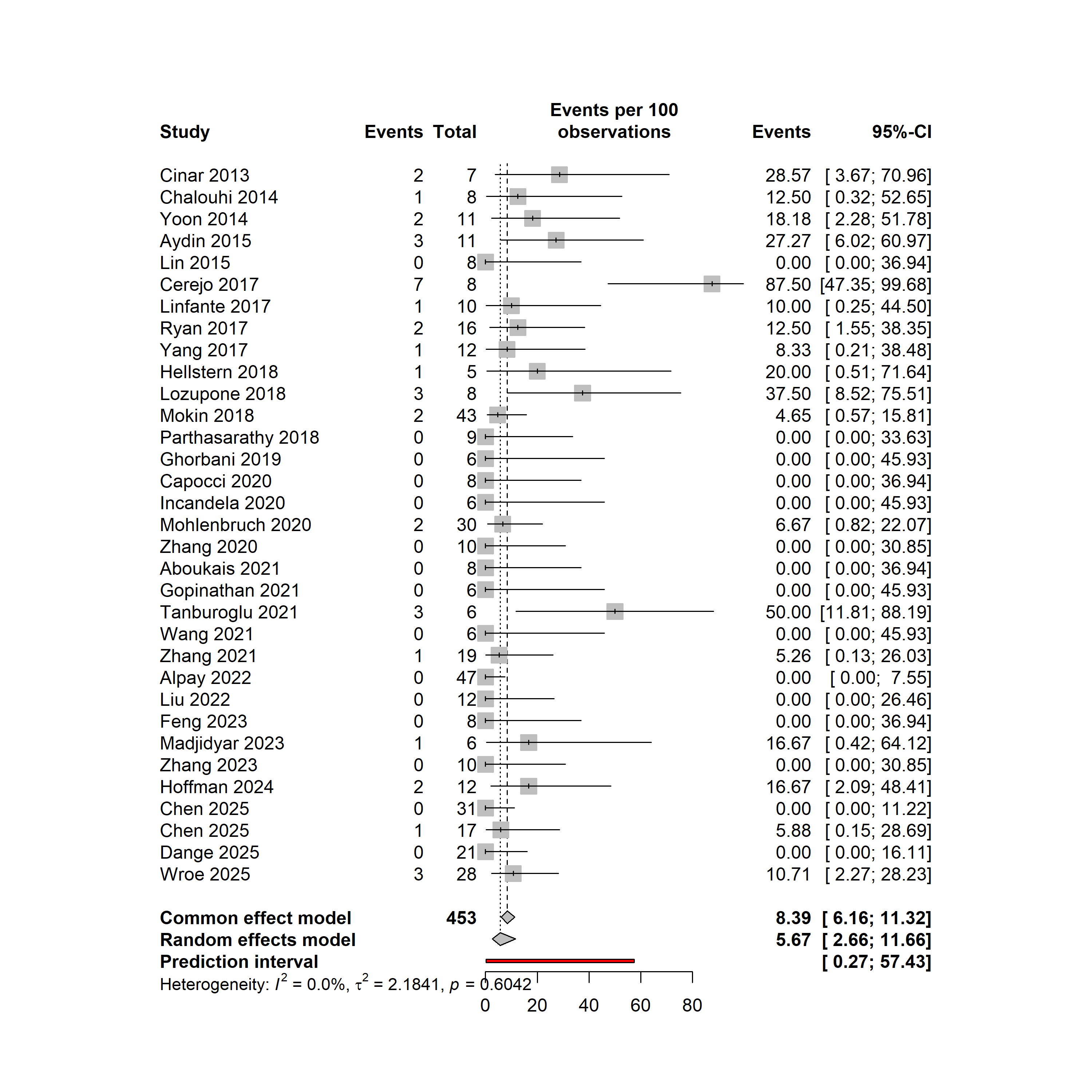


**Supplementary Figure 13.** Forest plot demonstrating proportion of patients undergoing adjunctive coiling.
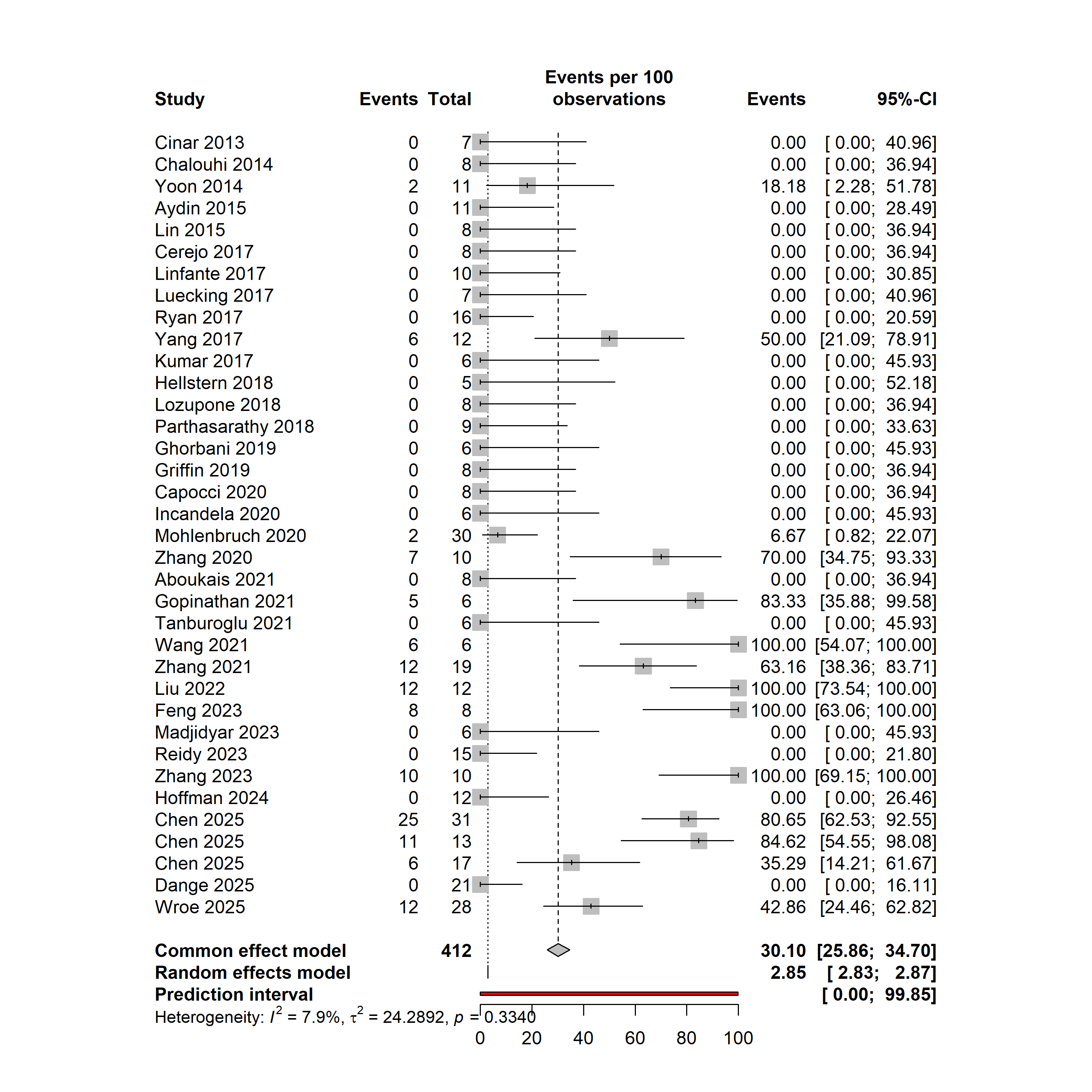


**Supplementary Figure 14.** Forest plot demonstrating proportion of patients using Aspirin/ Clopidogrel regimen following treatment.
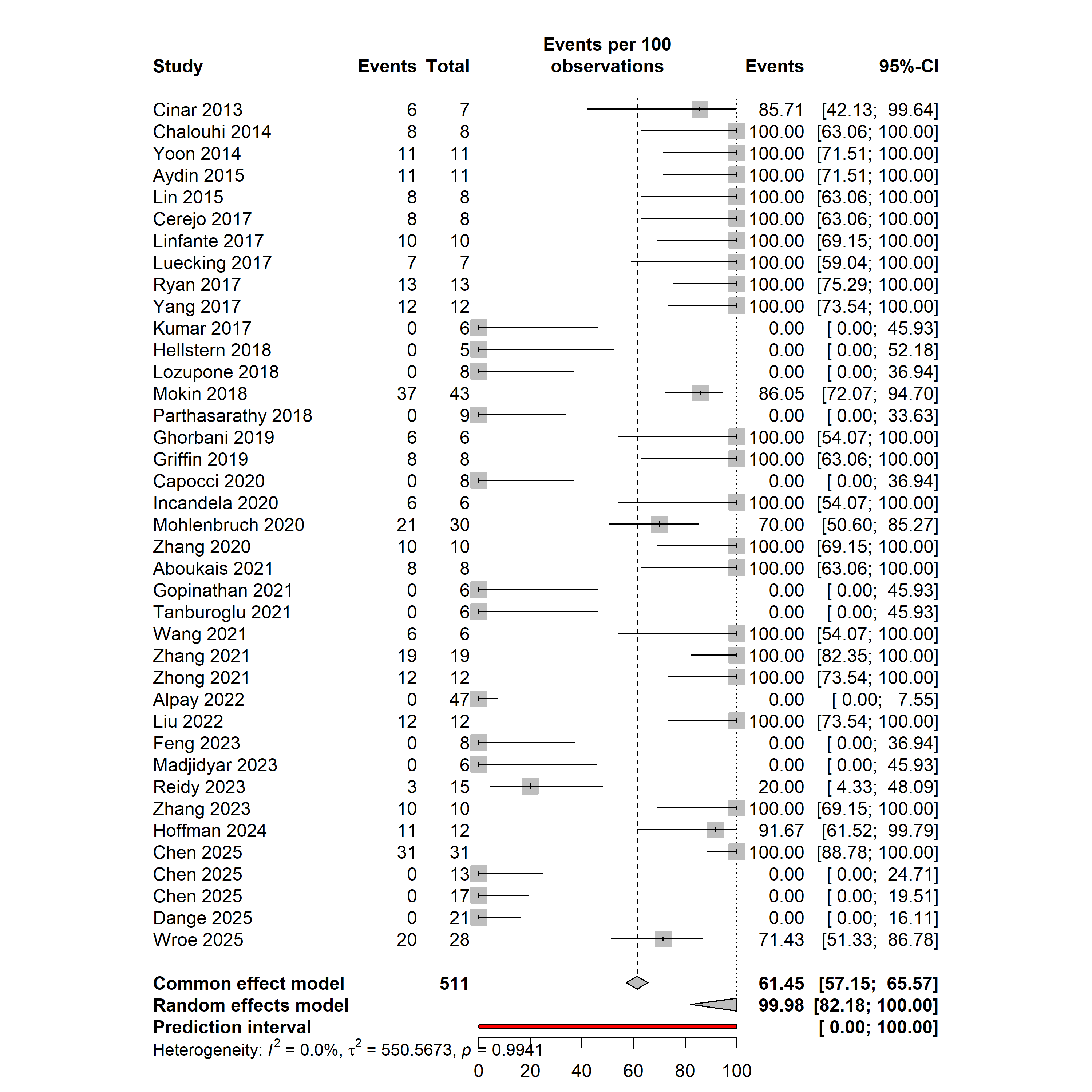


**Supplementary Figure 15.** Forest plot demonstrating the mean follow-up of patients in months.
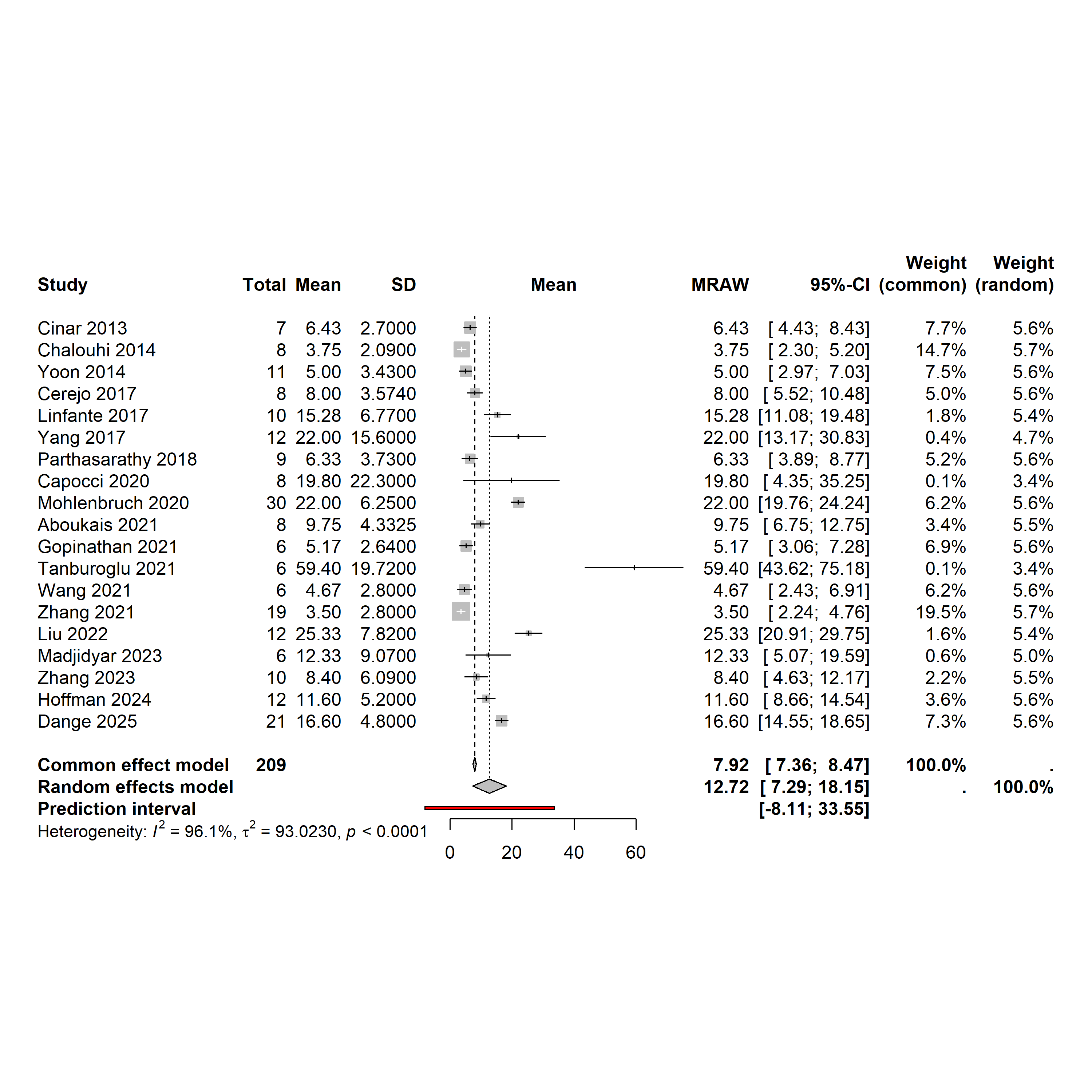


**Supplementary Figure 16.** Forest plot demonstrating proportion of patients with remnant aneurysm following treatment.
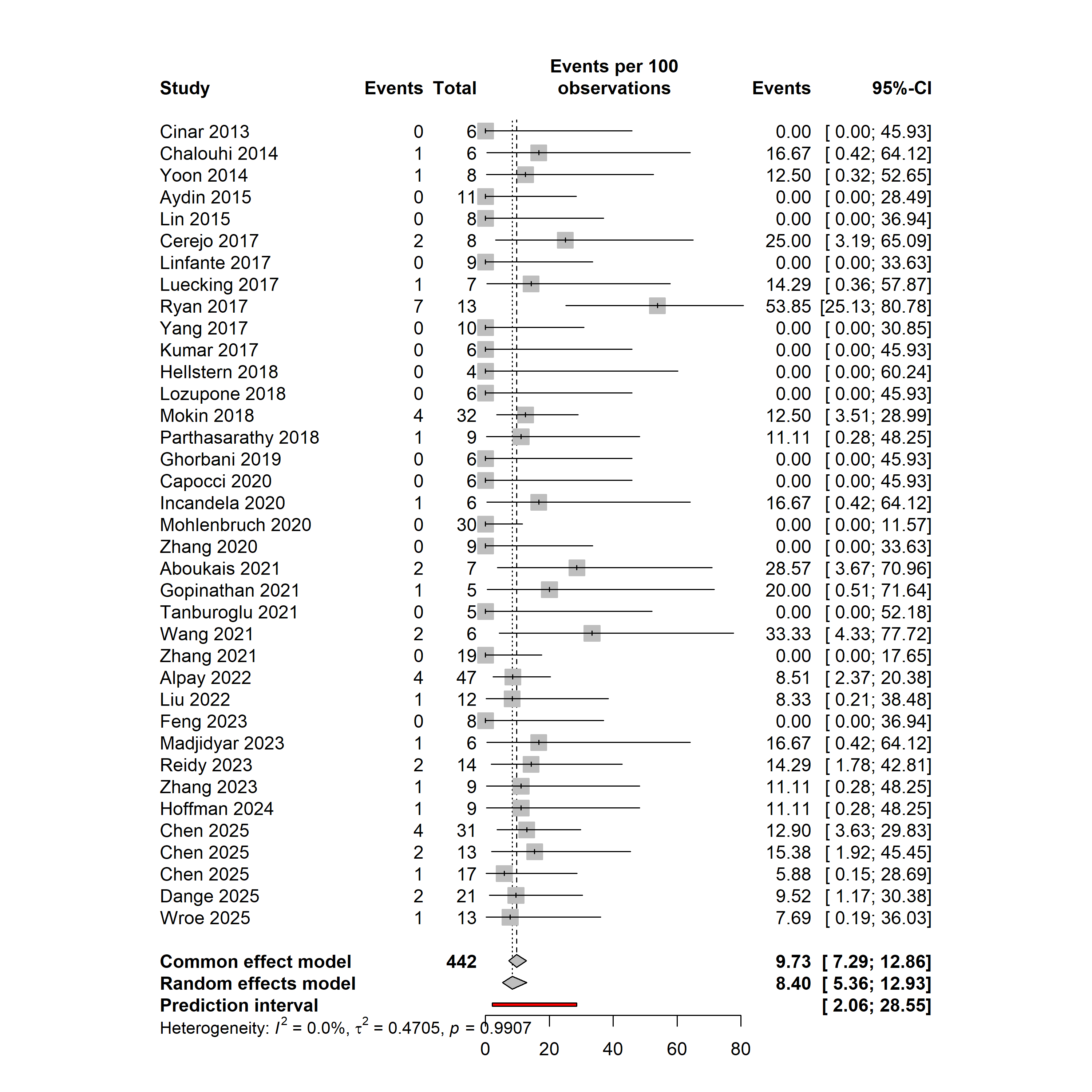


**Supplementary Figure 17.** Forest plot demonstrating leave-one-out analysis of complete occlusion rates.


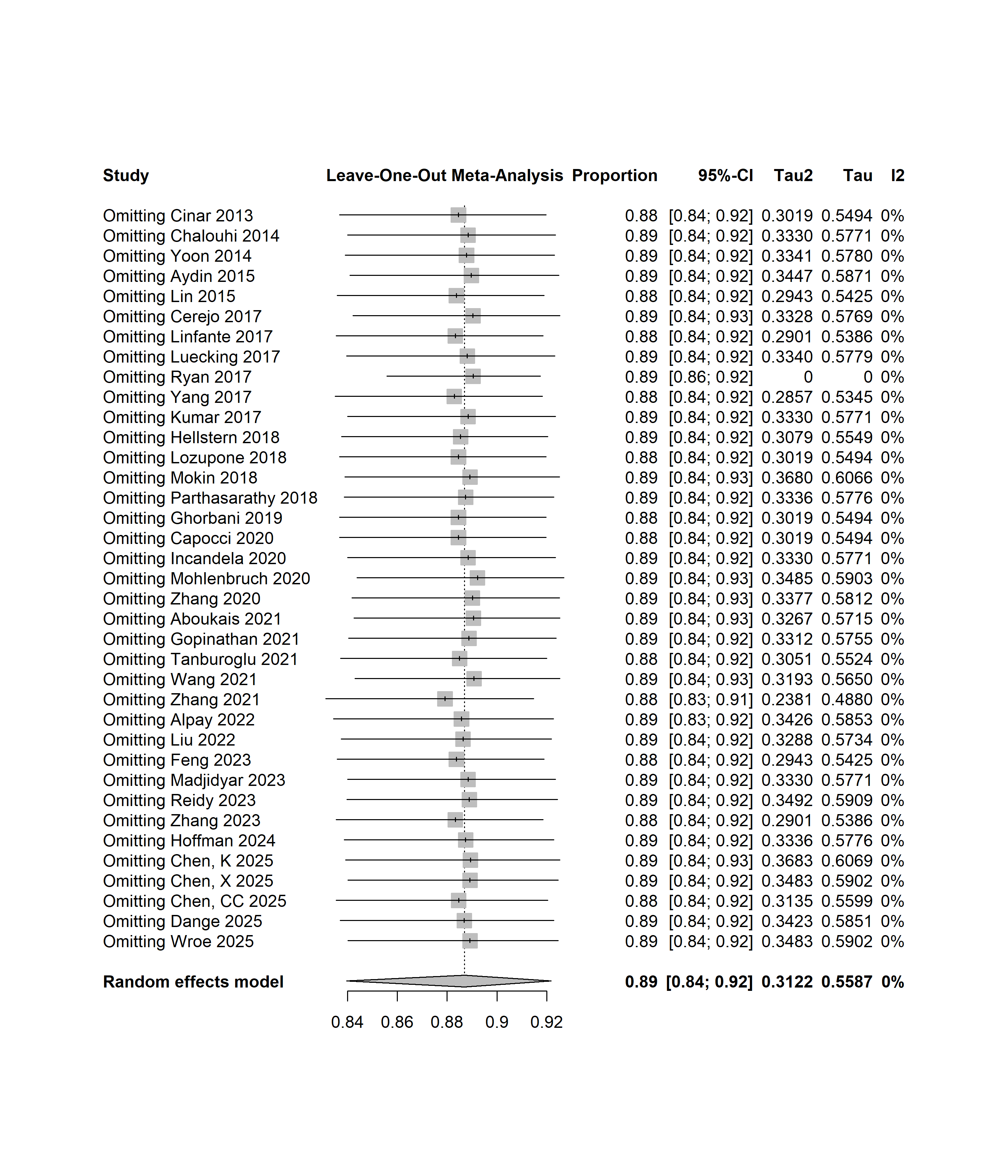


**Supplementary Figure 18.** Forest plot demonstrating leave-one-out analysis of remnant aneurysm rates.
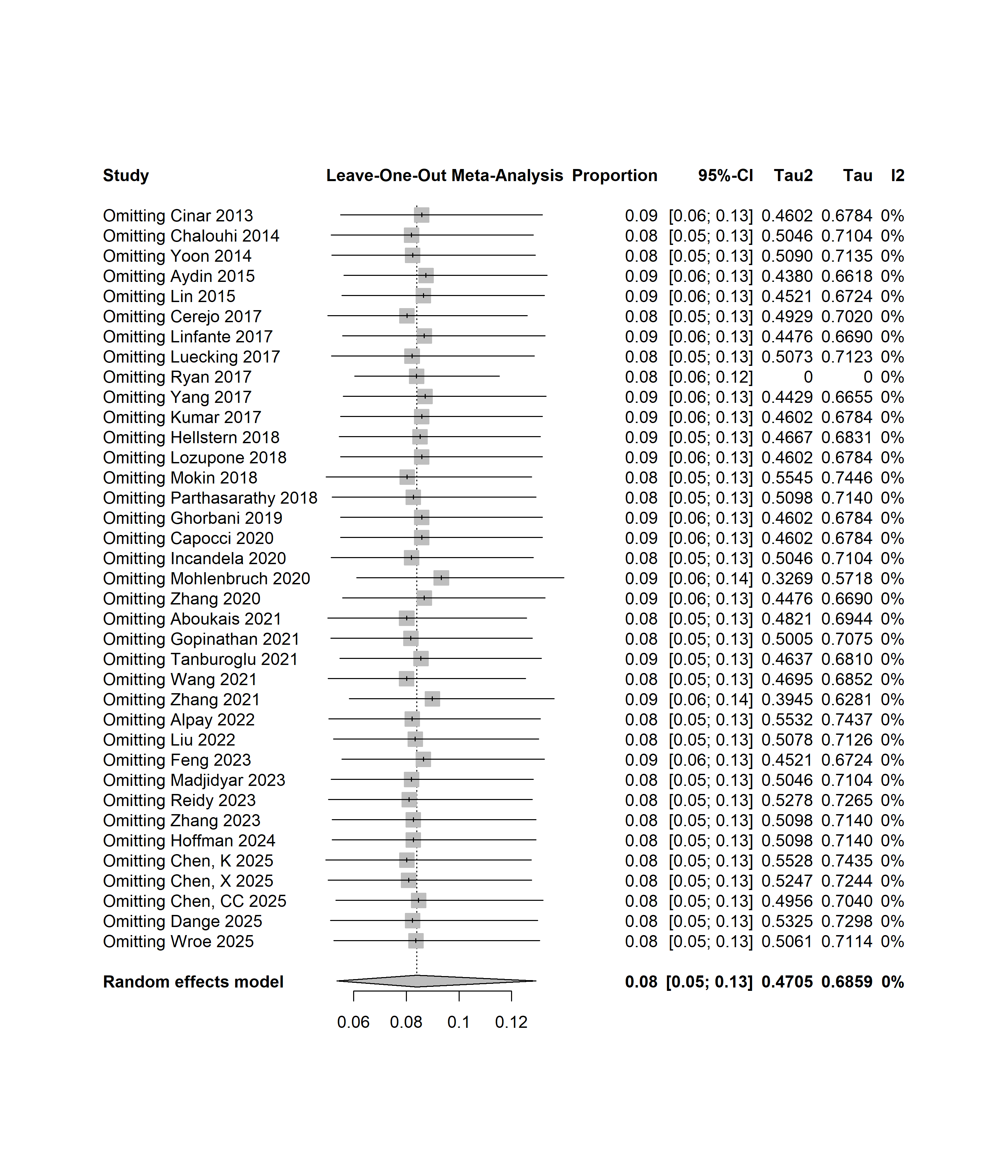


**Supplementary Figure 19**. Forest plot demonstrating the results of a subgroup analysis comparing complete occlusion rates between studies using different occlusion criteria (OC).
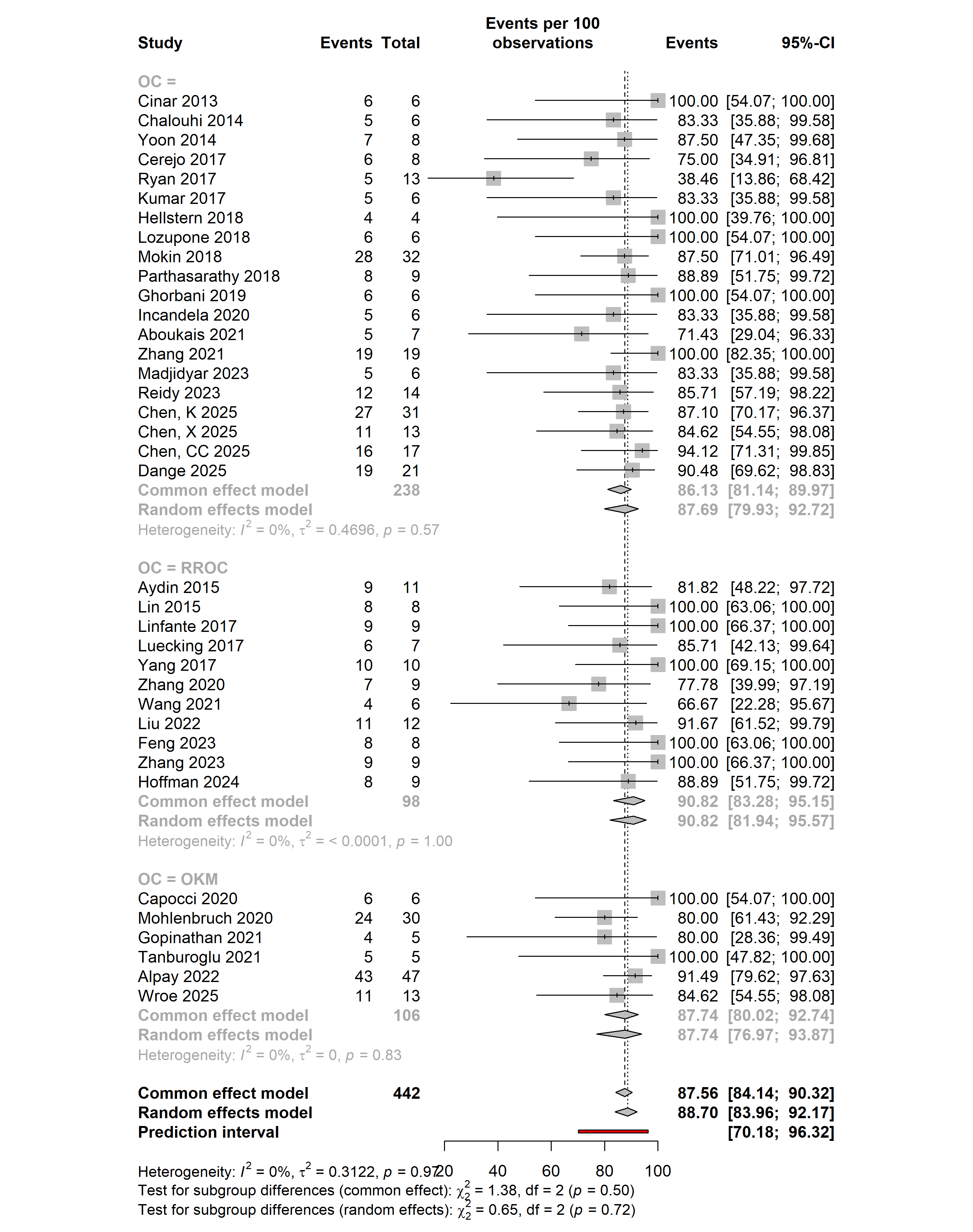


**Supplementary Figure 20.** Forest plot demonstrating proportion of patients with good neurological outcome.
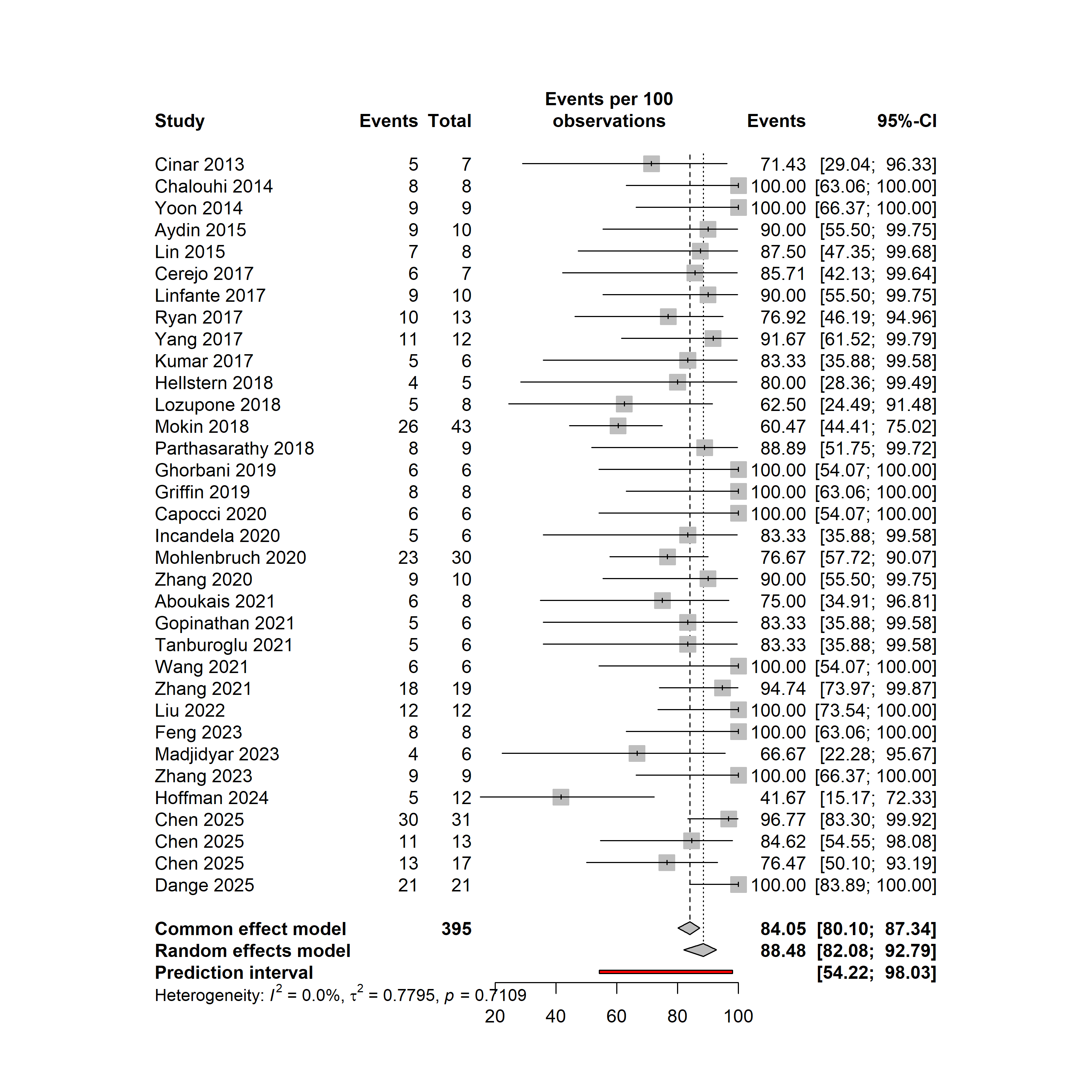


**Supplementary Figure 21.** Forest plot demonstrating leave-one-out analysis of good neurological outcome rates.
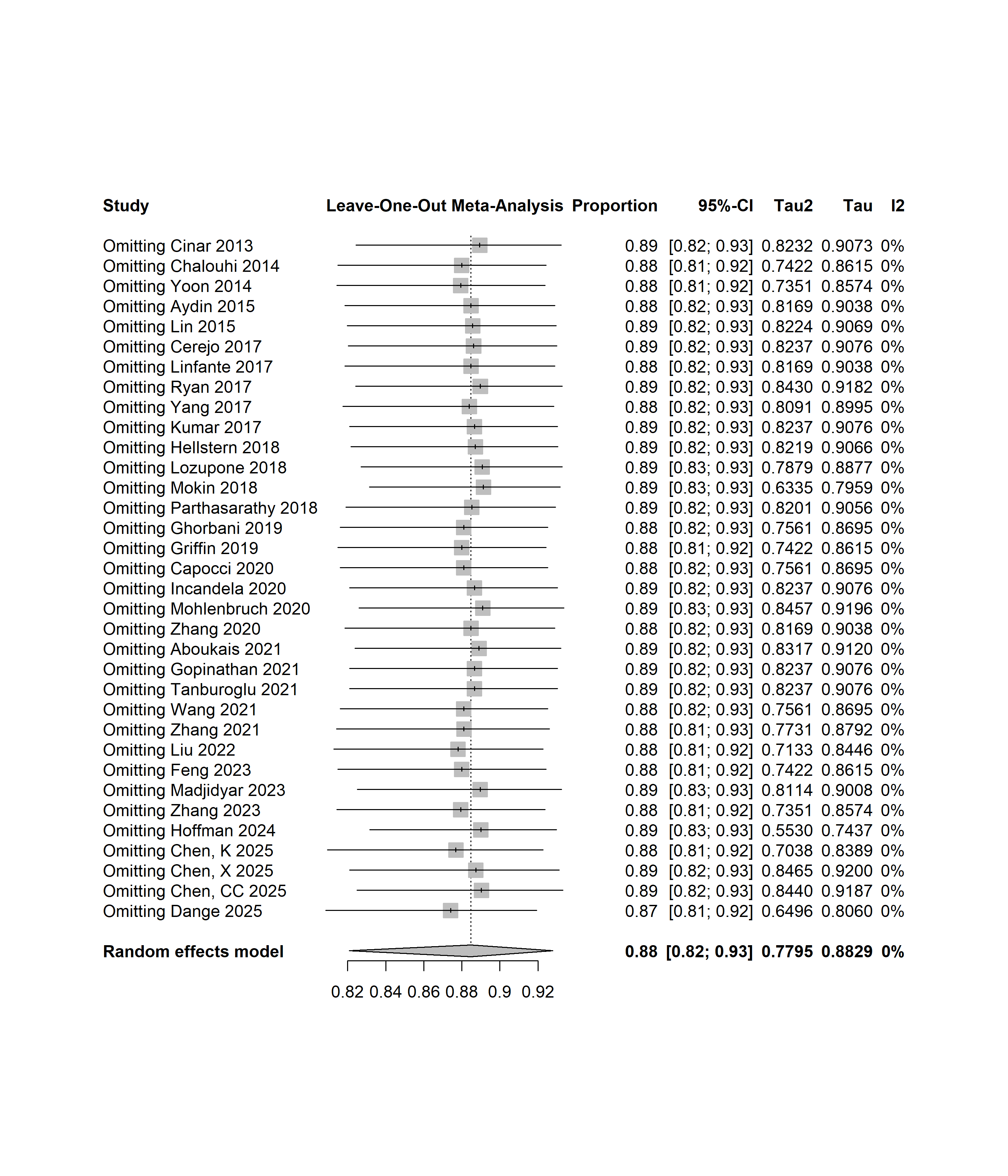


**Supplementary Figure 22.** Forest plot demonstrating proportion of patients requiring post-operative CSF diversion.
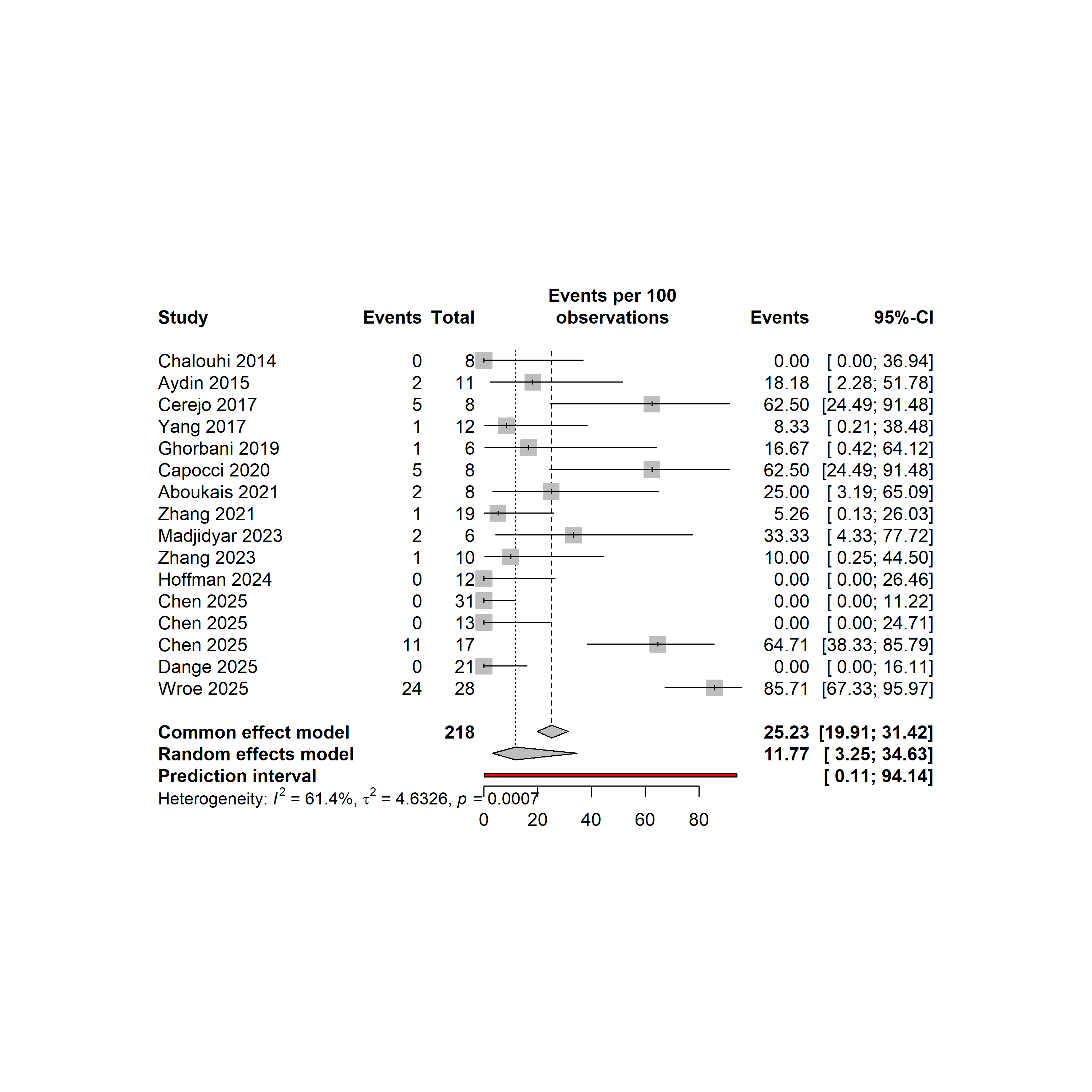


**Supplementary Figure 23.** Forest plot demonstrating proportion of patients requiring aneurysm retreatment.
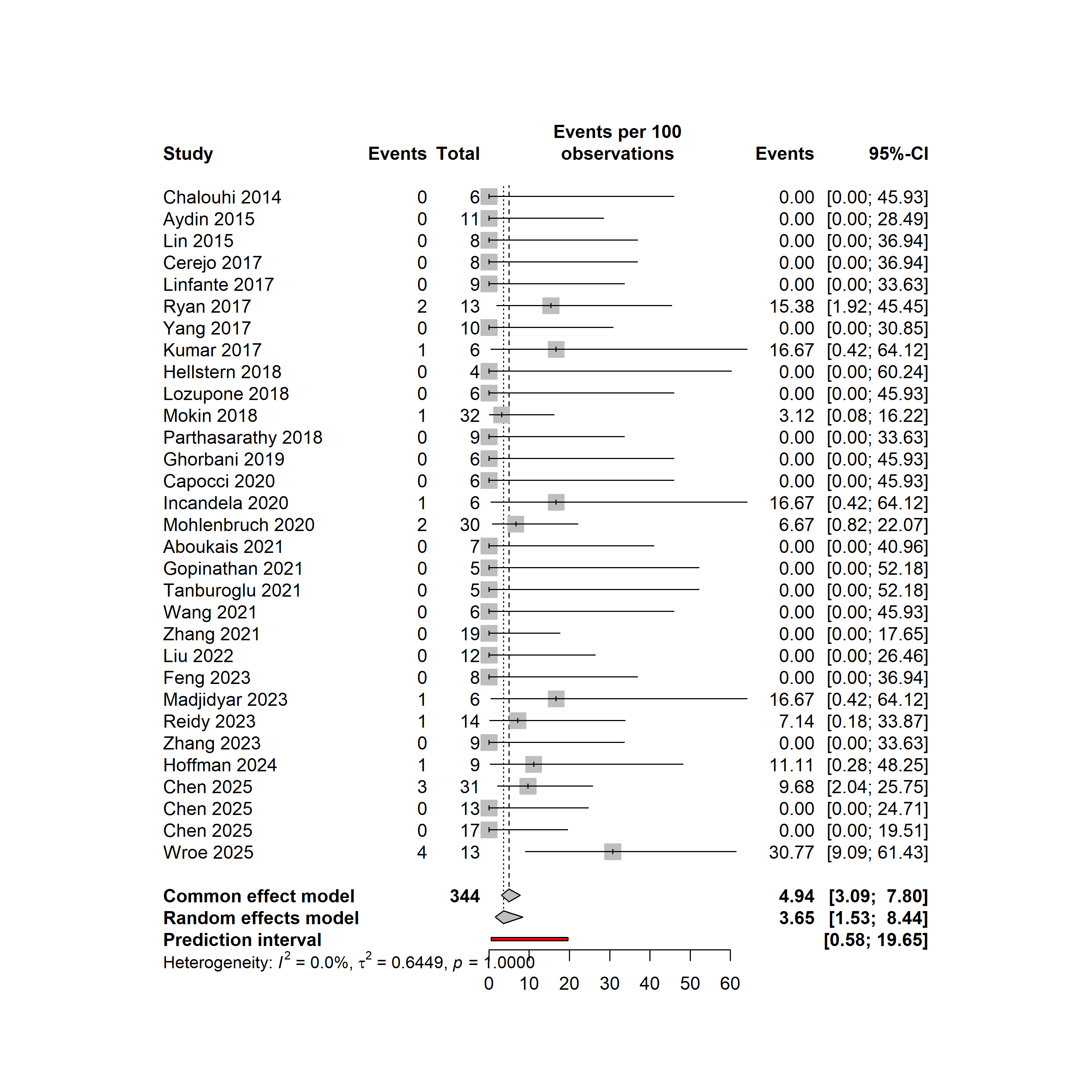


**Supplementary Figure 24**. Forest plot demonstrating the results of a subgroup analysis comparing complete occlusion rates between small and large studies.
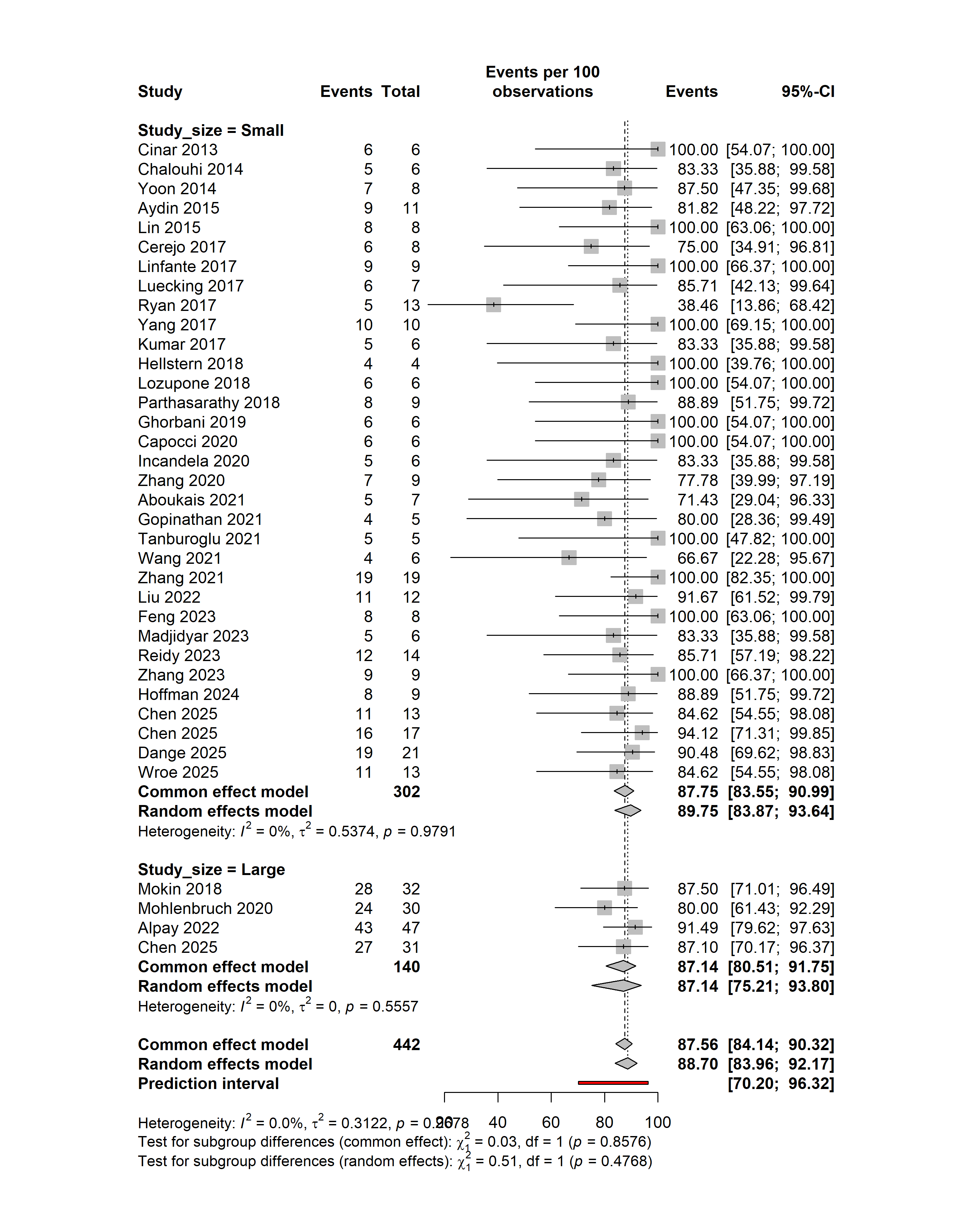


**Supplementary Figure 25**. Funnel plot of included studies on complete occlusion.
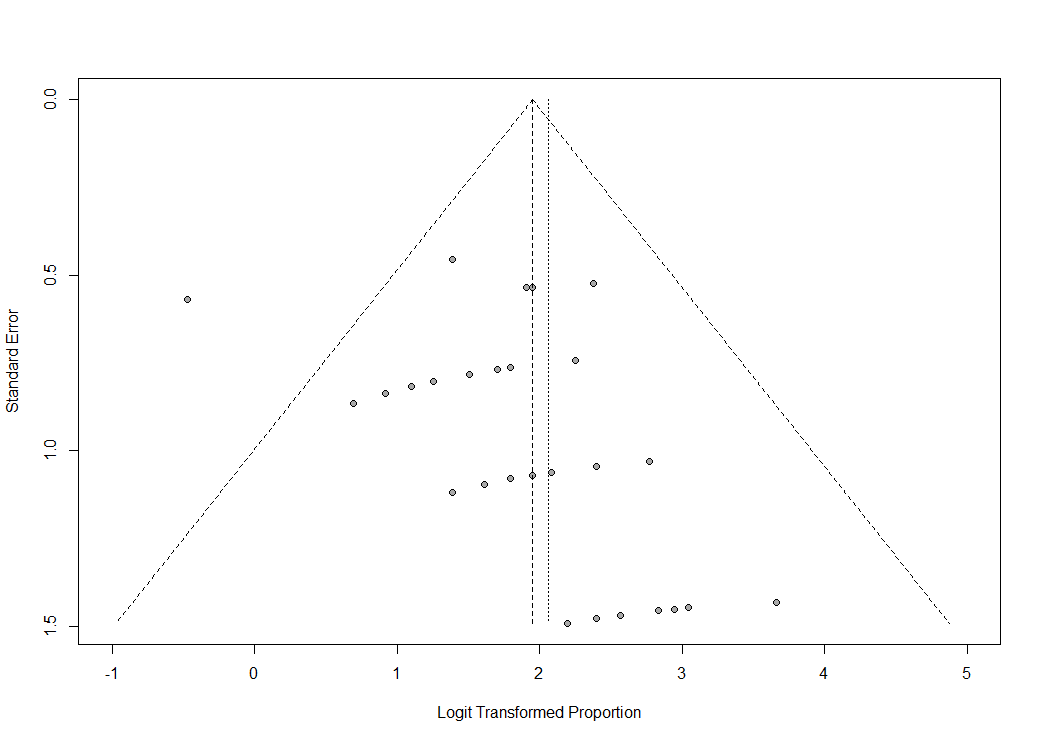


**Supplementary Figure 26**. Forest plot demonstrating the results of a subgroup analysis comparing good neurological outcome rates between small and large studies.
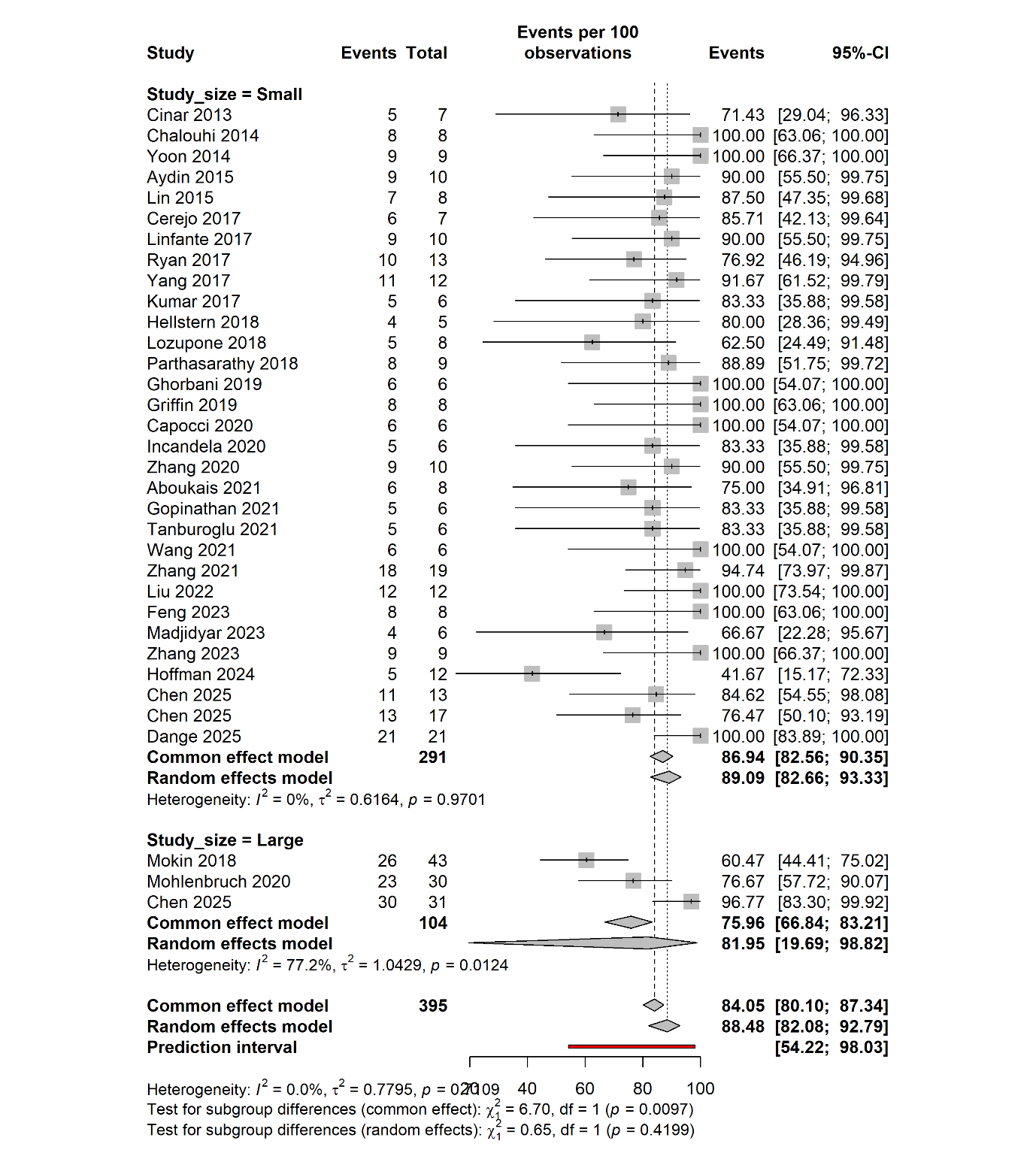


**Supplementary Figure 27**. Funnel plot of included studies on good neurological outcome.
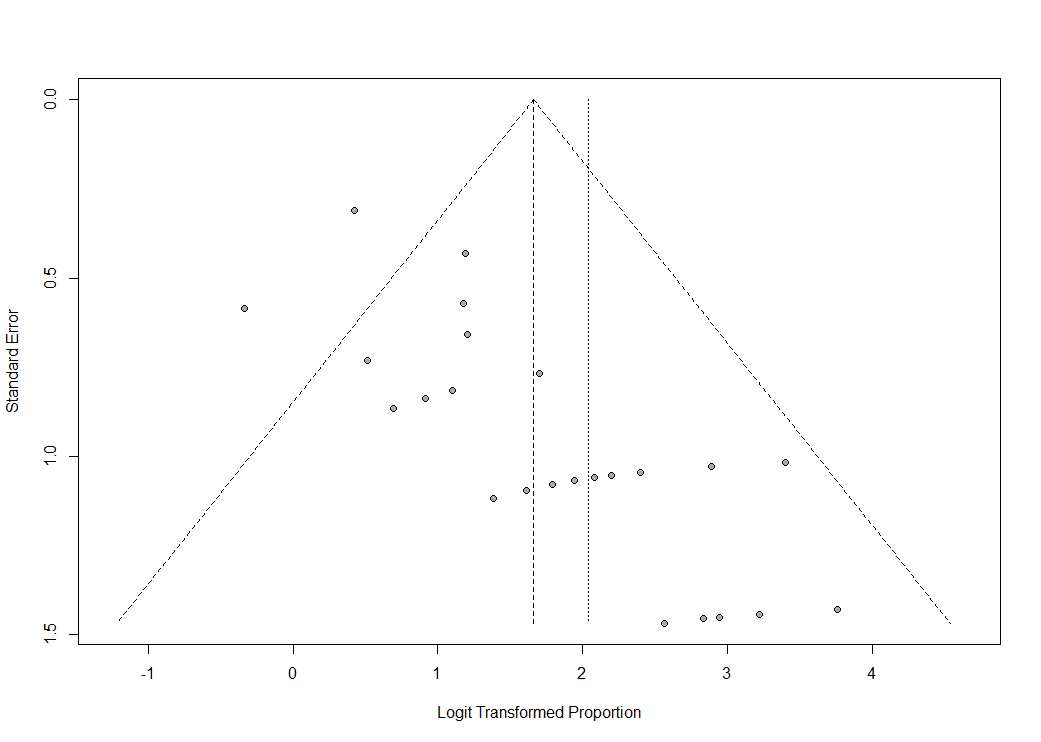


**Supplementary Figure 28**. Baujat plot identifying studies contributing to heterogeneity and overall effect size in the meta-analysis of complete occlusion rates.
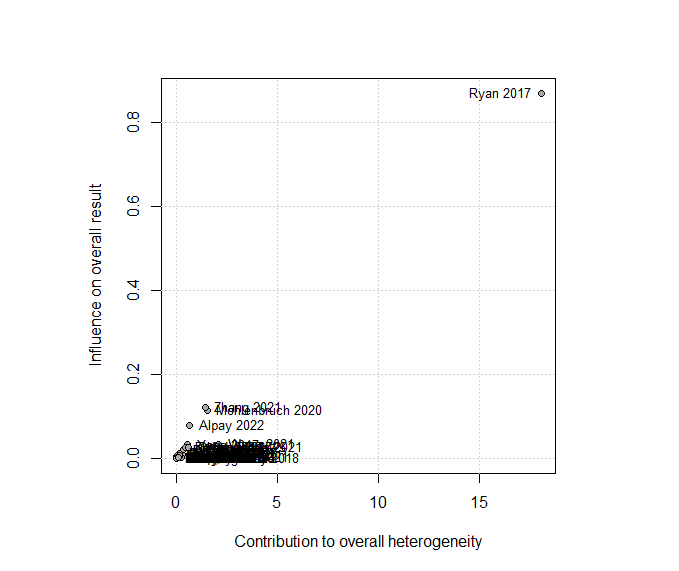


**Supplementary Figure 29**. Baujat plot identifying studies contributing to heterogeneity and overall effect size in the meta-analysis of good neurological outcome rates.
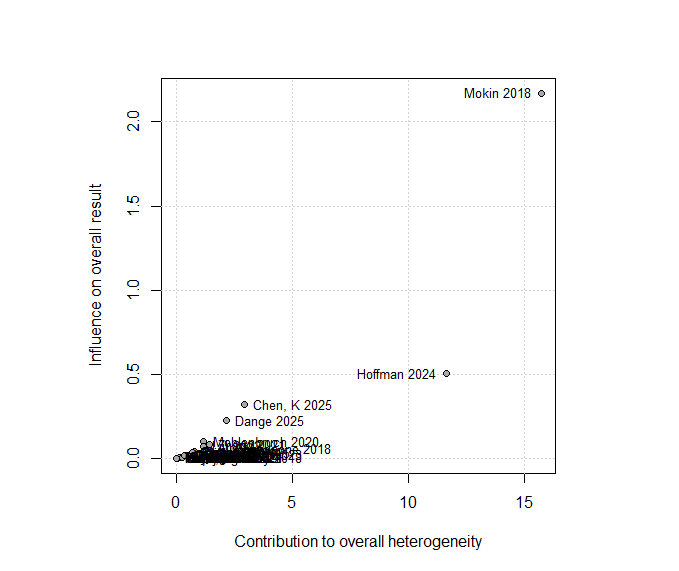


**Supplementary Figure 30**. Forest plot demonstrating proportion of patients experiencing intraoperative aneurysm rupture.
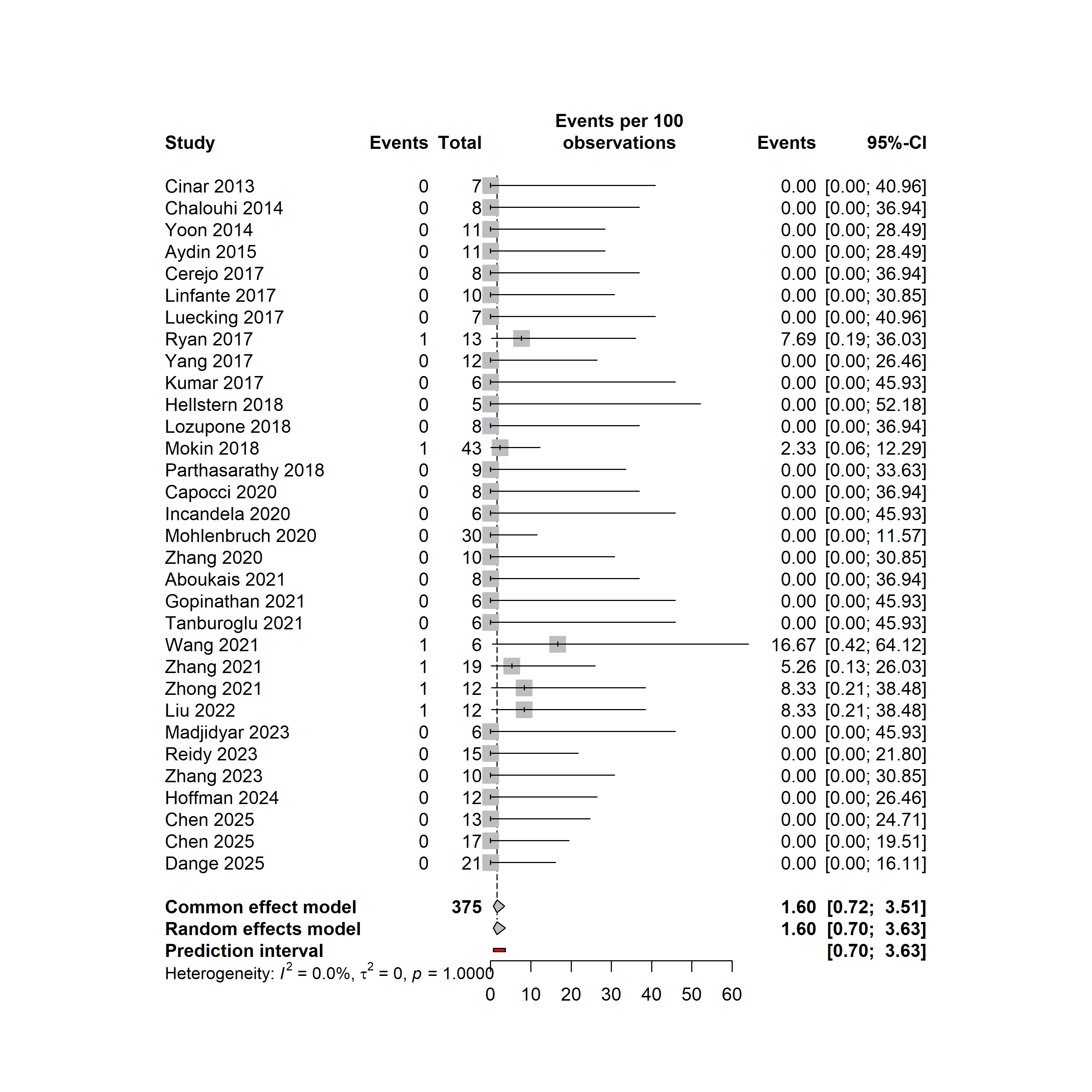


**Supplementary Figure 31**. Forest plot demonstrating proportion of patients experiencing hemorrhagic complications following treatment.
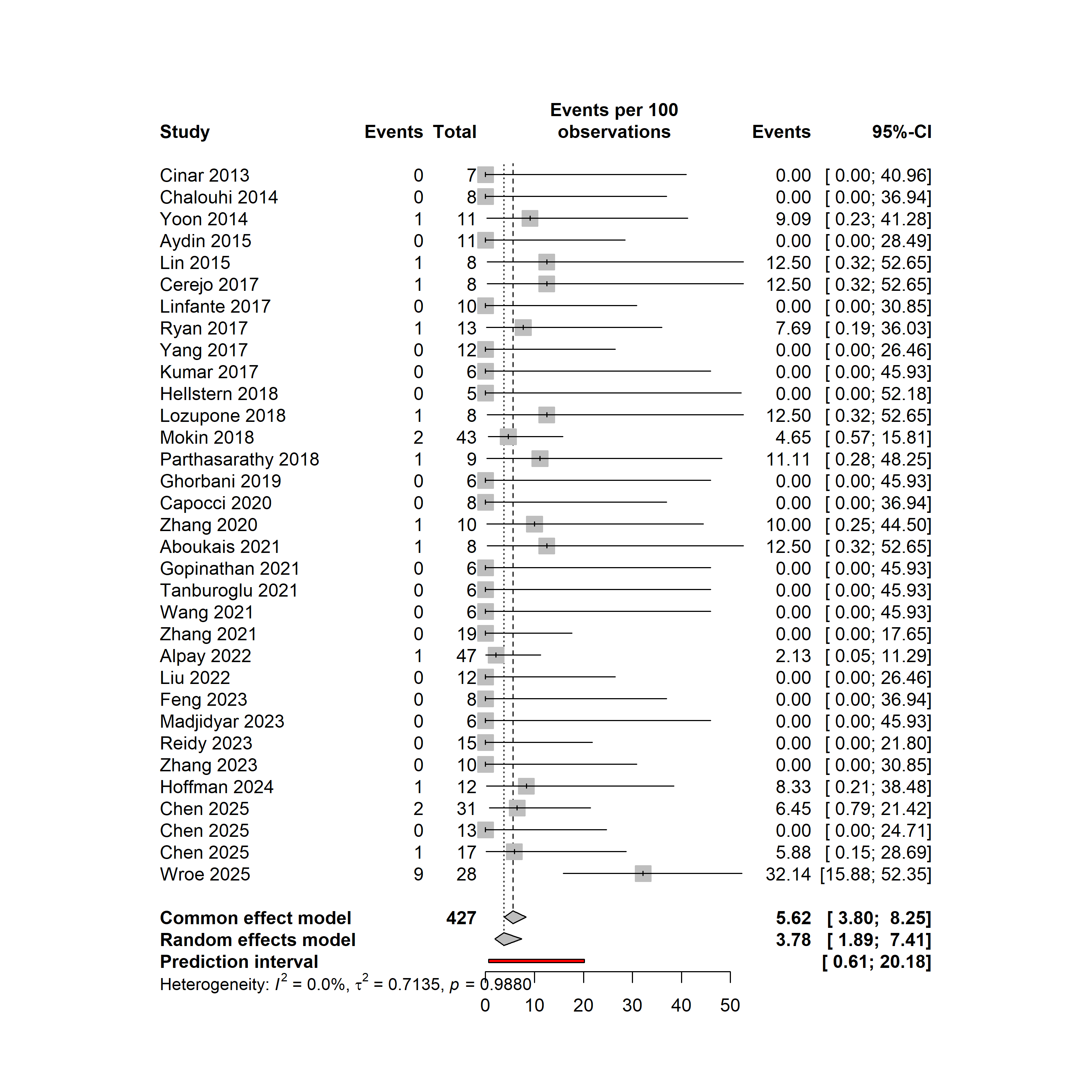


**Supplementary Figure 32.** Forest plot demonstrating proportion of patients experiencing thromboembolic complications following treatment.
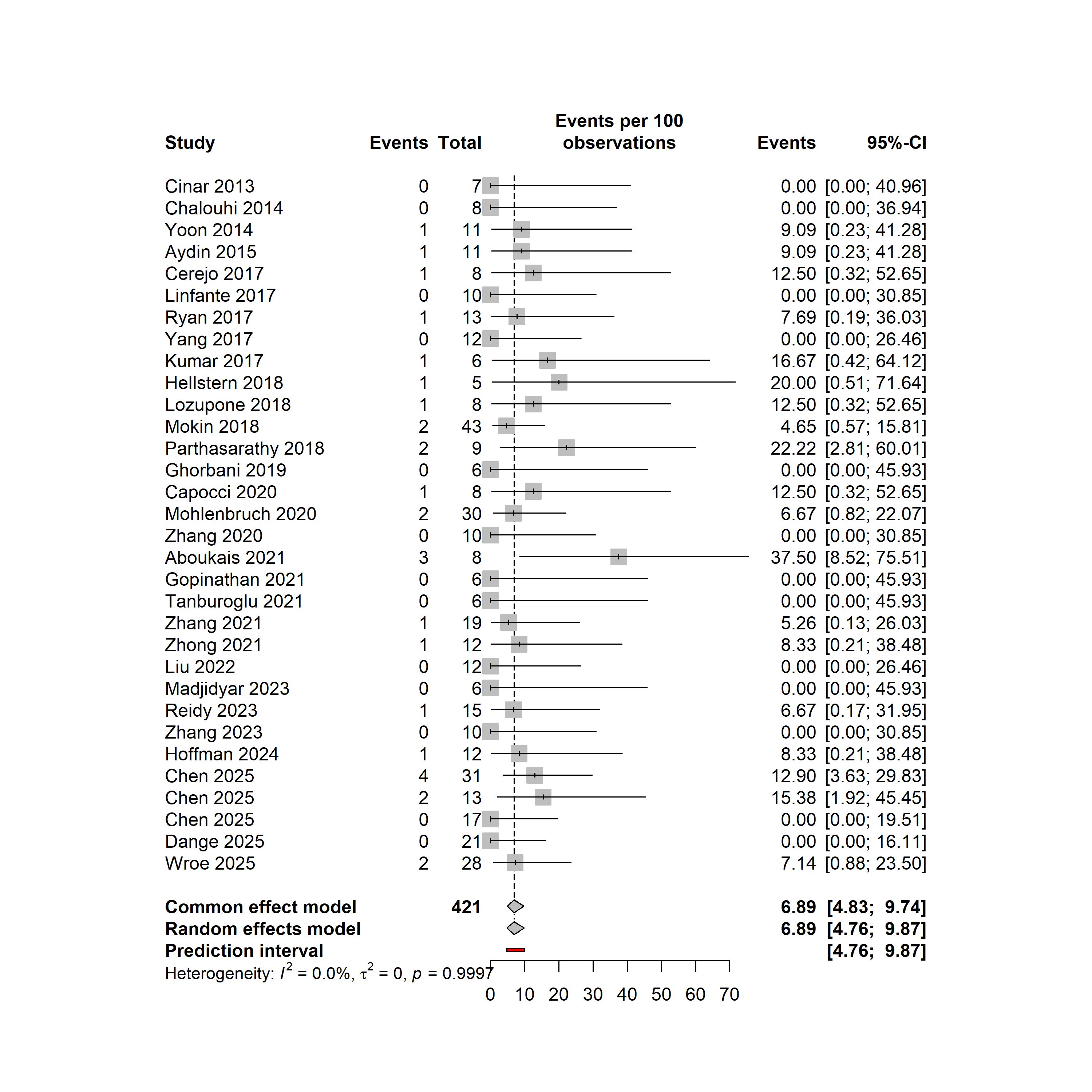


**Supplementary Figure 33.** Forest plot demonstrating proportion of patients experiencing neurologic complications following treatment.
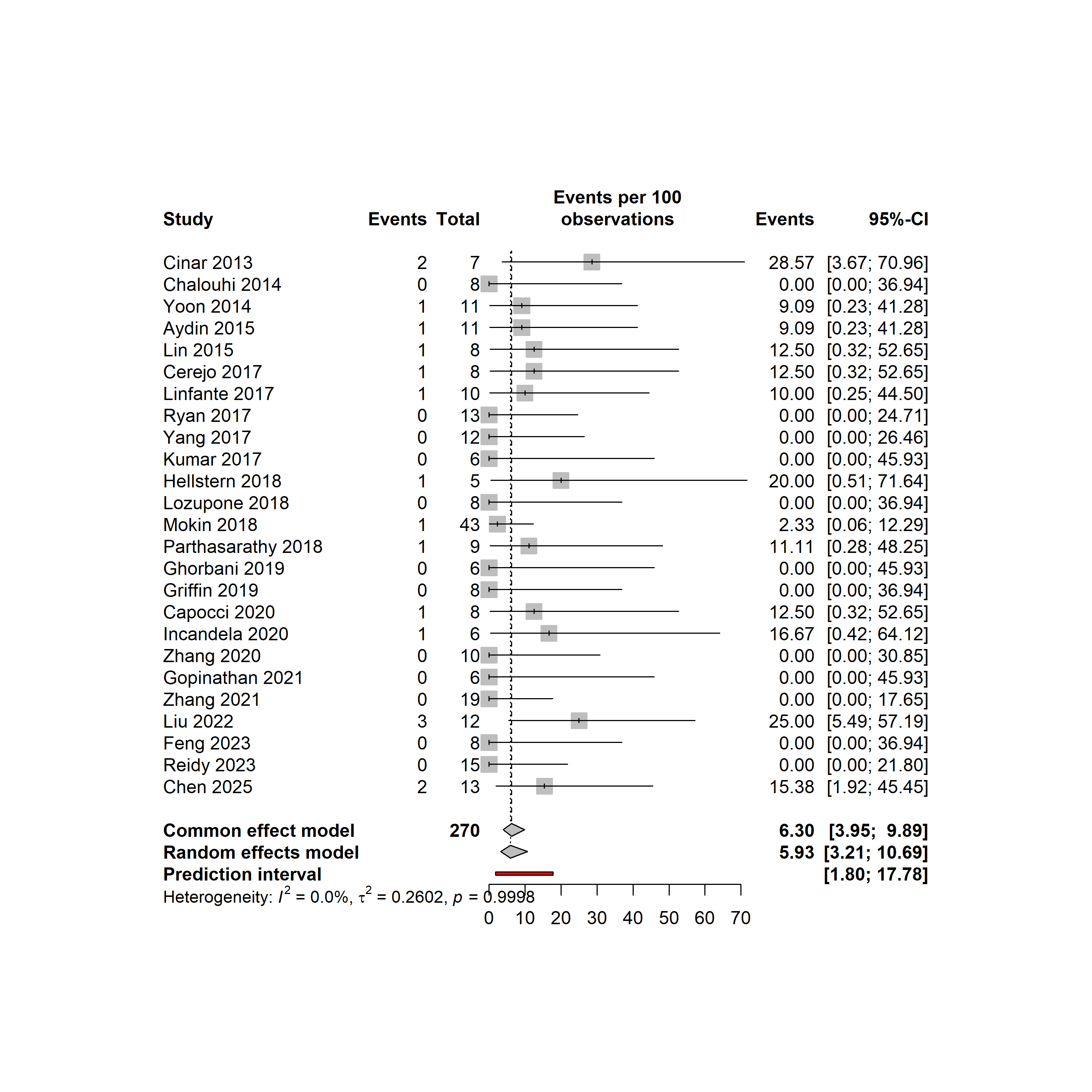


**Supplementary Figure 34.** Forest plot demonstrating proportion of patients experiencing vasospasm following treatment.
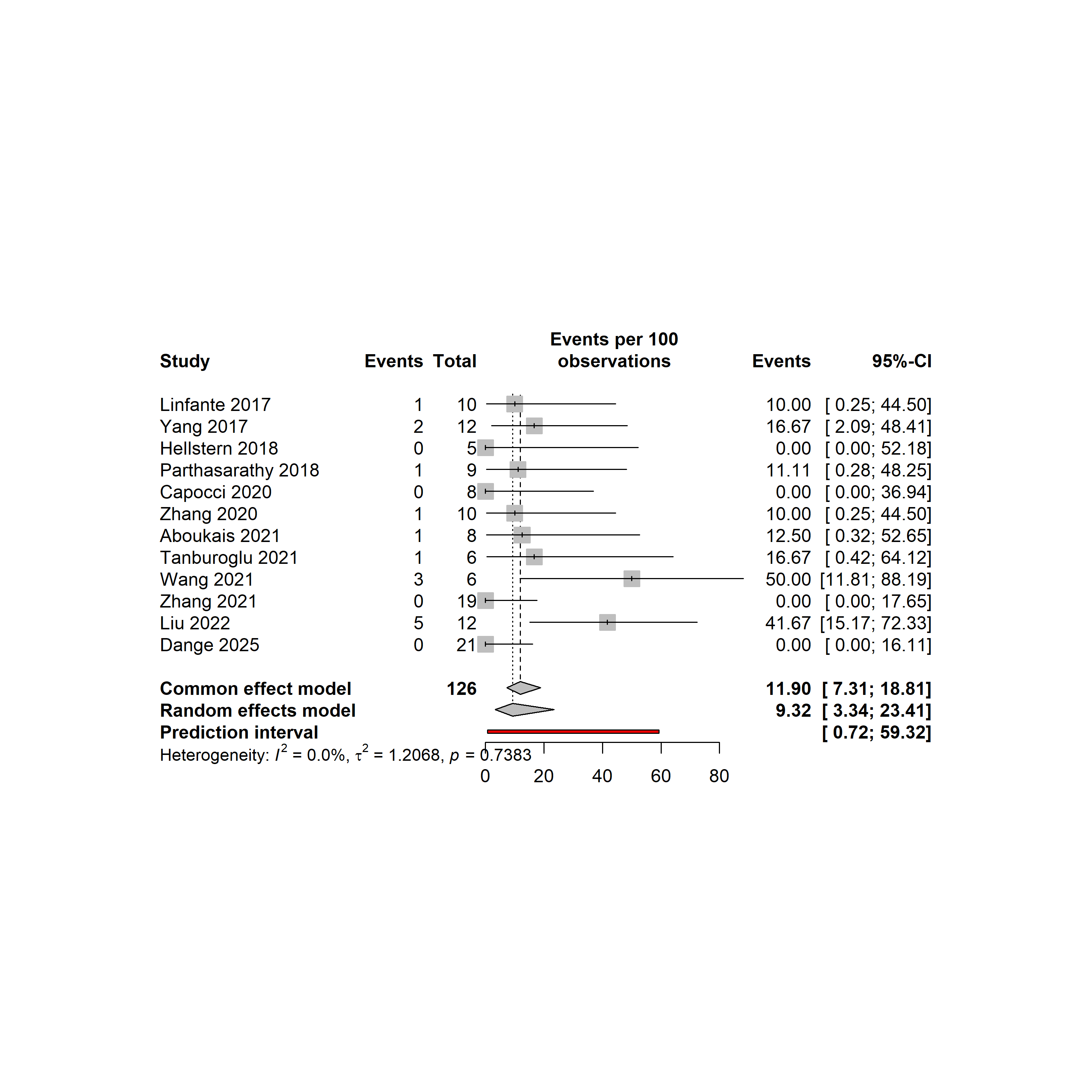


**Supplementary Figure 35.** Forest plot demonstrating proportion of patients experiencing in-stent stenosis.
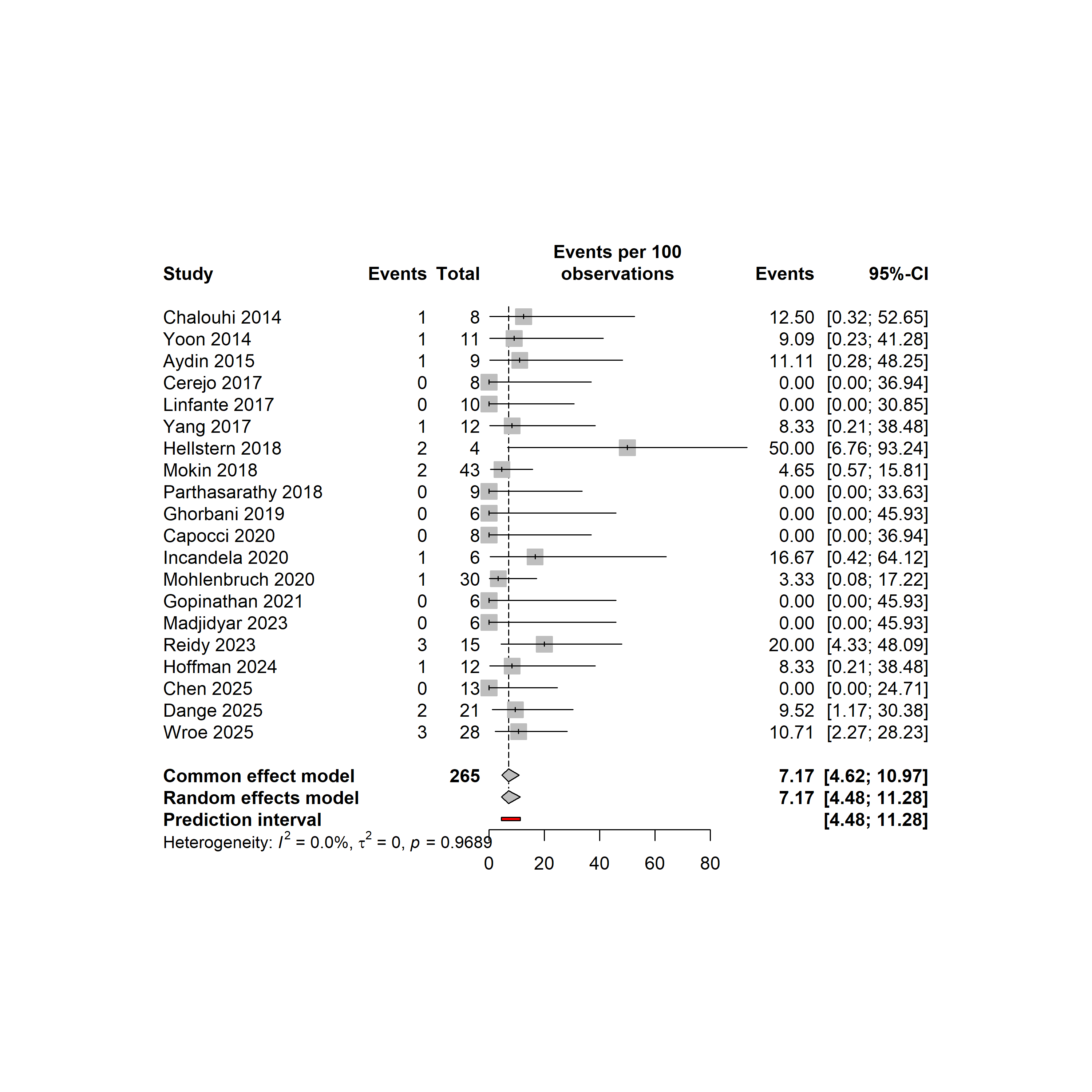


**Supplementary Figure 36.** Forest plot demonstrating mortality rates following treatment.
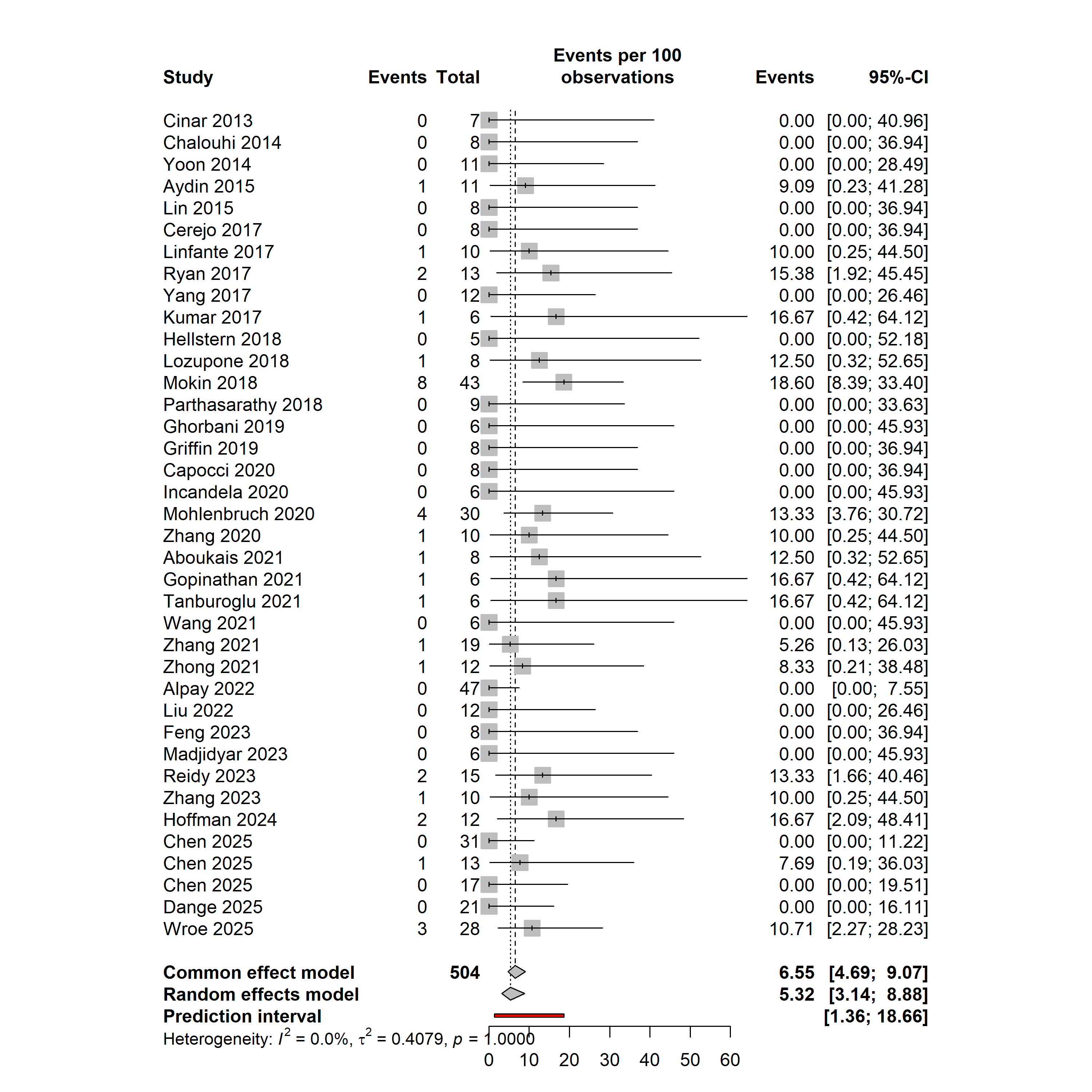


**Supplementary Figure 37**. Forest plot demonstrating the results of a subgroup analysis comparing intraoperative aneurysm rupture rates between small and large studies.
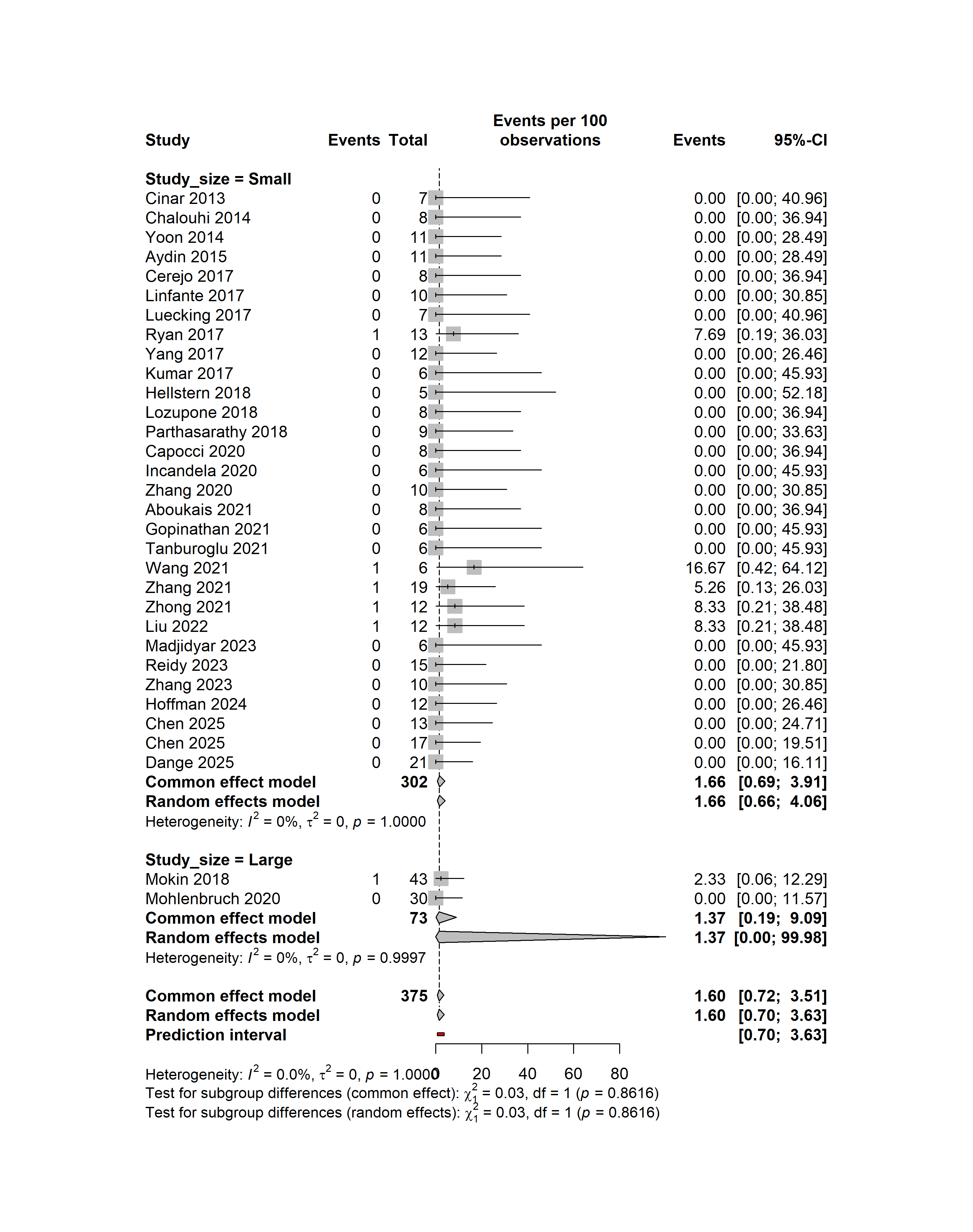


**Supplementary Figure 38**. Forest plot demonstrating the results of a subgroup analysis comparing hemorrhagic complication rates between small and large studies.


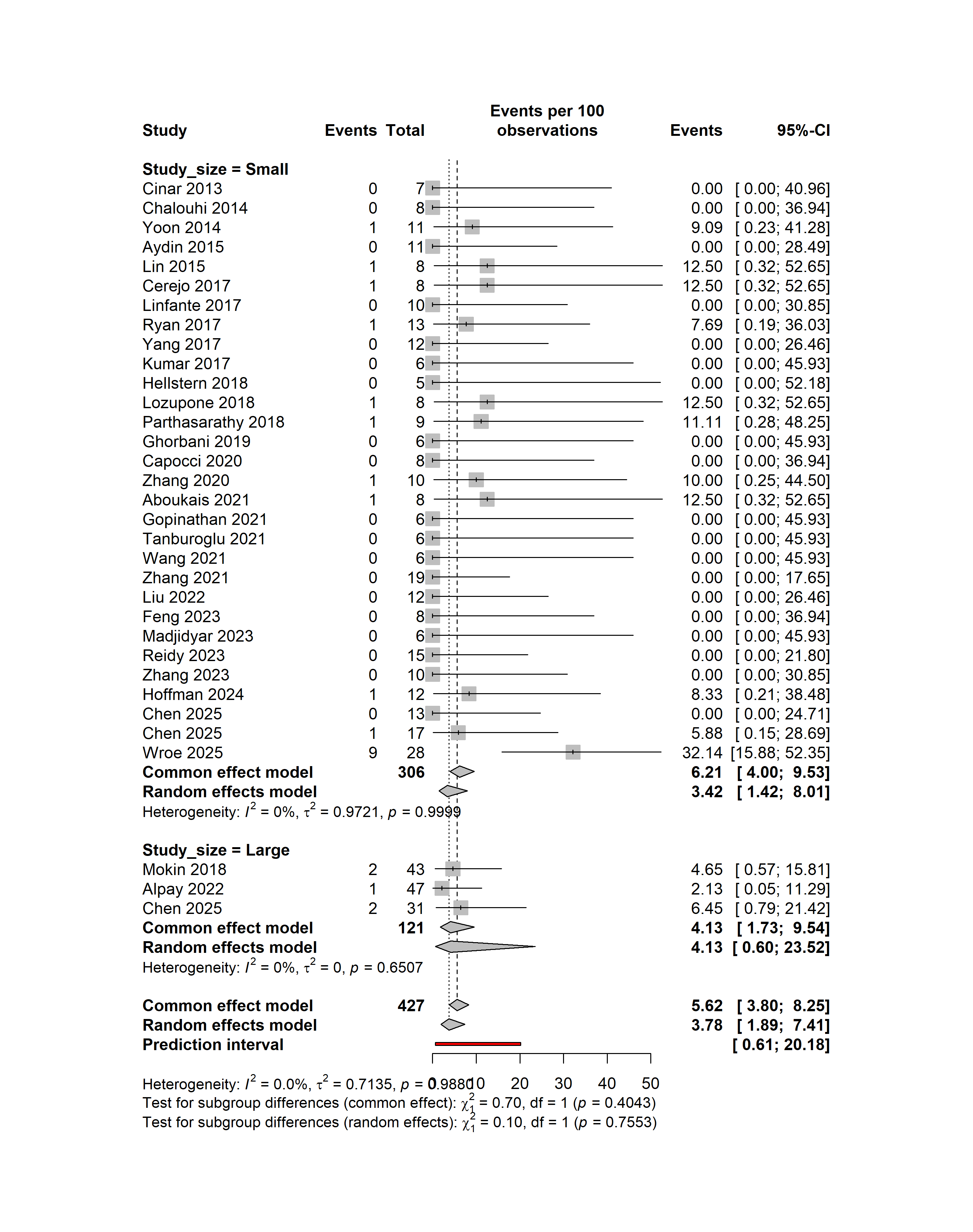


**Supplementary Figure 39**. Forest plot demonstrating the results of a subgroup analysis comparing thromboembolic complication rates between small and large studies.
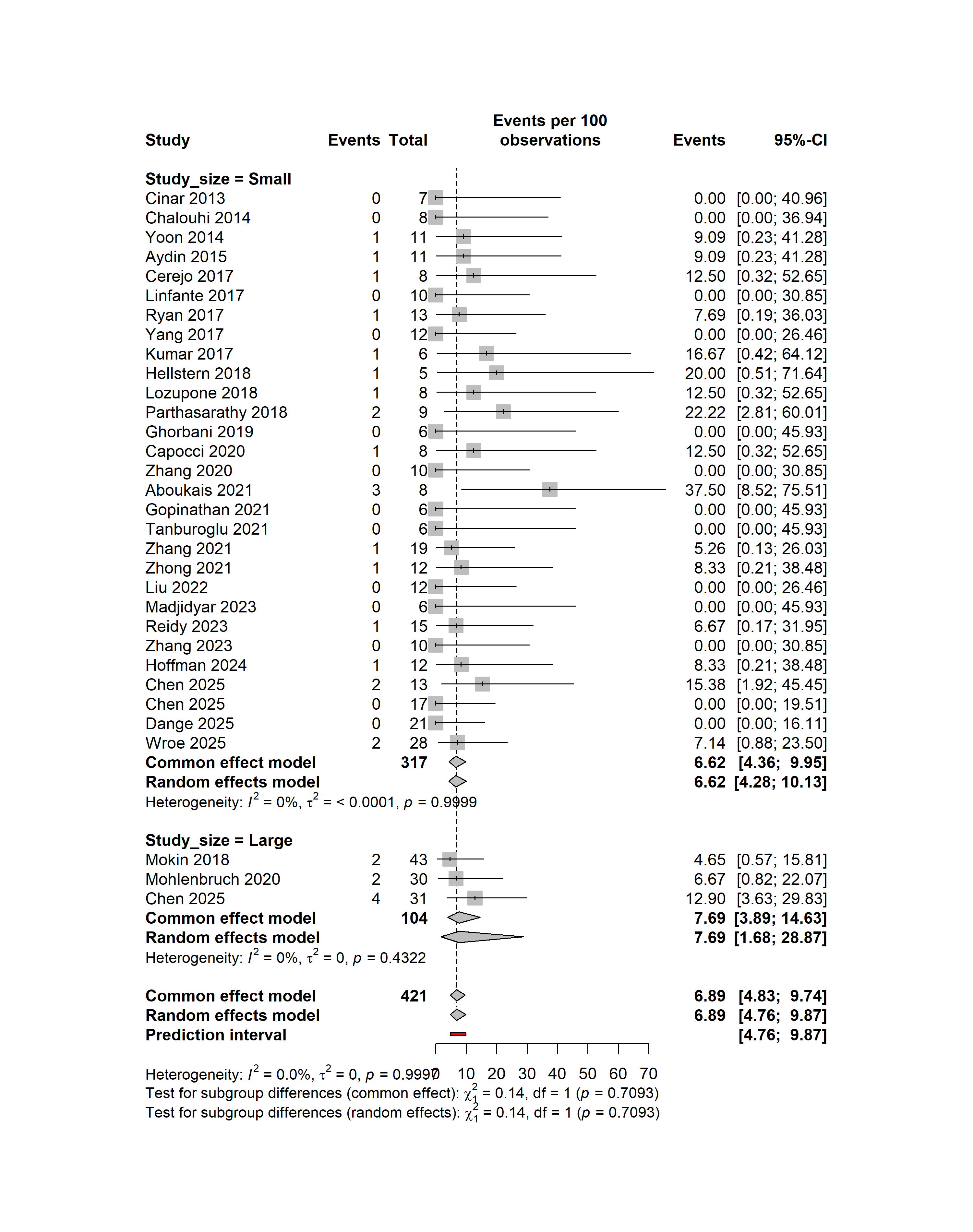


**Supplementary Figure 40**. Forest plot demonstrating the results of a subgroup analysis comparing in-stent stenosis rates between small and large studies
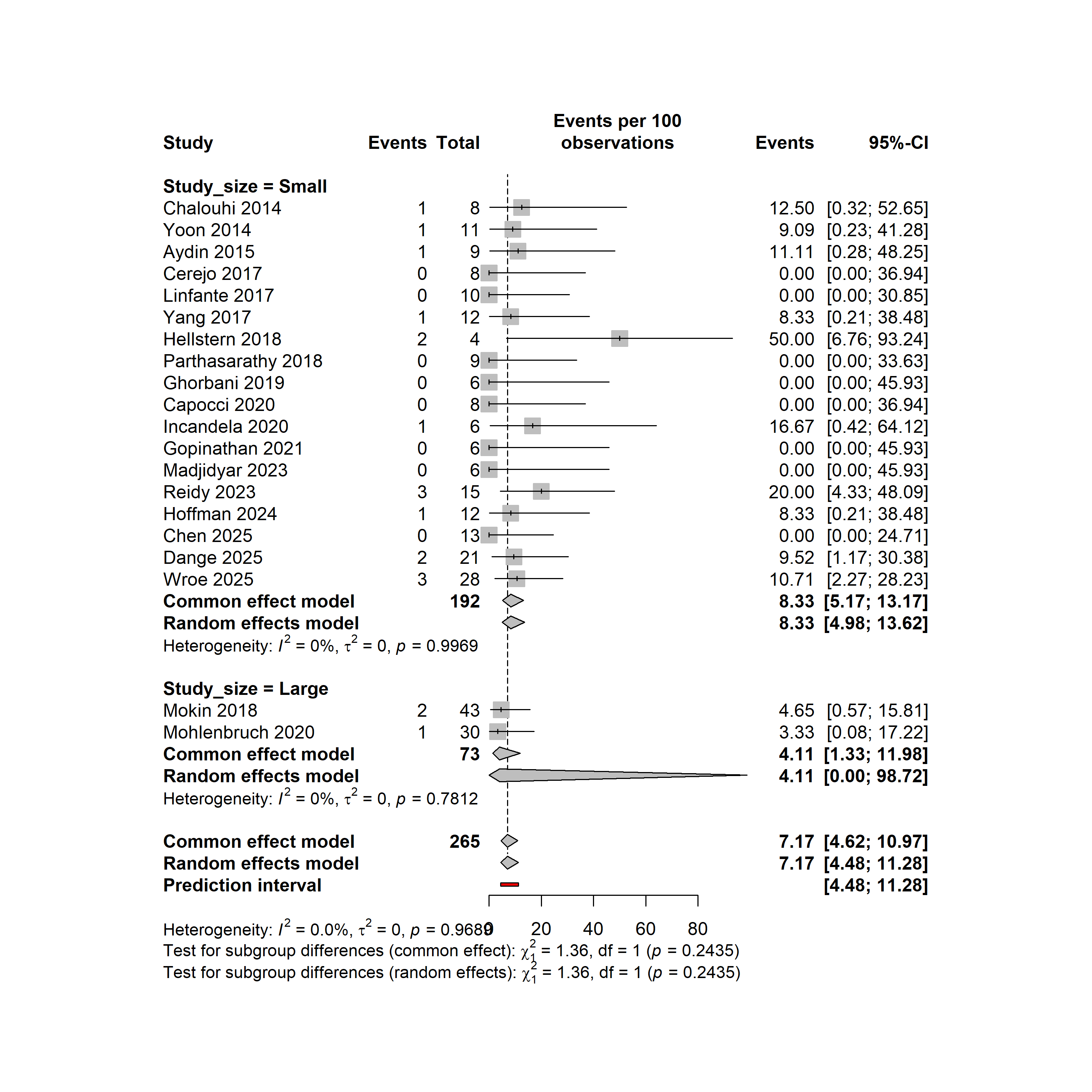


**Supplementary Figure 41**. Forest plot demonstrating the results of a subgroup analysis comparing mortality rates between small and large studies.
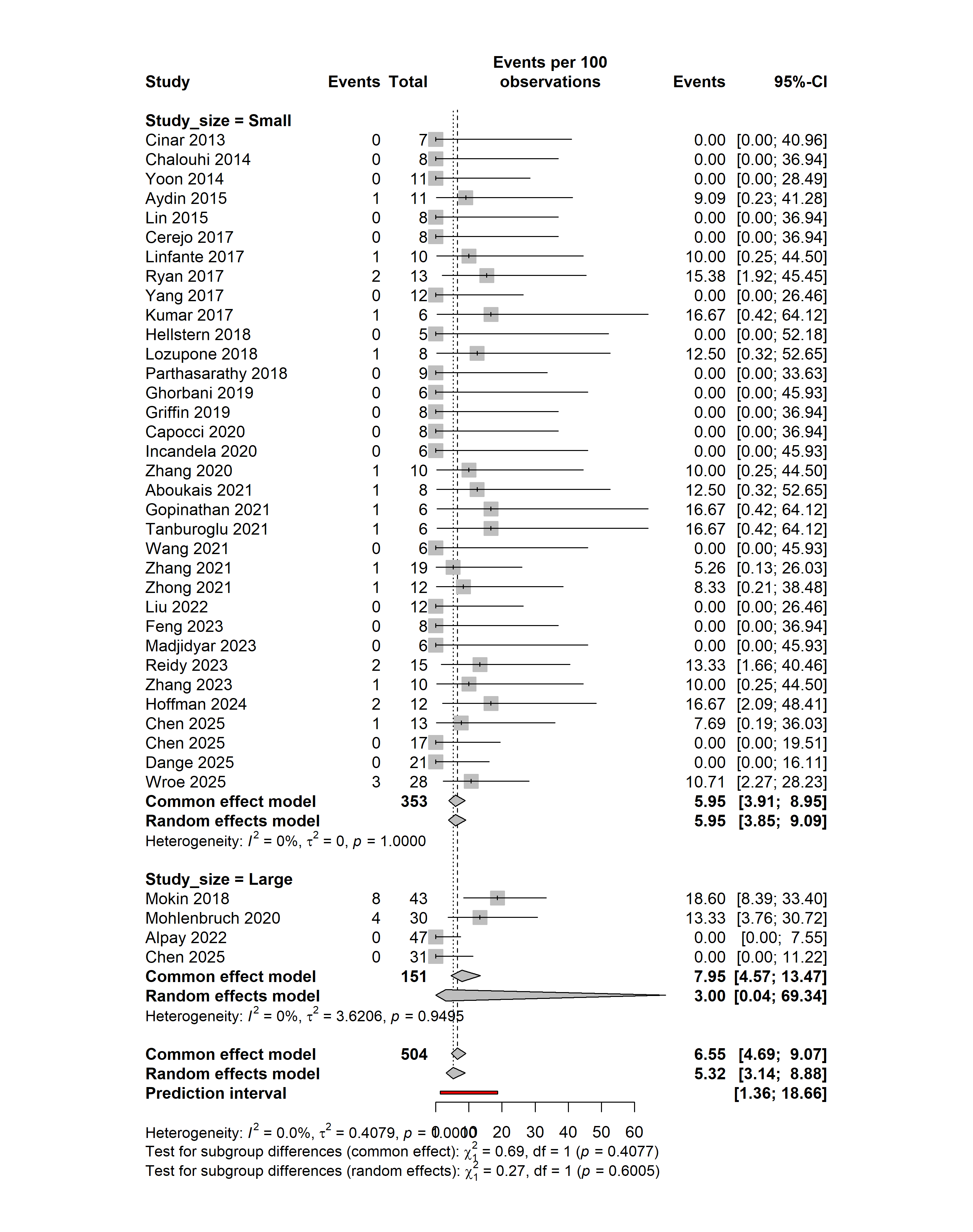


**References**

[1] C. Çinar, İ. Oran, H. Bozkaya, and E. Ozgiray, "Endovascular treatment of ruptured blister-like aneurysms with special reference to the flow-diverting strategy," (in eng), *Neuroradiology,* vol. 55, no. 4, pp. 441-7, Mar 2013, doi: 10.1007/s00234-013-1136-y.

[2] M. Zanaty, N. Chalouhi, S. I. Tjoumakaris, R. H. Rosenwasser, L. F. Gonzalez, and P. Jabbour, "Flow-diversion panacea or poison?," (in eng), *Frontiers in neurology,* vol. 5, p. 21, 2014, doi: 10.3389/fneur.2014.00021.

[3] J. W. Yoon *et al.*, "Feasibility and safety of pipeline embolization device in patients with ruptured carotid blister aneurysms," (in eng), *Neurosurgery,* vol. 75, no. 4, pp. 419-29; discussion 429, Oct 2014, doi: 10.1227/neu.0000000000000487.

[4] K. Aydin *et al.*, "Treatment of ruptured blood blister-like aneurysms with flow diverter SILK stents," (in eng), *J Neurointerv Surg,* vol. 7, no. 3, pp. 202-9, Mar 2015, doi: 10.1136/neurintsurg-2013-011090.

[5] N. Lin *et al.*, "Utilization of Pipeline embolization device for treatment of ruptured intracranial aneurysms: US multicenter experience," (in eng), *J Neurointerv Surg,* vol. 7, no. 11, pp. 808-15, Nov 2015, doi: 10.1136/neurintsurg-2014-011320.

[6] R. Cerejo *et al.*, "Flow diverter treatment of cerebral blister aneurysms," (in eng), *Neuroradiology,* vol. 59, no. 12, pp. 1285-1290, Dec 2017, doi: 10.1007/s00234-017-1936-6.

[7] I. Linfante, M. Mayich, A. Sonig, J. Fujimoto, A. Siddiqui, and G. Dabus, "Flow diversion with Pipeline Embolic Device as treatment of subarachnoid hemorrhage secondary to blister aneurysms: dual-center experience and review of the literature," (in eng), *J Neurointerv Surg,* vol. 9, no. 1, pp. 29-33, Jan 2017, doi: 10.1136/neurintsurg-2016-012287.

[8] H. Luecking *et al.*, "FRED Flow Diverter: A Study on Safety and Efficacy in a Consecutive Group of 50 Patients," (in eng), *AJNR Am J Neuroradiol,* vol. 38, no. 3, pp. 596-602, Mar 2017, doi: 10.3174/ajnr.A5052.

[9] R. W. Ryan, A. S. Khan, R. Barco, and A. Choulakian, "Pipeline flow diversion of ruptured blister aneurysms of the supraclinoid carotid artery using a single-device strategy," (in eng), *Neurosurg Focus,* vol. 42, no. 6, p. E11, Jun 2017, doi: 10.3171/2017.3.Focus1757.

[10] C. Yang, A. Vadasz, and I. Szikora, "Treatment of ruptured blood blister aneurysms using primary flow-diverter stenting with considerations for adjunctive coiling: A single-centre experience and literature review," (in eng), *Interv Neuroradiol,* vol. 23, no. 5, pp. 465-476, Oct 2017, doi: 10.1177/1591019917720805.

[11] D. Kumar *et al.*, "Flow Diversion in Ruptured Blood Blister Aneurysms: Single Centre Experience," (in En), *Journal of Clinical Interventional Radiology ISVIR,* vol. 01, no. 02, pp. 077-084, 2017/07/28 2017, doi: 10.1055/s-0037-1602770.

[12] V. Hellstern *et al.*, "Microsurgical clipping and endovascular flow diversion of ruptured anterior circulation blood blister-like aneurysms," (in eng), *Interv Neuroradiol,* vol. 24, no. 6, pp. 615-623, Dec 2018, doi: 10.1177/1591019918785911.

[13] E. Lozupone *et al.*, "Flow diverter devices in ruptured intracranial aneurysms: a single-center experience," (in eng), *J Neurosurg,* vol. 128, no. 4, pp. 1037-1043, Apr 2018, doi: 10.3171/2016.11.Jns161937.

[14] M. Mokin *et al.*, "Treatment of blood blister aneurysms of the internal carotid artery with flow diversion," (in eng), *J Neurointerv Surg,* vol. 10, no. 11, pp. 1074-1078, Nov 2018, doi: 10.1136/neurintsurg-2017-013701.

[15] R. Parthasarathy, V. Gupta, and A. Gupta, "Safety of Prasugrel loading in ruptured blister like aneurysm treated with a Pipeline device," (in eng), *Br J Radiol,* vol. 91, no. 1086, p. 20170476, Jun 2018, doi: 10.1259/bjr.20170476.

[16] M. Ghorbani *et al.*, "Flow diverter embolization device for endovascular treatment of ruptured blister and wide necked very small aneurysms," (in eng), *Heliyon,* vol. 5, no. 9, p. e02241, Sep 2019, doi: 10.1016/j.heliyon.2019.e02241.

[17] A. Griffin *et al.*, "Predictors of Clinical Outcome After Treatment of Intracranial Aneurysms with the Pipeline Embolization Device," (in eng), *World Neurosurg,* vol. 130, pp. e666-e671, Oct 2019, doi: 10.1016/j.wneu.2019.06.185.

[18] R. Capocci *et al.*, "Delayed Treatment (≥5 Days) by Flow Diversion of Ruptured Blister-Like Cerebral Aneurysms : Case Series of 8 Consecutive Patients," (in eng), *Clin Neuroradiol,* vol. 30, no. 2, pp. 287-296, Jun 2020, doi: 10.1007/s00062-019-00758-4.

[19] F. Incandela *et al.*, "Flow diverting devices in acute ruptured blood blister aneurysms: a three centric retrospective study," (in eng), *Acta Biomed,* vol. 91, no. 10-s, p. e2020011, Sep 23 2020, doi: 10.23750/abm.v91i10-S.10261.

[20] M. A. Möhlenbruch *et al.*, "Treatment of Ruptured Blister-Like Aneurysms with the FRED Flow Diverter: A Multicenter Experience," (in eng), *AJNR Am J Neuroradiol,* vol. 41, no. 12, pp. 2280-2284, Dec 2020, doi: 10.3174/ajnr.A6849.

[21] P. Zhang *et al.*, "Flow Diverter-Assisted Coil Embolization of Blood Blister-Like Aneurysm Using Semi-deploying Technique," (in eng), *Frontiers in neurology,* vol. 11, p. 625203, 2020, doi: 10.3389/fneur.2020.625203.

[22] R. Aboukais *et al.*, "Ruptured blood blister like aneurysm: does the best therapeutic option really exist?," (in eng), *Neurosurg Rev,* vol. 44, no. 5, pp. 2767-2775, Oct 2021, doi: 10.1007/s10143-020-01463-4.

[23] A. Gopinathan *et al.*, "Flow Diversion in Acute Sub Arachnoid Haemorrhage: A Single Centre Five Year Experience," (in eng), *J Stroke Cerebrovasc Dis,* vol. 30, no. 8, p. 105910, Aug 2021, doi: 10.1016/j.jstrokecerebrovasdis.2021.105910.

[24] A. Tanburoglu and C. Andic, "Early Treatment of Ruptured Blood Blister-Like Aneurysms of the Internal Carotid Artery With Flow Diverters Using Single Antiplatelet Therapy: A Single-Center Experience With Long-Term Follow-Up," (in eng), *Frontiers in neurology,* vol. 12, p. 708411, 2021, doi: 10.3389/fneur.2021.708411.

[25] T. Wang *et al.*, "Cerebral vasospasm resulted in "stent shortening" after pipeline assisted coil embolization for blood blister aneurysms," (in eng), *Medicine (Baltimore),* vol. 100, no. 33, p. e26971, Aug 20 2021, doi: 10.1097/md.0000000000026971.

[26] J. Zhang, M. Yu, and X. Lv, "Endovascular treatment of blood blister-like aneurysms of internal carotid artery: Stent-assisted coiling and pipeline flow diversion," (in eng), *J Clin Neurosci,* vol. 90, pp. 8-13, Aug 2021, doi: 10.1016/j.jocn.2021.04.040.

[27] W. Zhong *et al.*, "Pipeline Embolization Device for the Treatment of Ruptured Intracerebral Aneurysms: A Multicenter Retrospective Study," (in eng), *Frontiers in neurology,* vol. 12, p. 675917, 2021, doi: 10.3389/fneur.2021.675917.

[28] K. Alpay *et al.*, "Finnish flow diverter study: 8 years of experience in the treatment of acutely ruptured intracranial aneurysms," (in eng), *J Neurointerv Surg,* vol. 14, no. 7, pp. 699-703, Jul 2022, doi: 10.1136/neurintsurg-2021-017641.

[29] P. Liu *et al.*, "Treatment of Blood Blister Aneurysms of the Internal Carotid Artery With Pipeline-Assisted Coil Embolization: A Single-Center Experience," (in eng), *Frontiers in neurology,* vol. 13, p. 882108, 2022, doi: 10.3389/fneur.2022.882108.

[30] W. Feng, X. Tian, J. Kang, Z. Han, and E. Chen, "Flow Diverter Device-Assisted Coiling Treatment for Cerebral Blister Aneurysm: A Single-Center Study," (in eng), *Brain Sci,* vol. 13, no. 3, Mar 3 2023, doi: 10.3390/brainsci13030435.

[31] J. Madjidyar *et al.*, "Single-antiplatelet regimen in ruptured cerebral blood blister and dissecting aneurysms treated with flow-diverter stent reconstruction," (in eng), *J Neurointerv Surg,* vol. 15, no. 10, pp. 953-957, Oct 2023, doi: 10.1136/jnis-2022-019361.

[32] J. Reidy *et al.*, "Endovascular and microsurgical management of blister aneurysms: a multi-centre review," (in eng), *Neurosurg Rev,* vol. 46, no. 1, p. 147, Jun 24 2023, doi: 10.1007/s10143-023-02065-6.

[33] Y. B. Zhang *et al.*, "Treatment with a flow diverter-assisted coil embolization for ruptured blood blister-like aneurysms of the internal carotid artery: a technical note and analysis of single-center experience with pooled data," (in eng), *Neurosurg Rev,* vol. 46, no. 1, p. 305, Nov 20 2023, doi: 10.1007/s10143-023-02216-9.

[34] J. E. Hoffman *et al.*, "Periprocedural management of ruptured blister aneurysms treated with pipeline flow diversion," (in eng), *Surg Neurol Int,* vol. 15, p. 73, 2024, doi: 10.25259/sni_482_2023.

[35] K. Chen *et al.*, "Comparison of safety and efficacy between double LVIS and flow diverter for Blister-Like aneurysms: a multicenter retrospective study," (in eng), *Sci Rep,* vol. 15, no. 1, p. 19809, Jun 5 2025, doi: 10.1038/s41598-025-05234-3.

[36] X. Chen, Z. Zhou, J. Gao, and J. Yu, "Treatment of blood blister-like aneurysms of the supraclinoid internal carotid artery using pipeline and lattice flow diverters and coiling," (in eng), *Frontiers in neurology,* vol. 16, p. 1607683, 2025, doi: 10.3389/fneur.2025.1607683.

[37] C. C. Chen *et al.*, "Single antiplatelet therapy and tirofiban bridged with surface modified flow diverters for ruptured blood blister-like aneurysms: single center experience and systematic review," (in eng), *J Neurointerv Surg,* Aug 14 2025, doi: 10.1136/jnis-2025-023832.

[38] N. N. Dange, M. Gharat, N. V. Naikwade, and K. Bhatia, "Endovascular Parent Artery Reconstruction for Ruptured Intracranial Blister Aneurysms: Demonstrating Safety and Efficacy After Early Flow Diversion," (in eng), *Cureus,* vol. 17, no. 12, p. e99536, Dec 2025, doi: 10.7759/cureus.99536.

[39] W. W. Wroe *et al.*, "Outcomes after flow diversion for ruptured blood blister-like and dissecting aneurysms: A single-center series," (in eng), *Interv Neuroradiol,* p. 15910199251398360, Nov 28 2025, doi: 10.1177/15910199251398360.
